# Mu-Suppression Neurofeedback Training Targeting the Mirror Neuron System: A Pilot Study

**DOI:** 10.1007/s10484-024-09643-4

**Published:** 2024-05-13

**Authors:** Samaneh S. Dastgheib, Wenbo Wang, Jürgen M. Kaufmann, Stephan Moratti, Stefan R. Schweinberger

**Affiliations:** 1 Department for General Psychology and Cognitive Neuroscience, Institute of Psychology, Friedrich Schiller University of Jena, Am Steiger 3/1, 07743 Jena, Germany; 2 Social Potential in Autism Research Unit, Friedrich Schiller University of Jena, Am Steiger 3/1, 07743 Jena, Germany; 3 Department of Experimental Psychology, Complutense University of Madrid, Madrid, Spain; 4 Center for Intervention and Research On Adaptive and Maladaptive Brain Circuits Underlying, Mental Health (C-I-R-C), Jena-Magdeburg-Halle, Germany; 5 German Center for Mental Health (DZPG), Jena-Magdeburg-Halle, Germany

**Keywords:** Mu Suppression, Mirror Neuron System, Neurofeedback, EEG

## Abstract

Neurofeedback training (NFT) is a promising adjuvant intervention method. The desynchronization of mu rhythm (8–13 Hz) in the electroencephalogram (EEG) over centro-parietal areas is known as a valid indicator of mirror neuron system (MNS) activation, which has been associated with social skills. Still, the effect of neurofeedback training on the MNS requires to be well investigated. The present study examined the possible impact of NFT with a mu suppression training protocol encompassing 15 NFT sessions (45 min each) on 16 healthy neurotypical participants. In separate pre- and post-training sessions, 64-channel EEG was recorded while participants (1) observed videos with various types of movements (including complex goal-directed hand movements and social interaction scenes) and (2) performed the "Reading the Mind in the Eyes Test" (RMET). EEG source reconstruction analysis revealed statistically significant mu suppression during hand movement observation across MNS-attributed fronto-parietal areas after NFT. The frequency analysis showed no significant mu suppression after NFT, despite the fact that numerical mu suppression appeared to be visible in a majority of participants during goal-directed hand movement observation. At the behavioral level, RMET accuracy scores did not suggest an effect of NFT on the ability to interpret subtle emotional expressions, although RMET response times were reduced after NFT. In conclusion, the present study exhibited preliminary and partial evidence that mu suppression NFT can induce mu suppression in MNS-attributed areas. More powerful experimental designs and longer training may be necessary to induce substantial and consistent mu suppression, particularly while observing social scenarios.

**Supplementary Information:**

The online version contains supplementary material available at 10.1007/s10484-024-09643-4.

## Introduction

Neurofeedback is an operant conditional learning approach based on a brain-computer interface (BCI), which has shown promising results in treating neurological and psychiatric disorders without significant side effects (Shindo et al., 2011; Sitaram et al., 2017; Young et al., 2017). This is a substantial advantage since pharmacological therapies usually result in adverse side effects (Kloosterboer et al., 2020; Sitaram et al., 2017). Another advantage of neurofeedback training (NFT) therapy is that it involves the active participation of the clients and their brains' neural networks. It can potentially induce long-term synaptic structural changes by facilitating neural plasticity resulting in long-term therapeutic effects. Animal studies have shown effects lasting longer than a week after the NFT was terminated, with only two training sessions (Sitaram et al., 2017). Human studies in attention deficit hyperactivity disorder (ADHD), stroke, and epilepsy have also provided evidence for positive effects (Haut et al., 2019; Trambaiolli et al., 2021; Van Doren et al., 2019). However, even though neurofeedback has been suggested as a promising adjuvant therapy, it is also sometimes discussed controversially (Cortese et al., 2016; Sitaram et al., 2017). It is reported that even with repeated sessions, up to 30% of human NFT participants may fail to regulate their brain activity. Overall, more systematic research on the efficacy of experimental and clinical NFT protocols is needed to elucidate the factors influencing them. More randomized control trials are needed to confirm protocol-specific effects (Jackson et al., 2006; Sitaram et al., 2017).

Notwithstanding its critical reception, neurofeedback, as a non-pharmacological intervention, presents a prospective therapeutic avenue for conditions where conventional medical treatments have demonstrated limited efficacy, such as autism. Regarding the treatment of autism, medical therapy is mainly reduced to treating symptoms, and the affected individuals usually end up taking multiple medications, often with disturbing side effects, even though no prescription has been approved for the core symptoms of autism so far (McPheeters et al., 2011; Pandina et al., 2020). Autism spectrum disorders (ASD) are characterized by social communication and repetitive movements (American Psychiatric et al., 2013), with theories suggesting impairments in the "theory of mind" linked to MNS dysfunction, motor clumsiness, and imitation difficulties. (Coben et al., 2014; Oberman et al., 2005).

The concept of the MNS was introduced by Giacomo Rizzolatti and his group (Gallese et al., 1996), who first described mirror neurons (MNs) in the macaque brain as groups of neurons that fire during the performance of a specific goal-directed action (e.g., grasping food). Crucially, these neurons in premotor and parietal regions show strikingly similar firing while merely observing such actions in others. Interestingly, a subgroup of mirror neurons, the so-called congruent neurons, do not only fire when the action perceived is precisely identical to the performed one, but also when a similar action serves the same goals (Rizzolatti & Craighero, 2004). Moreover, mirror neurons can fire with patterns that resemble those of anticipated actions. For instance, when expecting goal-directed actions (e.g., taking food into the mouth) that would follow an observed action (e.g., putting food on a table), mirror neurons can fire with patterns similar to an observation of the expected action (Iacoboni & Dapretto, 2006). In sum, MNs code abstract representations of observed actions, extracting an observer's intention. This embodied experience of understanding others' actions links sensation and action and produces a model of the outside world by mirroring it from within.

The main areas assumed to encompass MNs in humans are the inferior frontal cortex, enclosing the posterior inferior frontal gyrus (IFG), the adjacent ventral premotor cortex, and the superior parts of the inferior parietal lobule (IPL) with the superior temporal sulcus as their primary visual input (Iacoboni & Dapretto, 2006). The posterior inferior frontal cortex in humans and monkeys is strongly connected to the inferior parietal lobule, constituting parallel fronto-parietal neural systems belonging to various known networks (Caspers et al., 2012). Thus, the MNS should be understood within the broader context of the massive fronto-parietal networks for sensorimotor integration. It consists of multiple body representations in the frontal areas that participate in complex movement planning and execution (like using tools) in cooperation with parietal areas, e.g., the rostral parts of the inferior parietal lobule (Iacoboni & Dapretto, 2006; Rizzolatti et al., 2014). When perceiving an action performed by somebody else, the perceiver's MNS activates the internal neural code of a corresponding own action (Rizzolatti & Craighero, 2004; Umilta et al., 2001). Such neural mirroring can promote action prediction skills in human infants (Falck-Ytter et al., 2006; Marshall & Meltzoff, 2011), which may be relevant for complex social and communicative abilities (Alcala-Lopez et al., 2019). Finally, putative impairments in the human MNS could offer insights into underlying mechanisms and, therefore, potential interventions for various disorders, including autism spectrum disorders.

Alongside extensive research on the MNS and its function, there is an ongoing search for non-invasive biomarkers reflecting human MNS activation. Event-related desynchronization (ERD) and synchronization (ERS) have long histories as non-invasive methods to investigate brain physiology. Since Hans Berger (Berger, 1929), it is known that certain events can block or desynchronize ongoing EEG oscillations (e.g. alpha rhythms). These EEG oscillations exhibit event-related characteristics and temporal synchronization, yet they are not strictly stimulus-bound or precisely synchronized with a stimulus onset. Moreover, they are subject to modulation by the brain's feedback loops, encompassing thalamocortical and cortico-cortical circuits. This renders Event-Related Desynchronization/Event-Related Synchronization (ERD/ERS) as apt candidates for the investigation of cerebral networks (Pfurtscheller & da Silva, 1999). Along these lines, there has been growing interest in mu rhythm as the potential biomarker for human neural mirroring (Fox et al., 2016; Oberman et al., 2005; Pineda et al., 2008). Mu rhythm reflects EEG oscillations within the alpha frequency range of ~ 8–13 Hz in adults (~ 6–9 Hz in children). Unlike alpha, mu is mainly recorded from central rather than occipital sites. Mu amplitude is largest at rest and smallest during action execution. Importantly, mu is compatible with MNS function, in that it is also suppressed when the participant merely observes another person's action (Avanzini et al., 2012; Fox et al., 2016; Lepage & Theoret, 2006; Muthukumaraswamy & Johnson, 2004). The idea that mu suppression qualifies as an MNS activity biomarker is reinforced by findings that the typical mu suppression during action observation is reduced or eliminated in individuals with autism (Oberman et al., 2005).

Given the link between autism and MNS dysfunctions (Iacoboni & Dapretto, 2006), it is reasonable to assume training mu rhythm activity via NFT could target social communications in Autism Spectrum Disorder (ASD). Jaime Pineda and his group (e.g., LaMarca et al., 2023; Oberman et al., 2005; Pineda et al., 2008) investigated this hypothesis through a series of experiments. The mu suppression index (MSI) developed by this team (Oberman et al., 2005; Pineda et al., 2008) showed that mu suppression was preserved during self-performed hand movements in ASD individuals, but not during the observation of others' actions (Oberman et al., 2005), suggesting MNS dysfunction in ASD.

Pineda and colleagues (Pineda et al., 2008) developed a social-specific neurofeedback protocol for autism by training the individuals' mirror neuron system (MNS). Specifically, they tried to modulate mu rhythm using NFT. Note that, in most studies, Pineda et al. counter-intuitively trained for mu enhancement, rather than suppression (Datko et al., 2018; Pineda et al., 2008; Pineda et al., 2014; but see also Friedrich et al., 2015). Although the overarching aim was to provide participants with a degree of control over their mu rhythm, this may be surprising, because it is mu suppression that appears to be linked to the activation of the mirror neuron system.

We explored, whether higher task-dependent mu suppression can be achieved following a mu-suppression NFT protocol. For this, we recruited 16 neurotypical individuals and trained them using a similar neurofeedback protocol as utilized by Pineda et al. (2014), except that we trained mu suppression instead of mu enhancement. To assess NFT effects, we recorded 64-channel EEG while participants observed biological and non-biological actions, in two separate sessions, before and after NFT. In these sessions, participants watched several types of videos, including moving balls (baseline), simple and complex hand movements, and social interaction scenarios. The ERD in the mu frequency band was derived from the EEG data and the implicated brain areas were estimated using source reconstruction analysis. We expected that NFT effects would be modulated by the type of scenario observed. Specifically, we expected the most prominent mu suppression during the observation of hand movement actions (also cf. Fox et al., 2016; Oberman et al., 2005).

## Method

### Participants

Sixteen healthy neurotypical volunteers (three males and 13 females; age 19–25 years; *M* = 21.5, *SD* = 2.0) participated in the study and contributed data. We determined sample size using GPower 3.1 (Faul et al., 2007) based on a previously reported effect size (Pineda et al., 2014; ŋ_p_^2^ = 0.43). In order to detect condition difference using a repeated measures ANOVA (one group, and three measures representing the three conditions), 11 participants would be necessary to achieve a power of 0.80). Because of possible dropouts, we recruited 16 participants. One additional female participant dropped out before the start of the training sessions for personal reasons. Participants were university students of psychology (*n* = 14) or engineering (*n* = 2), and they received course credit or a small financial reimbursement for participation. Exclusion criteria were applied if participants reported diagnoses with neurological or psychiatric disorders or taking medications or drugs that affected the nervous system; two further participants were excluded accordingly (one who reported a diagnosis of ADHD, and one who reported occasional marijuana consumption). The procedure was fully explained to every participant, and all of them gave written informed consent. The procedure followed the declaration of Helsinki, and the faculty's ethics committee approved the study (Reg. No. 2018–1156-BO). All participants completed the autism-spectrum quotient questionnaire (Freitag et al., 2007) to exclude participants with an autism diagnosis. None of the participants attained a score surpassing the suggested threshold of 29 and thus none was excluded due to high autistic traits (*M* = 13.75, *SD* = 4.6).

### Behavioral Experiment

The Reading the Mind in the Eyes test (RMET) (Baron-Cohen et al., 2001) was used before and after NFT. We evaluated response times and accuracy. Using paired t-tests, pre- versus post-training differences were evaluated.

### EEG Experimental Procedures

#### Experimental Conditions and Stimuli

Two sessions of EEG assessments were performed, one before and one after NFT, during which participants watched four video categories. These encompassed video clips showing a) Non-biological movements (moving balls), b) Non-goal directed biological actions (simple hand movements) – subsequently termed as *Simple condition*, c) Goal-directed biological actions (complex hand movements) – subsequently termed as *Complex condition*, and d) Social interaction scenarios – subsequently termed as *Social condition*. As detailed below, the Mu Suppression Index (MSI) was used to assess changes in mu (8–13 Hz) power in response to the observation of these videos. For every category, five video clips (each showing a different exemplar of movement, but from the same category) were edited using Adobe Premiere Pro (CS6 version 6.0.0), with a final size of 1920*1080 pixels and 30 frames per second, each lasting 24 s. In total, this resulted in 4 clips of 2 min, one for each condition (category; 5 × 24 s). The five videos of the non-biological category (showing moving balls) were downloaded from a free licensed website (for details please see the associated OSF repository), and edited according to the abovementioned standards. For the other three categories, we created the stimuli using a high-definition video camera (Panasonic HC-V777EG-K). These included Simple hand movements (e.g., a hand opening and closing rhythmically every second), Complex goal-directed hand movements (e.g., right hand taking a bar of chocolate among many, or using a hammer), and Social interaction scenarios of three people (e.g., when playing interactively with a ball). All videos were in color. Hand movement videos were taken in our audiovisual lab, and social interaction videos were recorded outdoors (see the supplementary materials for more details about the stimuli and their production). Videos were presented with EPrime™ (version 3.0) in the center of a 19″ color monitor and at a screen resolution of 1280 by 1024 pixels. Using a chin rest, the viewing distance was fixed to 110 cm, and video sizes were 34 cm by 22 cm, resulting in viewing angles of 8.78° by 5.71°. To perceptually validate the stimuli set, two online pilot experiments were performed (results and statistical analysis are uploaded as supplementary materials on the OSF repository).

**Fig. 1:**
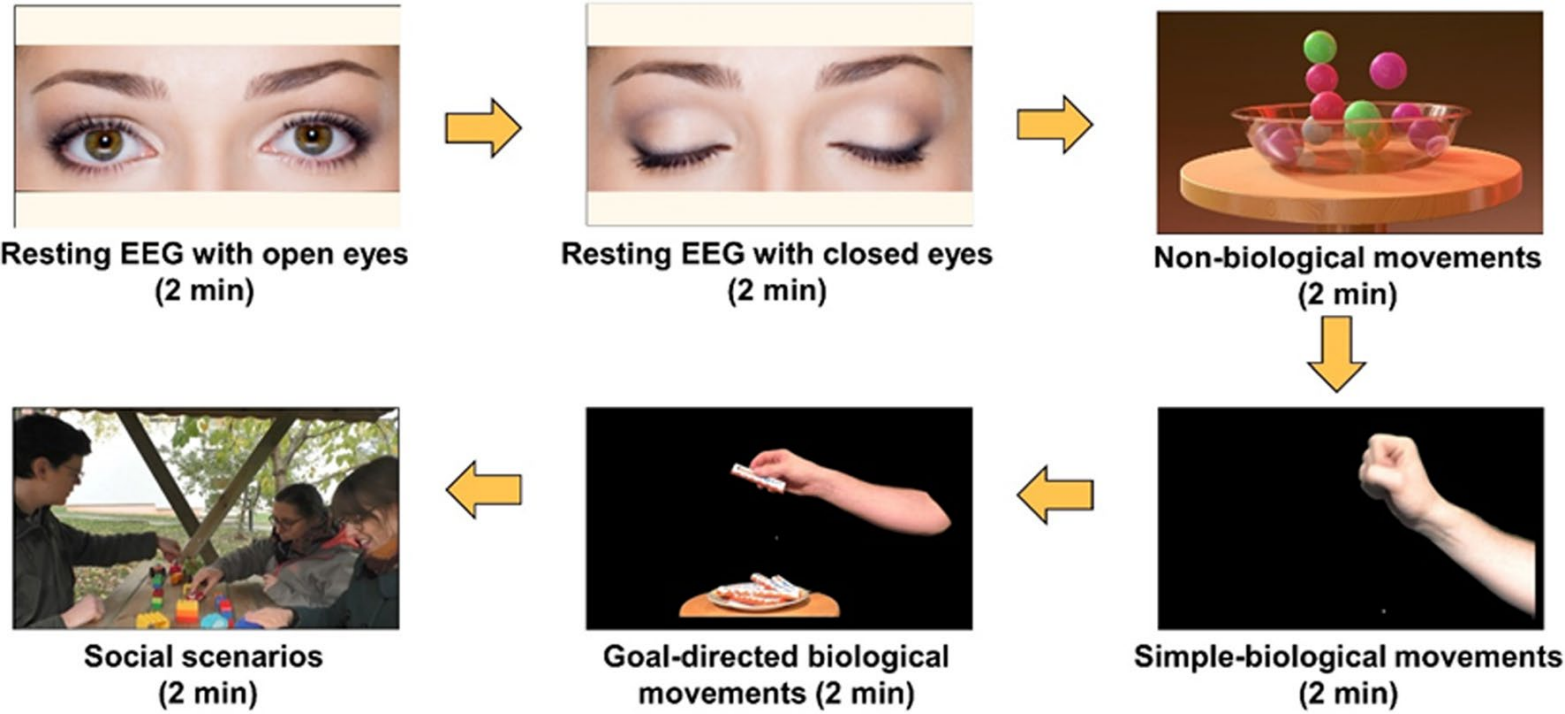
Schematic illustration of blocks in the EEG experiment. The arrows show the order of blocks and conditions

The EEG was recorded during the observation of the described videos and during two resting conditions – one with open eyes while looking at a black screen on the monitor and one with closed eyes. Each resting condition lasted for 120 s. The order of blocks was fixed. See Fig. 1 for a schematic illustration of the experimental design.

#### Electrophysiological Recordings and Pre-Processing

For the EEG part of the experiment, participants were seated in a dimly lit, electrically shielded, and sound-attenuated with their heads in a chin rest. Electroencephalographic data were recorded with 64 BioSemi™ (BioSemi, Amsterdam, Netherlands) Active Two-System sintered Ag/AgCI electrodes attached to an elastic electrode cap (Electro-Cap Intl.™). The electrode layout ensured adequate coverage according to the extended international 10–20 system at the positions FP1, FT9′, AF3, F1, F3, F5, F7, FT7, TP9′, FC3, FC1, C1, C3, C5, T7, TP7, PO9′, CP3, CP1, P1, P3, O9′, P7, P9, PO7, PO3, O1, Iz, Oz, POz, Pz, CPz, FPz, FP2, FT10′, AF4, AFz, Fz, F2, F4, F6, F8, FT8, TP10′, FC4, FC2, FCz, Cz, C2, C4, C6, T8, TP8, PO10′, CP4, CP2, P2, P4, O10′, P8, P10, PO8, PO4, and O2. Rather than ground and reference electrodes, the BioSemi™ system utilizes a CMS/DRL feedback loop (see http://www.biosemi.com/faq/cms&drl.htm) with two additional electrodes. Horizontal electrooculogram (EOG) electrodes were placed on the outer canthi of both eyes, and vertical EOG was recorded from two electrodes placed above and below the right eye. Data were continuously recorded and amplified with a BioSemi™ amplifier and digitally recorded with ActiView™ (version 6.0.5), with online filtering (DC to 120 Hz, low-pass) and a sampling rate of 512 Hz. Noisy segments due to movement artifacts and horizontal eye movements as monitored by the EOG were determined by visual inspection and excluded from the analysis. Eye blink artifacts were removed from data by Signal-Space Projection (SSP) implemented in Brainstorm toolbox (https://neuroimage.usc.edu/brainstorm) (Uusitalo & Ilmoniemi, 1997).

#### Data Processing and EEG Analysis

EEG data were processed using the Brainstorm toolbox for MATLAB (Tadel et al., 2011). After removing the DC offset, data were re-referenced to average reference and segmented into 20 s epochs (for action videos) and 100 s epochs for the resting conditions. The mu frequency band overlaps with the alpha band, which is usually picked up at posterior sites. Since the generator for posterior alpha is more robust than the one for mu (Oberman et al., 2005), posterior alpha activity might affect recordings from C3, Cz, and C4. As posterior alpha is sensitive to the transitions from one stimulus to the other, we excluded EEG data corresponding to the transitions between the individual 24 s video clips within the same video category from the analysis. Specifically, we excluded the first and last 2 s of each EEG block for action videos, and the first and last 10 s of each EEG block for open/closed resting conditions. Thus, for action videos, a 20-s segment of data was obtained and combined with the other segments of the same condition (i.e. five 20 s epochs per condition), resulting in a 100 s segment of data per condition. Finally, data sets were evaluated for the power of EEG signals at different frequencies, using Welsch’s method. Thereby, overlapping (50%) 2-s hamming windows were moved across the 100 s segments. For each moving window the power of the Fourier components using the Fast Fourier Transform (FFT) was determined. Then, these power estimates were averaged in order to determine the power spectrum density (PSD).

We calculated the mu suppression index (MSI) using the ratios of mu rhythm (mean PSD between 8–13 Hz) power during the *Simple*, *Complex*, and *Social* conditions relative to the mu power in the non-biological motion condition (moving balls). Note that we chose non-biological motion as the baseline because we expected different mu responses to biological movements, according to the properties of the mirror neuron system. A ratio was used to control for variability in absolute mu power as a result of individual differences in irrelevant aspects, e.g., scalp thickness and electrode impedance. A log transform was used for statistical data analysis because ratio data are inherently non-normally distributed. Thus, a log ratio of less than zero indicates mu suppression, whereas a value of zero indicates no change, and positive values mean mu enhancement in the specific experimental condition compared to the baseline.

$$\mathrm{MSI}\;\mathrm{in}\;a\;\mathrm{specific}\;\mathrm{scenario}\;\left(\mathrm{experimental}\;\mathrm{condition}\right)={\text{log}}_{10}\frac{Mu\;power\;during\;specific\;scenario}{Mu\;power\;during\;non-biological\;motion\;condition}$$

T-tests against zero were performed on the average MSI for every scenario, before and after neurofeedback training to statistically test whether the means differed positively or negatively from zero; in other words, to check whether mu enhancement or suppression had occurred during the observation of a specific experimental condition.

A repeated measure analysis of variance (ANOVA) was used to compare MSIs, including the factors session (before and after NFT), experimental condition (*Simple* vs. *Complex* vs. *Social* conditions), and electrode (C3 vs. C4 vs. Cz). A further reason for using these electrodes for frequency analysis was that mu rhythm can be prominently recorded from central areas (Fox et al., 2016; Pineda et al., 2014). Two of these electrodes (C3 and C4), were the target electrodes during NFT.

All the statistical analyses were performed using R version 4.0.4 (2021–02-15) (R Development Core Team, 2010), and all scripts plus the stimuli are available on OSF (https://osf.io/n5wrk/?view_only=873a011e9b65461eaafdc9370817a9e9).

#### Source Reconstruction Analysis

For cortical source estimation we used the default anatomical head model included in Brainstorm (ICBM125 2009c). Before forward modeling, the electrode locations were co-registered with the canonical template brain using a nonlinear warping algorithm implemented in Brainstorm. The forward model was calculated using a boundary element model (BEM). As we had few trials and a non-conventional baseline, we used the identity matrix as noise covariance estimate, assuming constant noise across electrodes. For the inverse solution a weighted minimum norm (wMNE) approach estimated the current density distribution underlying the recorded EEG. Then, the same power spectrum density and MSI calculations as in the electrode space were performed at each source vertex of the canonical template brain. Current source density MSI maps (for the Simple, Complex, and Social condition) were compared before and after NFT using paired t-tests at each vertex of the canonical brain surface. To safeguard against false positives due to multiple comparisons, clusters were formed based on these t-tests, with a two-sided cluster threshold of *p* = 0.05. Then, the same procedure was repeated 1,000 times, and at each repetition, the current density MSI contrasts were permuted between experimental conditions under the null hypothesis that the differences between the MSI maps of the two conditions were zero. The highest *t* cluster sum was entered into a permutation distribution at each repetition. Only *t* clusters of the initially observed contrast that formed cluster sums located below the 2.5 percentile or above the 97.5 percentile (two-sided test) of the cluster-based permutation distribution were considered significant (Nichols & Holmes, 2002). Detailed analyses and R scripts are available on OSF.

### Neurofeedback Training

All participants completed 15 sessions of 45-min NFT (i.e., approximately 11.25 h of training each), using a THERA PRAX® Q-EEG—Bio- and Neurofeedback System. The instructions were to sit calmly, avoid movements, and focus so that a self-selected video would keep playing smoothly rather than freeze. Thus, the video provided the incentive and feedback for whether or not participants succeeded in producing the target state. For the video to play smoothly, we selected mu rhythm (8–13 Hz) plus three additional frequency bands – theta (4–7 Hz), beta (14–30 Hz), and high beta (20–40 Hz) – which had to be maintained below pre-determined thresholds for at least 1 s.^1^ Our electrode arrangement consisted of five sintered Ag/AgCl ring electrodes: two active electrodes on C3 and C4 (overlying the brain's premotor region), and two electrodes on the right and left mastoid processes of petrosal bone (the reference was an averaged reference between the two), and one prefrontal electrode placed above the glabella bone as ground. The device was programmed to provide training based on the sum of signals recorded from C3 and C4 electrodes, which we refer to as C3-C4 montage in this text. The EEG from each active electrode was recorded, C3 and C4 were recorded separately, but training was based on the C3-C4 montage. The video would become slow and noisy when the mentioned rhythms exceeded thresholds. In order to resume playing, participants had to focus and maintain the levels of these frequencies below the thresholds for at least one second. The thresholds for the four frequency bands were determined in an initial *baseline period* (1 min) at the beginning of each session and were calibrated such that performance for the entire session fell in the 75–80% success range in the first seven sessions, and 65–75% in the last seven NFT sessions. As there are typical mu rhythm suppression associations with higher MNS activity (Iacoboni & Dapretto, 2006) and beta and theta frequencies with distraction (Pineda et al., 2014), suppression of these frequency bands was also trained. The THERA PRAX device registered the average amplitude for mu rhythm recorded from C3 and C4 montages separately and C3-C4 montage mu signal, C3-C4 montage theta, C3-C4 montage beta, and finally, C3-C4 montage gamma signals, which will be called in this text as montages, for baseline and feedback states per session, providing the possibility of tracking the training across sessions. The data recorded during neurofeedback sessions were also available based on training montages, feedback, and baseline period. Therefore, repeated measures ANOVAs were used to evaluate these data for the gradual effect on feedback or baseline period across the neurofeedback training session.

## Results

### Behavioral Study Results (RMET) Before and After NFT

Results of the reading minds in the eyes test showed no significant difference for accuracy (proportion correct responses), *M*_*before*_ = 0.734, *M*_*after*_ = 0.743, *t*(15) = 0.33, *p* = 0.74, *d* = 0.08, 95% CI for the difference [-0.06, 0.05]. Response times were significantly shorter after neurofeedback training, *M*_*before*_ = 7109 ms, *M*_*after*_ = 6046 ms, *t*(15) = 3.9, *p* = 0.001, *d* = 0.97, 95% CI for the difference [480, 1644].

### EEG Experiment Before and After NFT

#### Frequency Analysis

**Fig. 2:**
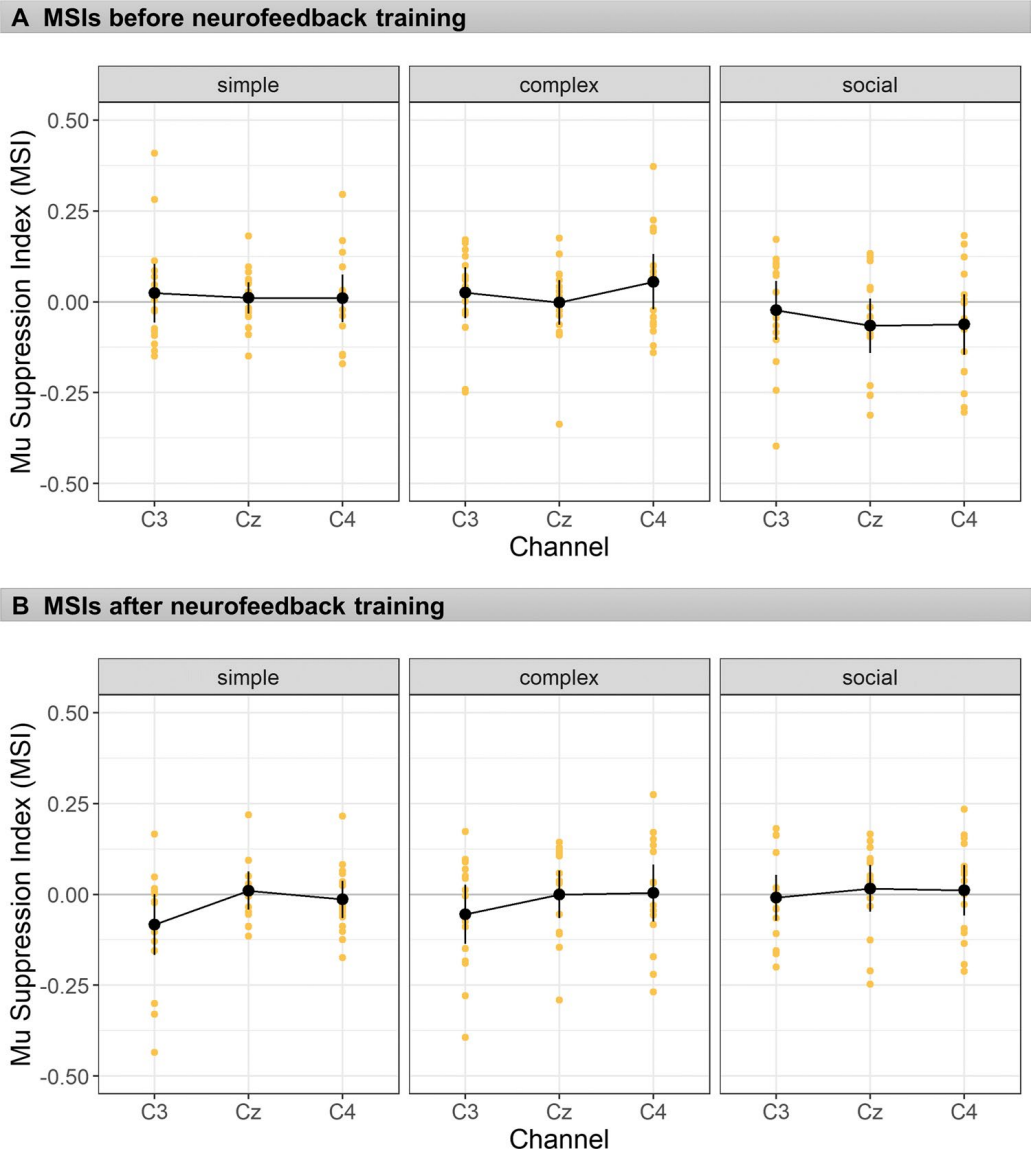
MSIs before and after neurofeedback training. Mu suppression indices (MSIs) before (top) and after neurofeedback training (bottom), separately for experimental conditions and electrodes. Error bars depict 95% confidence intervals, and the dots show the individual data points

MSIs were calculated as outlined above. Before NFT, there was neither suppression nor enhancement of mu rhythm during the hand movement observation conditions (Simple or Complex) or Social scenarios. In response to goal-directed biological movements (Complex condition), although the mean across the three electrodes was numerically higher than zero, *M* = 0.026, *SD* = 0.15, this difference also was far from significance, *t*(15) = 0.97,* p* = 0.35, *d* = 0.24, two-tailed, as was the difference in response to the Simple condition, *M* = 0.015, *SD* = 0.15,* t*(15) = 0.59*, p* = 0.56, *d* = 0.15, two-tailed. During the Social condition, although the mean was numerically negative, mu suppression was not significant, *M* = -0.05, *SD* = 0.14, *t*(16) = -1.5,* p* = 0.15, *d* = 0.38, two-tailed). After NFT, the frequency analysis again showed neither significant mu suppression nor enhancement (Complex condition: *M* = -0.017, *SD* = 0.14, *t*(15) = -0.63*, p* = 0.54, *d* = 0.16; Simple condition: *M* = -0.029, *SD* = 0.15,* t*(15) = -1.19*, p* = 0.26, *d* = 0.30; Social condition: *M* = 0.006, *SD* = 0.13,* t*(15) = 0.24*, p* = 0.81, *d* = 0.29). MSIs before and after NFT are depicted in Fig. 2.

In addition to testing MSIs against zero, a repeated measures ANOVA was also employed to compare the pattern of MSIs for the three factors of session (before and after NFT), experimental condition (Simple vs. Complex vs. Social), and electrode (C3 vs. C4 vs. Cz). The results revealed no significant main effects of either session, condition, or electrodes, all *F* values < 1. The two way interaction between session and condition, *F*(2,30) = 7.40, *p* = 0.002, *η*_*p*_^*2*^ = 0.33, and the interaction between electrode and session, *F*(2,30) = 3.44, *p* = 0.045,* η*^*2*^_*p*_ = 0.19 were statistically significant. Figure 2 suggests that the interaction between session and condition could be mainly due to the Social condition, because it tended to exhibit a degree of mu suppression before, but not after NFT. However, separate analyses for condition effects before NFT, *F*(2,30) = 2.78, *p* = 0.077,* η*^*2*^_*p*_ = 0.16, or after NFT *F*(2,30) = 1.62, *p* = 0.215,* η*^*2*^_*p*_ = 0.10, did not reveal any statistically significant condition differences within each session.

#### Source Reconstruction and the results of the Permutation T-Tests

##### *Simple* condition

**Fig. 3:**
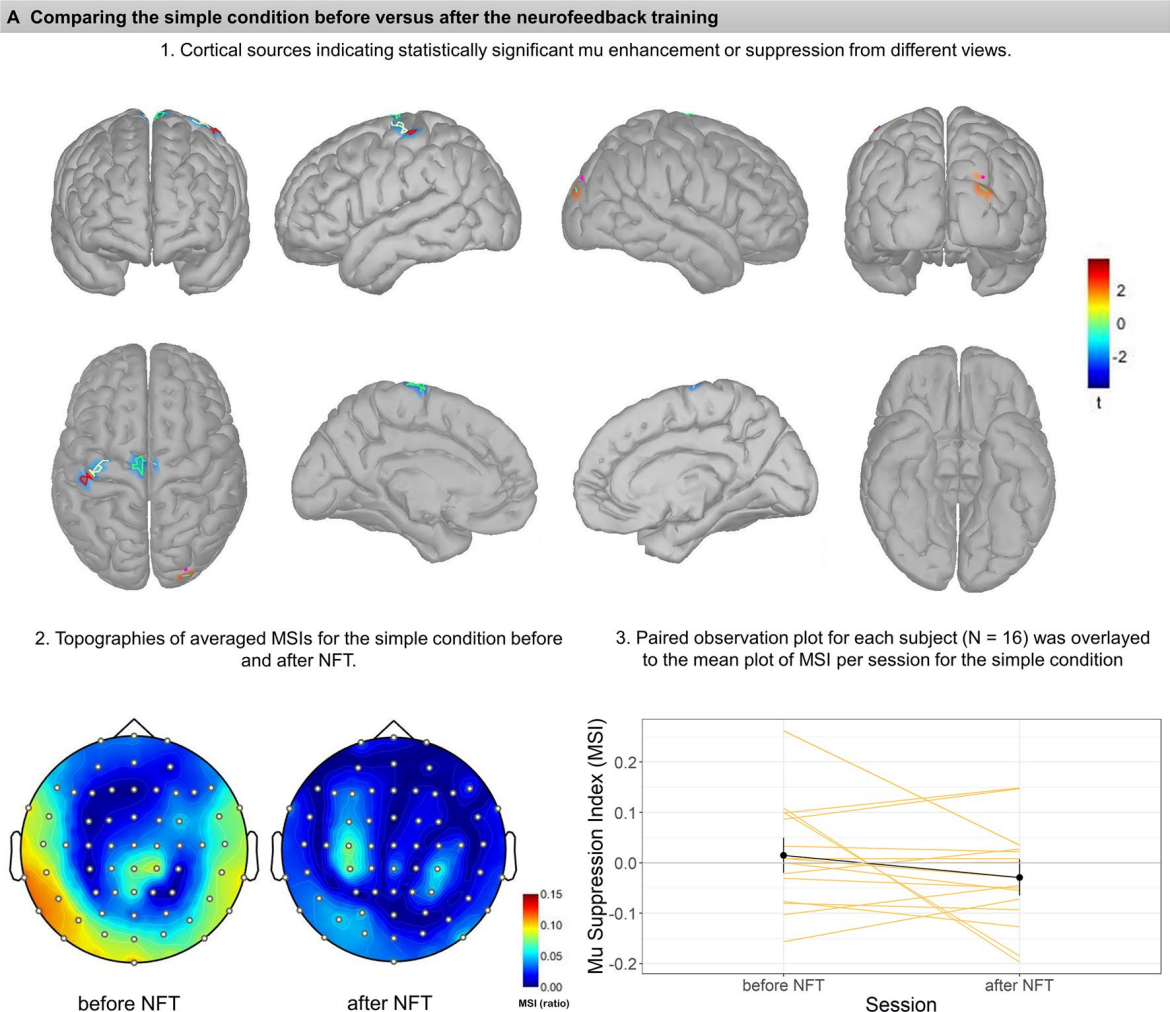

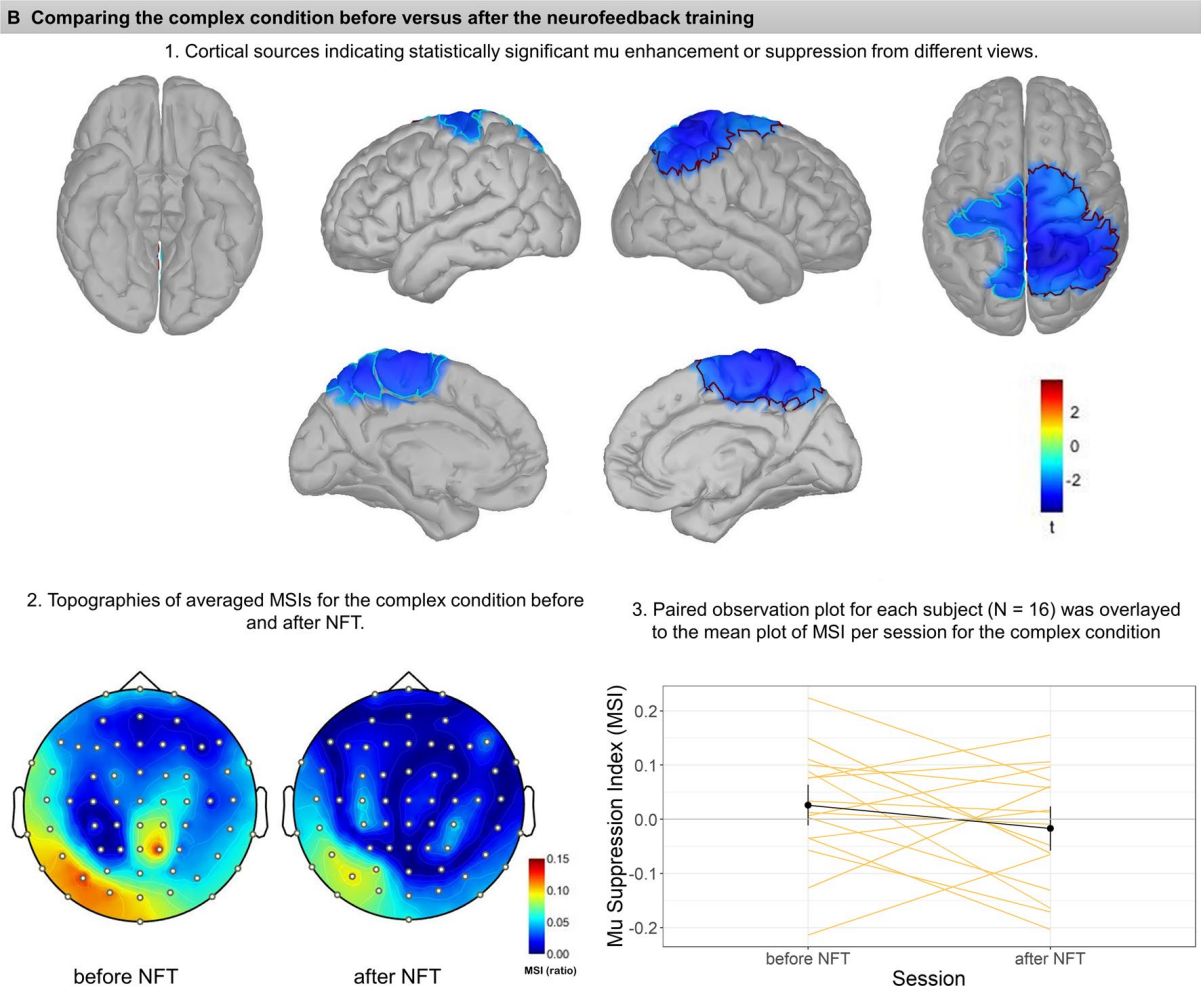

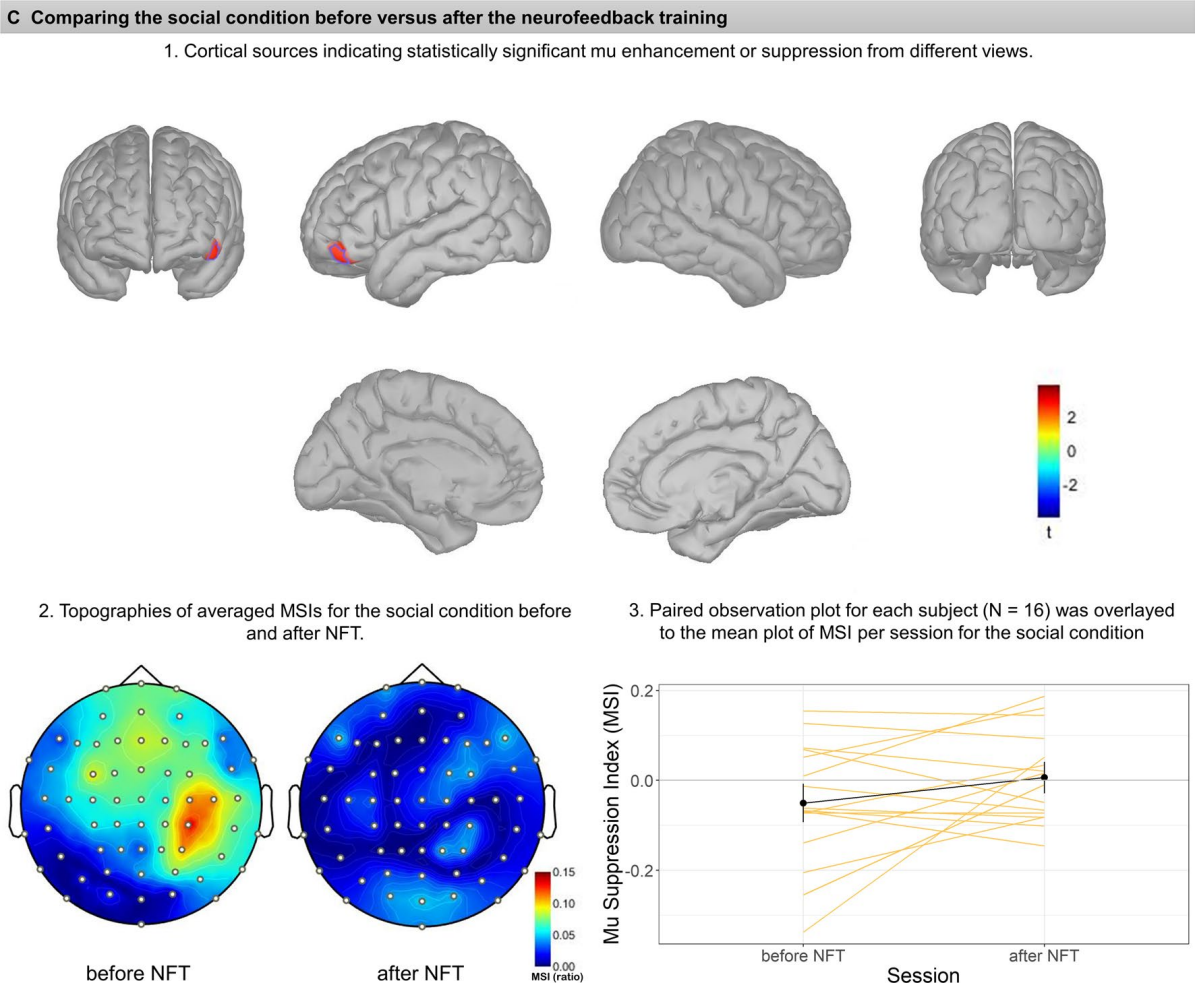
Each panel (**A**-**C**) illustrates the comparison before versus after neurofeedback in the Simple (**A**), Complex (**B**), and Social condition (**C**). Subsections (1–3) within each panel depict: (1) cortical sources resulting from permutation statistics, indicating statistically significant mu enhancement or suppression. Color bars indicate the t values, with blue colors showing lower MSIs after vs. before NFT (i.e., relative mu suppression after NFT), whereas red colors show mu enhancement after NFT vs before NFT (i.e., relative mu enhancement after NFT). Note that for comparison, each cluster is marked and bordered with a color corresponding to the cluster description and statistics in Table 1. (2) Averaged topographies of MSIs for each condition before and after NFT. Maps show spherical spline interpolation, with a 115° equidistant projection. (3) Paired observation plots per participant from the frequency analysis, with the means also indicated in black. Note that corresponding MSIs for the same participant are connected with lines

The permutation t-tests resulted in six small clusters (cf. Table 1) showing significant mu suppression or enhancement comparing the MSIs during observing simple hand movements before and after NFT (Fig. 3A). Areas on the anterior superior parietal lobule and lateral side of post-central gyrus on the left hemisphere showed suppression after NFT, *t*(15) = -1.80, *p* = 0.039, and* t*(15) = -1.80,* p* = 0.044, respectively. The superior areas of the left and right post-central gyri also showed significant mu suppression, *t*(15) = -2.14,* p* = 0.035, for the right, and *t*(15) = -1.92,* p* = 0.05, for the left cluster. However, two small posterior clusters on the anterior and posterior sides of the right calcarine sulcus showed enhancements, *t*(15) = 2.26,* p* = 0.038, and *t*(15) = 2.16,* p* = 0.05, respectively.

**Table 1:**
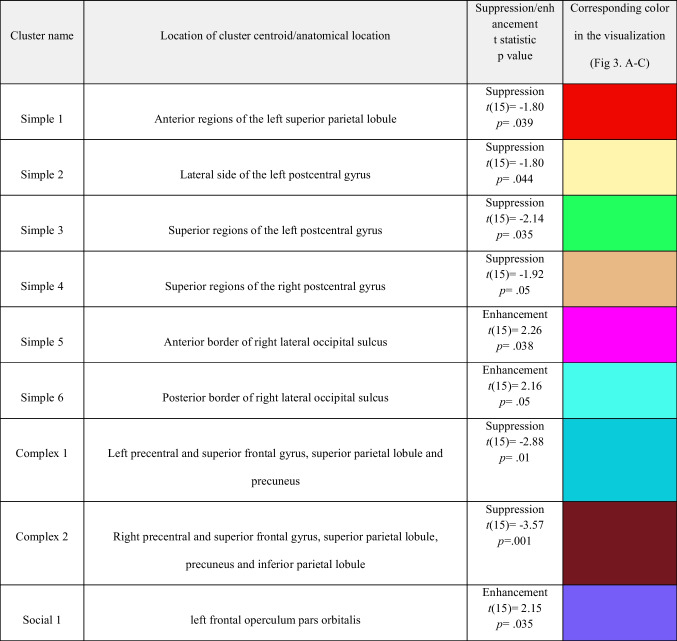
Significant clusters according to permutation t-tests comparing sessions before vs. after neurofeedback training, their estimated anatomical location, and t-test results showing either mu suppression or enhancement. Also see Fig. 3 for correspondence

##### *Complex* condition

The comparison for complex hand movements before and after neurofeedback yielded two vast clusters of mu suppression after NFT (Fig. 3B). On the left, the cluster includes superior areas of the pre-central gyrus and superior frontal gyrus, lateral side of the pre-central gyrus, superior border of the superior parietal lobule, which extends medially to paracentral lobule and the anterior 2/3 of precuneus, *t*(15) = -2.88,* p* = 0.01. The cluster on the right hemisphere was even more extensive and encompassed parts of the superior frontal and pre-central gyrus, superior parietal lobule, and superior regions of the inferior parietal lobule and supramarginal gyrus. Medially this cluster extended to the paracentral lobule and precuneus, *t*(15) = -3.57, *p* = 0.001.

##### *Social* condition

Regarding comparing social interaction scenarios before and after neurofeedback training, a small cluster positioned at the left frontal operculum pars orbitalis showed statistically significant mu enhancement after training, *t*(15) = 2.15, *p* = 0.035; see also Fig. 3C.

The MSIs in the source space were calculated using the same mathematical formula as in the sensor space. Table 1 shows a list of all clusters with their detailed neuroanatomy location and their corresponding color on graphs presented in Fig. 3 (A-C).

### Neurofeedback Sessions

**Fig. 4:**
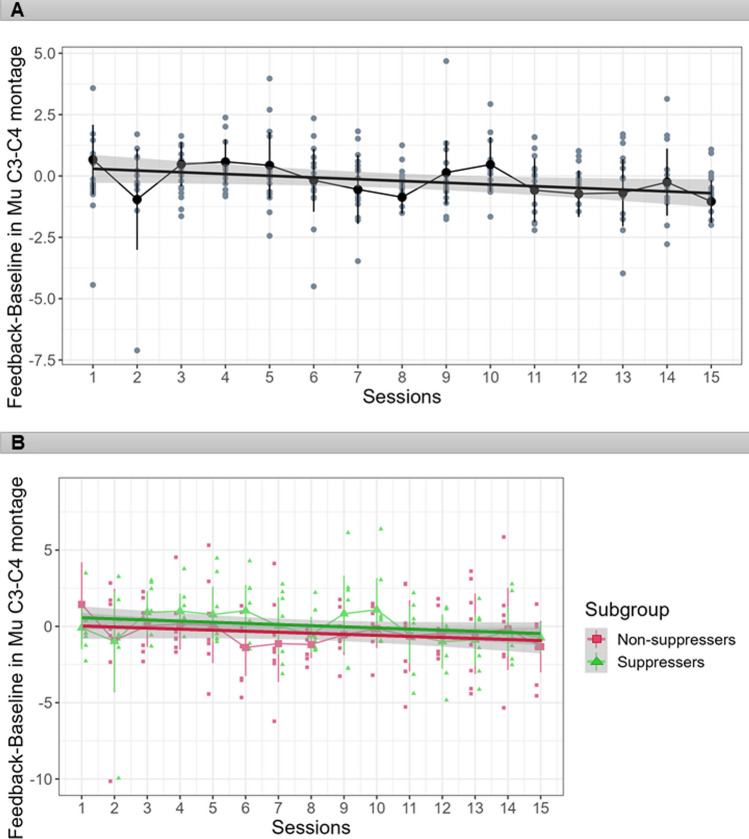
Each panel (**A** and **B**) presents mu amplitude difference (in microvolts) between feedback and baseline across the 15 neurofeedback sessions (**A**) in all participants, and (**B**) in two subgroups of suppressers, who showed mu suppression before NFT versus non-suppressers who did not show mu suppression before the training start. Note highly parallel neurofeedback training courses over sessions for both subgroups. The dots depict the individual data of the averaged mu amplitude during the feedback condition minus the baseline condition. Error bars show 95% confidence intervals, and the linear regression line is surrounded by the 95% confidence interval (the grey area)

As mentioned in the method section, the THERA PRAX device recorded the average amplitude of the signal from the C3-C4 montage for each four frequency bands in two feedback conditions, either baseline or feedback. We conducted an initial repeated measures ANOVA on these neurofeedback data, with the factors session (15 levels), frequency band (4 levels) and feedback condition (Baseline, Feedback), and applied Greenhouse–Geisser corrections for any violations of sphericity assumptions, as suggested by Mauchly´s tests. This analysis revealed no effect of session overall, *F*(5.68,85.19) = 1.08, *p* = 0.381, *η*^*2*^_*p*_ = 0.07, but a trend for an interaction between session and frequency band, *F*(42,630) = 1.40, *p* = 0.051, *η*^*2*^_*p*_ = 0.09. Refer to Fig. 4, which seems suggestive of a small numerical trend for suppression across training sessions in the mu band (but see below for qualification). Figure 4A, shows the mu amplitude difference between feedback and baseline in every NFT session (for the three other trained frequency bands, please refer to the supplementary material). There was also a main effect of the feedback condition, *F*(1,15) = 16.04, *p* = 0.001, *η*^*2*^_*p*_ = 0.52, and an interaction between frequency and feedback condition, *F*(3,45) = 20.94,* p* < 0.001, *η*^*2*^_*p*_ = 0.58.

To follow up on these interactions, we conducted separate analyses per frequency band. These did not reveal any main effects of session for any frequency band (theta: *F*(14,210) = 0.93, *p* = 0.46*;* mu: *F*(14,210) = 1.51, *p* = 0.20*;* beta: *F*(14,210) = 1.19, *p* = 0.32*;* high beta: *F*(14,210) = 1.22, *p* = 0.31). Moreover, main effects of feedback condition indicated reduced amplitude during feedback as compared to baseline for beta and high beta bands only (theta: *F*(1,15) = 3.29, *p* = 0.09; mu: *F*(1,15) = 0.33, *p* = 0.57*;* beta: *F*(1,15) = 16.88, *p* < 0.001; high beta: *F*(1,15) = 20.73, *p* < 0.31). Finally, the interactions between session and feedback conditions did not reach significance (theta: *F*(14,210) = 0.99, *p* = 0.44*;* mu: *F*(14,210) = 1.58, *p* = 0.17*;* beta: *F*(14,210) = 0.79, *p* = 0.56*;* high beta: *F*(14,210) = 0.69, *p* = 0.63).

Finally, we performed a subgroups comparison between those participants who showed more vs. less mu suppression prior to training. To achieve this, we decided to perform a median split (in the interest of equal group sizes based on the MSI during the first EEG session) and termed the subgroups suppressers versus non-suppressers. Note that we refrained from formal statistical analysis of subgroup differences because the study was not appropriately powered to perform such statistical comparisons due to small N of only eight per group. Nevertheless, visual inspection of Fig. 4B suggests that neurofeedback training benefits were highly parallel for suppressers and non-suppressers.

## Discussion

In the present study, we aimed at investigating the effects of mu suppression neurofeedback training on the mirror neuron system. Notably, for an evaluation of NFT effects, we included the observation not only of complex goal-directed actions, but also of social interaction scenarios, which, compared to other action categories, were typically neglected in the literature (Fox et al., 2016). Overall, whereas the present results from frequency analyses were small and inconsistent, the results from source reconstruction analysis using 64-channel EEG data provide some support for the idea that the effects of mu suppression NFT involve higher task-dependent mirror neuron system activation, particularly during the observation of complex hand movements. It may be noted that these data do not suggest that occipital alpha could have mimicked mu suppression in this condition (Hobson & Bishop, 2017, also cf. the present Fig. 3B).

Compared to other (e.g., pharmacological) interventions, neurofeedback targets specific brain circuits associated with a specific function to achieve a therapeutic effect while minimizing side effects (Weber et al., 2020). In terms of behavioral outcomes of the present study, we observed no significant changes in accuracy in the RMET test. The response time, by contrast, was substantially shorter after the neurofeedback training. As a word of caution, we cannot interpret this difference as a specific effect of NFT with any certainty, because faster responses could be a simple consequence of doing the same test repeatedly. Although this may seem unlikely when considering (1) that our subjects were mostly psychology students who frequently participate in experiments involving response speed, and (2) that there was approximately a 2-month interval between the first and the second RMET, it would have been desirable to assess an additional control outcome variable.

In contrast to studies published by the Pineda and colleagues (Friedrich et al., 2015; Pineda et al., 2008), the present frequency analysis did not exhibit significant mu suppression during complex hand movement or during social scenarios before the neurofeedback training. At the same time, source reconstruction analysis permitted to compare every condition before and after NFT, and this analysis did suggest substantial mu suppression in the complex condition in areas related to the mirror neuron system (but not in occipital areas) after NFT. Nevertheless, this finding is only partially in line with our hypotheses, as we had expected the most prominent effects of NFT during the observation of both complex and social conditions, whereas prominent differences were only seen in the complex condition. Here, significant mu desynchronization was found in bilateral pre-central and superior frontal gyrus, superior parietal lobule and precuneus on both hemispheres, and inferior parietal lobule, especially on the right side (Fig. 3-B). This could be tentatively related to functional imaging research and meta-analyses which denote a broad, bilateral system for action observation and imitation, with a parietal focus, the rostral area PFt of the inferior parietal lobule (IPL), spreading across the caudally adjacent IPL area PF and the dorsally adjacent intraparietal sulcus (IPS) areas humanIP1 and human IP2 (Svenja Caspers et al., 2012; Caspers et al., 2008) (for a review, see (Grafton & Hamilton, 2007). Interestingly, comparing pre- versus post-training assessment during the complex hand movement observation revealed mu suppression in areas such as the precuneus involved with tasks evaluating the theory of mind. While this is both beyond the known regions for the MNS and beyond the scope of discussion for the present pilot report, it may be noted that the precuneus has been associated with other social cognitive functions (Molenberghs et al., 2016; Schurz et al., 2014).

In source reconstruction analysis, several clusters showed mu rhythm synchronization (enhancement) while observing the simple hand movements and social scenarios. During the observation of simple hand movements, two significant clusters of right occipital alpha enhancement appeared after NFT. During the observation of social scenarios, the left frontal operculum pars orbitalis, exhibited mu enhancement. This region has been associated with top-down control of social and emotional processing (Adolphs, 2003; Britton et al., 2006). However, in view of the small sample and the small clusters that were associated with mu enhancement in the present study, we think that replication is necessary before interpreting these findings.

The benefit of NFT consists of creating task-dependent neural plasticity (Sitaram et al., 2017; Weber et al., 2020). The average neurofeedback intervention time (including protocols that target affective systems or the MNS) is approximately 20–30 sessions (R. Coben, 2008; M. E. J. Kouijzer et al., 2009a, 2009b; Pineda et al., 2014; Thompson et al., 2010). It remains a distinct possibility that the present NFT protocol with only 15 sessions could become more efficient by extending the number of training sessions.

The present pilot study had several limitations. First, the resources available for the present project unfortunately did not permit to test more participants and to apply more training sessions, which could seriously limit the statistical power of the present study. Second, relative to recommendations for full clinical or replication studies (cf. Ros et al., 2020), it should be noted that the present exploratory study did not use a control group or a control intervention (such as a training program that does not involve neurofeedback, or one that involves sham-neurofeedback). Third, and as discussed above, the assessment of multiple outcome variables apart from the RMET would have been desirable. Fourth, a notorious issue in mu suppression EEG studies is whether changes in the 8–13 Hz band are indeed related to activity in sensorimotor regions, as opposed particularly with occipital regions, which are involved in generating the classical alpha (8–13 Hz) rhythm. Although we performed NFT training on only a few electrodes over sensorimotor areas, we tried to address this issue by evaluating training effects with 64-channel EEG and source reconstruction methods. Fifth, while the present neurofeedback protocol was relatively novel in the sense that it engaged suppression training only, the multiple frequency bands that were part of the preset feedback protocol – although inspired by previous research – could be a subject of further consideration. Finally, another limitation of the present study could relate to the specific selection of stimulus videos to elicit mu suppression. For instance, the present hand movement videos only involved observations of right-hand movements, which might be related to hemispheric asymmetries in observed mu activity.

In conclusion, while the present study clearly requires replication and extension, the present results are broadly consistent with the idea that mu rhythm suppression can be a marker for the function of the human mirror neuron system. Moreover, our results provide preliminary evidence that a relatively short EEG neurofeedback training incentivizing mu suppression in particular can be used to train and modify this system. If confirmed in larger studies with more powerful designs, the present findings could also have possible implications for interventions in specific target groups, such as individuals on the autism spectrum.

### Supplementary Information

Below is the link to the electronic supplementary material.

Supplementary file1 (PDF 12174 KB)

## Data Availability

Extensive further supplemental information (figures, tables, scripts, raw data) can be found in the associated OSF Repository: https://osf.io/n5wrk/?view_only=873a011e9b65461eaafdc9370817a9e9

## Footnotes

1 Note that we included theta and beta rhythms as they can be associated with distraction (Kilavik, Zaepffel, Brovelli, MacKay, & Riehle, 2013; Pineda et al., 2014) We wanted to provide a negative feedback for voluntary movements to reduce the chance of our participants trying to reduce their mu rhythm via action execution. At the same time we did not want to add another electrode for electromyography and make neurofeedback uncomfortable. According to corticomuscular coherence studies (Kilavik et al., 2013; Ritterband-Rosenbaum et al., 2017), a transient but prominent increase in high beta power occurs after the end of any action execution, a well-known phenomenon called post-movement beta 'rebound'. The magnitude of the beta rebound seems to parallel the speed of the preceding movement. We had observed inflated high beta power as a result of bodily movement of participants and we decided to use this phenomen to reduce mu suppression due to action execution.
