# Supplementary material for: Mu-Suppression Neurofeedback Training Targeting the Mirror Neuron System: A Pilot Study
Source: Appl Psychophysiol Biofeedback. 2024 May 13;49(3):457–71. doi: 10.1007/s10484-024-09643-4 (PMC11310260; doi:10.1007/s10484-024-09643-4)
Supplement: Supplementary file 1 — Supplementary file1 (PDF 12174 KB) [file 10484_2024_9643_MOESM1_ESM.pdf]

Mu-Suppression Neurofeedback Training Targeting the Mirror Neuron System: A Pilot Study

Neurofeedback training (NFT) is a promising adjuvant intervention method. The desynchronization of mu rhythm (8-13 Hz) in the electroencephalogram (EEG) over centroparietal areas is known as a valid indicator of mirror neuron system (MNS) activation, which has been associated with social skills. Still, the effect of neurofeedback training on the MNS requires to be well investigated. The present study examined the possible impact of NFT with a mu suppression training protocol encompassing 15 NFT training sessions (45 min each) on 16 healthy neurotypical participants. In separate pre- and post-training sessions, 64-channel EEG was recorded while participants (1) observed videos with various types of movements (including complex goal-directed hand movements and social interaction scenes) and (2) performed the "Reading the Mind in the Eyes Test" (RMET). EEG source reconstruction analysis revealed statistically significant mu suppression during hand movement observation across MNS-attributed fronto-parietal areas after NFT. Despite the fact that numerical mu suppression appeared to be visible in a majority of participants during goal-directed hand movement observation, the frequency analysis showed no significant mu suppression after NFT. At the behavioral level, RMET accuracy scores did not suggest an effect of NFT on the ability to interpret subtle emotional expressions, although RMET response times were reduced after NFT. In conclusion, the present study exhibited preliminary and partial evidence that Mu suppression NFT can induce mu suppression in MNS-attributed areas. More powerful experimental designs and longer trainings may be necessary to induce substantial and consistent mu suppression, particularly while observing social scenarios.

Here you will find all the statistical analysis (except the cluster permutation t-tests of the scource reconstruction that are performed in brainstorm) regarding this study.

```
#--------------Libraries ----------------
# version
sessionInfo()
```

```
## R version 4.2.3 (2023-03-15 ucrt)
## Platform: x86_64-w64-mingw32/x64 (64-bit)
## Running under: Windows 10 x64 (build 22621)
##
## Matrix products: default
##
## locale:
## [1] LC_COLLATE=German_Germany.utf8  LC_CTYPE=German_Germany.utf8
## [3] LC_MONETARY=German_Germany.utf8 LC_NUMERIC=C
## [5] LC_TIME=German_Germany.utf8
##
## attached base packages:
## [1] stats     graphics  grDevices utils     datasets  methods   base
##
## loaded via a namespace (and not attached):
##  [1] digest_0.6.31   R6_2.5.1        jsonlite_1.8.4  evaluate_0.21
##  [5] cachem_1.0.8    rlang_1.1.0     cli_3.6.0       rstudioapi_0.14
##  [9] jquerylib_0.1.4 bslib_0.4.2     rmarkdown_2.21  tools_4.2.3
## [13] xfun_0.37       yaml_2.3.7      fastmap_1.1.1   compiler_4.2.3
## [17] htmltools_0.5.4 knitr_1.42      sass_0.4.6
```

```
version
```

```
##                _
## platform       x86_64-w64-mingw32
## arch           x86_64
## os             mingw32
## crt            ucrt
## system         x86_64, mingw32
## status
## major          4
## minor          2.3
## year           2023
## month          03
## day            15
## svn rev        83980
## language       R
## version.string R version 4.2.3 (2023-03-15 ucrt)
## nickname       Shortstop Beagle
```

```
# setting time
Sys.setenv(TZ="Europe/Berlin", location=FALSE)
as.POSIXct(Sys.time())
```

```
## [1] "2023-06-25 17:00:20 CEST"
```

```
# libraries
library(broom)
library(ggsignif)
library(apaTables)
library(pander)
library(readxl)
library(dplyr)
library(ggplot2)
library(ez)
#library(boot) # make demo table
library(table1) # make demo table
#library(arsenal) # make demo table
#library(knitr)    #  For knitting document and include_graphics function
library(lme4)
#library(sjPlot)
#library(broom)
#library(reshape2)
#library(corrplot)
#library(forestplot)
library(lmerTest)
#library(sjPlot)
library(tidyverse)
#library(EMAtools)
library("dplyr")
library("ggplot2")
library("tidyr")
library("Hmisc")
library("ez")
library("schoRsch")
library("gmodels") # The CrossTable()-function from the "gmodels"-package offers an easy way to create a detailed
library("psych") # Sometimes you just want to get a quick overview over your data, which shows the most common statistica
l characteristics of your data
library("foreign")
# this package allows you to read and save different file formats
library("data.table")
# this package has a function to easily rename columns called setnames(dataframe, "old name of column", "new name of a co
lumn")
library("rstatix")
library("nlme")
library("reshape")
library("afex") # easy for ANOVAs and post hocs, which has aov_ez() function-> https://www.psychologie.uni-heidelberg.de/
ae/meth/team/mertens/blog/anova_in_r_made_easy.nb.html
library("emmeans")
library("schoRsch")
library("base")
library("lsmeans")
library("formattable")# makes nice amazing tables for raw data!
library(Rcmdr)# chapter5, assumptions
library(car)# chapter5, assumptions
library(pastecs)# chapter5, assumptions
library(effectsize) #calculates effect sizes
library("rex") #write excel files
library("covr") #write excel files
library("openxlsx") #write excel files
library(effectsize)
windowsFonts("Arial" = windowsFont("Arial"))
```

Demographic data+ AQ

All participants completed the autism-spectrum quotient questionnaire (Freitag et al., 2007) to exclude participants with an autism diagnosis. None of the participants were excluded due to high autistic traits (*M* = 13.75, *SD*= 4.6).

```
#---------------Demographic data & AQ ----------------

DD<-read.csv(file=("C:/Users/dastg/Desktop/Main-Projects/Empra_2019_Dastgheib/Statistical_Analysis/Input/15VP-NFT/AQ.cs
v"),header = TRUE)

desDD<-describe(DD)

formattable(desDD)
```

	vars	n	mean	sd	median	trimmed	mad	min	max	range	skew	kurtosis	se
Verantwortlich*	1	16	6.812500	2.9261750	8.5	7.000000	1.4826	1	10	9	-0.7100484	-1.1003556	0.7315437
ID*	2	16	8.500000	4.7609523	8.5	8.500000	5.9304	1	16	15	0.0000000	-1.4262408	1.1902381
VP	3	16	15.625000	13.7446959	8.5	15.285714	8.8956	1	35	34	0.4051644	-1.8204062	3.4361740
Geschlecht*	4	16	1.812500	0.4031129	2.0	1.857143	0.0000	1	2	1	-1.4535320	0.1325120	0.1007782
Alter	5	16	22.187500	3.0379544	21.5	21.785714	2.2239	19	31	12	1.3843448	1.7401086	0.7594886
Gesamtscore	6	16	13.750000	4.5680047	14.0	13.571429	2.9652	5	25	20	0.4937367	0.5623205	1.1420012
Social_skills	7	13	1.692308	1.0315535	1.0	1.727273	1.4826	0	3	3	0.1517665	-1.5533098	0.2861015
Attention_switching	8	13	3.461538	1.4500221	3.0	3.363636	1.4826	2	6	4	0.2973962	-1.5835777	0.4021638
Attention_to_detail	9	13	4.076923	2.4651416	4.0	4.000000	2.9652	0	9	9	0.1343579	-0.8454295	0.6837073
Communication	10	13	1.692308	1.4935760	2.0	1.636364	1.4826	0	4	4	0.2098356	-1.5181631	0.4142434
Imagination	11	13	2.307692	1.9315199	2.0	2.181818	1.4826	0	6	6	0.3661009	-1.1306630	0.5357072

Behavioral data (RMET) analysis before and after NFT

```
#----------------RMTE analysis before and after-----------------
#Loading data:

file_path <- "C:/Users/dastg/Desktop/Main-Projects/Empra_2019_Dastgheib/Statistical_Analysis/Input/15VP-NFT/RMET_before_a
nd_after.csv"

# Try different encodings until you find the one that works
encodings <- c("UTF-8", "latin1")

for (encoding in encodings) {
  # Attempt to read the file with the current encoding
  RMETraw <- tryCatch(
    read.delim(file_path, header = TRUE, sep = "\t", skipNul = TRUE, encoding = encoding),
    error = function(e) NULL
  )

  # Check if the read was successful
  if (!is.null(RMETraw))
    break
}

if (is.null(RMETraw)) {
  # Failed to read the file with any encoding
  print("Unable to read the file. Please check the encoding.")
} else {
  # File was successfully read
  print("File read successfully.")
}
```

```
## [1] "File read successfully."
```

```
#Selecting the relevant cloumns

RMET <- RMETraw %>% select( # for columns
                    Subject,
                    Session,
                    Age,
                    Sex,
                    Correct_Spelled,
                    Images,
                    Slide2.ACC,
                    Slide2.CRESP,
                    Slide2.RESP,
                    Slide2.RT)
setnames( RMET, "Correct_Spelled", "Emotion")
setnames( RMET, "Slide2.ACC", "Accuracy")
setnames( RMET, "Slide2.RT", "ResponseTime")
setnames( RMET, "Slide2.RESP", "Answer")
setnames( RMET, "Slide2.CRESP", "Correct Answer")

#Removing practice trial
RMET <- RMET %>% filter(Emotion != "panisch")

#Descriptive statistics
desRMET<-RMET %>% group_by (Session) %>%summarise_at(vars(Accuracy),funs(min,median,mean, max, sd))

attach(desRMET)
confidence_level <-0.95 # a is considered <0.05 before starting the research => thus, a/2 should be 0.05/2 =0.025
n<-16
z<-qt((1+confidence_level)/2, df= n-1 )

desRMET<-mutate(desRMET, Accuracy_se= sd/sqrt(16))
desRMET<-mutate(desRMET, Accuracy_CI= z*Accuracy_se)

write.csv(RMET,"C:/Users/dastg/Desktop/Main-Projects/Empra_2019_Dastgheib/Statistical_Analysis/Input/15VP-NFT/desRMETAcc.
csv", row.names = FALSE)

#Testing accuracy
A <- RMET %>% group_by(Subject, Session) %>% summarise(Mean = mean(Accuracy),
                                                       SD = sd(Accuracy),
                                                       N = length(Accuracy),
                                                       SE = SD/sqrt(N),
                                                       CI = SE*2.13)

test1 <- t.test(Mean ~ Session, data = A , paired = TRUE, na.rm = TRUE)

test1
```

```
##
##  Paired t-test
##
## data:  Mean by Session
## t = -0.33294, df = 15, p-value = 0.7438
## alternative hypothesis: true mean difference is not equal to 0
## 95 percent confidence interval:
##  -0.06425280  0.04689169
## sample estimates:
## mean difference
##    -0.008680556
```

```
#use lsr package:
library(lsr)
cohensD(Mean ~ Session,
       data   = A,
       method = "paired")
```

```
## [1] 0.08323474
```

```
#Here is 0.08323474

capture.output(test1, file="C:/Users/dastg/Desktop/Main-Projects/Empra_2019_Dastgheib/Statistical_Analysis/Input/15VP-NF
T/Output/RMET-ACC.txt")

#Testing Response time
#Getting the Rsponse tim for the correct responses:
Correct<-subset(RMET,RMET$Accuracy== 1)
RMET <-Correct

#Descriptive statistics
desRMET<-RMET %>% group_by (Session) %>%summarise_at(vars(ResponseTime),funs(min,median,mean, max, sd))

attach(desRMET)
confidence_level <-0.95 # a is considered <0.05 before starting the research => thus, a/2 should be 0.05/2 =0.025
n<-16
z<-qt((1+confidence_level)/2, df= n-1 )

desRMET<-mutate(desRMET, ResponseTime_se= sd/sqrt(16))
desRMET<-mutate(desRMET, ResponseTime_CI= z*ResponseTime_se)

write.csv(RMET,"C:/Users/dastg/Desktop/Main-Projects/Empra_2019_Dastgheib/Statistical_Analysis/Input/15VP-NFT/desRMETRT.c
sv", row.names = FALSE)

B <-RMET %>% group_by(Subject, Session) %>% summarise(Mean = mean(ResponseTime),
                                                           SD = sd(ResponseTime),
                                                           N = length(ResponseTime),
                                                           SE = SD/sqrt(N),
                                                           CI = SE*2.13)
test2 <- t.test(Mean ~ Session, data =B , paired = TRUE, na.rm = TRUE)

test2
```

```
##
##  Paired t-test
##
## data:  Mean by Session
## t = 3.8908, df = 15, p-value = 0.001448
## alternative hypothesis: true mean difference is not equal to 0
## 95 percent confidence interval:
##   480.2507 1643.9194
## sample estimates:
## mean difference
##        1062.085
```

```
#use lsr package:
cohensD(Mean ~ Session,
       data   = B,
       method = "paired")
```

```
## [1] 0.9726912
```

```
#Here is 0.9726912

capture.output(test2, file="C:/Users/dastg/Desktop/Main-Projects/Empra_2019_Dastgheib/Statistical_Analysis/Input/15VP-NF
T/Output/RMET-ResTime.txt")
```

Tidying up the database

The EEG values in the analysis from now on are all indexes, which means: <u>the power of a target scenario over the power of the ball movements scenario (baseline) in a specific frequency band</u>**.** Regarding the mu frequency band, the index is known as the **mu suppression index** or **MSI.**

EEG analysis is performed in the Brainstorm Matlab toolbox, and the values are exported to R as a CSV file!

```
#--------------- Tidying up the database ----------------

#EEGall<-read.csv(file=("C:\\Users\\dastg\\Desktop\\Main-Projects\\Empra_2019_Dastgheib\\Statistical_Analysis\\Raw-Data_17032021\\RawEEG-MSI-NFT-16vp.csv"),header = TRUE)

#setwd("C:\\Users\\dastg\\Desktop\\Main-Projects\\Empra_2019_Dastgheib\\Statistical_Analysis\\Raw-Data_17032021\\EEG-individual-databases")

#BA<-read.csv( file=("MSIsocial35after.csv"),header = TRUE);BA$condition<- "social" ;BA$session <-"after NFT" ; BA$X <- NULL ;BA$index <- "MSI" ;BA$VP<-"35" ;setnames(BA, "Freq", "channel");BA[ ,c('highmu', 'lowmu')] <- list(NULL)

#EEGall1 <-rbind(BA,EEGall1)
#rm(BA,EEGall)


#save

#write.csv(EEGall1,"C:\\Users\\dastg\\Desktop\\Main-Projects\\Empra_2019_Dastgheib\\Statistical_Analysis\\Raw-Data_17032021\\RawEEG-MSI-NFT-16vp.csv", row.names = FALSE)

#**Remove useless channels**

EEGall<-read.csv(file=("C:\\Users\\dastg\\Desktop\\Main-Projects\\Empra_2019_Dastgheib\\Statistical_Analysis\\Raw-Data_17032021\\RawEEG-MSI-NFT-16vp.csv"),header = TRUE)

attach(EEGall) #=>This function allows me to skip indexing and $sign next time...

EEG <- EEGall %>% filter(channel=="C3" | channel== "C4" | channel=="Cz" )

rm(EEGall)

#order and sort database

#display factor levels for condition
EEG$condition <- as.factor(EEG$condition)
levels(EEG$condition)
```

```
## [1] "complex" "simple"  "social"
```

```
#re-order factor levels for condition
EEG$condition <- factor(EEG$condition, levels=c("simple", "complex", "social"))
#display factor levels for session
EEG$session <- as.factor(EEG$session)
levels(EEG$session)
```

```
## [1] "after NFT"  "before NFT"
```

```
#re-order factor levels for session
EEG$session <- factor(EEG$session, levels=c("before NFT", "after NFT"))
#display factor levels for channels
EEG$channel <- as.factor(EEG$channel)
levels(EEG$channel)
```

```
## [1] "C3" "C4" "Cz"
```

```
#re-order factor levels for channels

EEG$channel <- factor(EEG$channel, levels=c("C3", "Cz", "C4"))

write.csv(EEG,"C:/Users/dastg/Desktop/Main-Projects/Empra_2019_Dastgheib/Statistical_Analysis/Input/15VP-NFT/EEG-NFT-onlyC3C4Cz.csv", row.names = FALSE)
```

check the freq-distribution of data with tables and histogram

```
#--------------- check the distribution of data ----------------

EEG<-read.csv(file=("C:/Users/dastg/Desktop/Main-Projects/Empra_2019_Dastgheib/Statistical_Analysis/Input/15VP-NFT/EEG-NF
T-onlyC3C4Cz.csv"),header = TRUE)

my_colors <- c("#fec44f", "#4F5B66", "#00B2B2")

#Mu Frequency

mu_scatter<- ggplot( EEG, aes( x= session , y= mu, color= session))
mu_scatter+geom_hline(yintercept = 0, colour ="#979797") + geom_point(position = position_jitterdodge(), alpha=0.6, size=
1.5)+facet_grid( channel~condition) + labs(title= "MSIs for mu frequency, 8-12 Hz", x = "Group", y = "Mu Suppression Inde
x (MSI)", colour = "Group")+ scale_color_manual(values = my_colors)+scale_y_continuous( limits = c(-1,1))+ theme_bw()+ th
eme(text = element_text(size = 12, family = "Arial"))+ facet_grid( channel~condition)
```

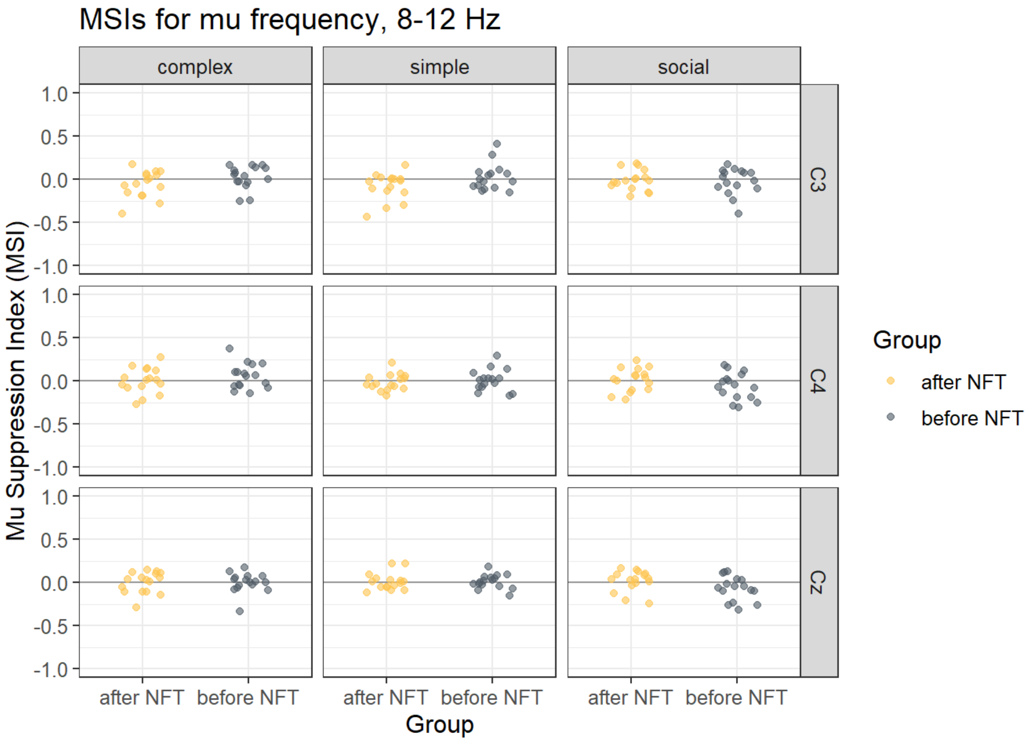


```
#Histogram plot----------------------------------
#per session, per condition, per channel
EEG_before <-subset(EEG,session=="before NFT")
EEG_before_his <- ggplot(EEG_before, aes(mu))
EEG_before_his + geom_histogram(aes(y=..density..), colour="black", fill= "#F0F0F0")+ geom_density(alpha=.2, fill="#fec44
f")+facet_wrap(~session)+facet_grid( channel~condition)+ theme_bw()+ labs( x = "Mu Suppression Index (MSI)", y = "Count")
+ theme(text = element_text(size = 15, family = "Arial"))+ theme(panel.spacing = unit(0.8, "lines"))
```

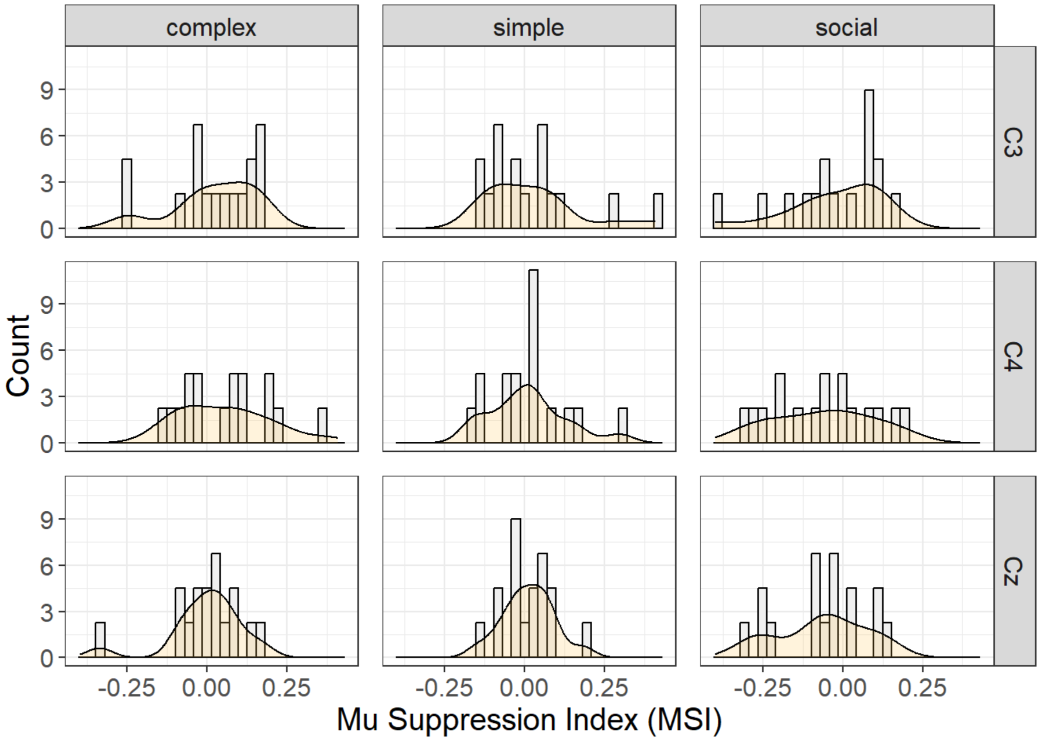


```
#
EEG_after <-subset(EEG,session=="after NFT")
EEG_after_his <- ggplot(EEG_after, aes(mu))
EEG_after_his + geom_histogram(aes(y=..density..), colour="black", fill= "#F0F0F0")+ geom_density(alpha=.2, fill="#fec44
f")+facet_wrap(~session)+facet_grid( channel~condition)+ theme_bw()+ labs( x = "Mu Suppression Index (MSI)", y = "Count")
+ theme(text = element_text(size = 15, family = "Arial"))+ theme(panel.spacing = unit(0.8, "lines"))
```

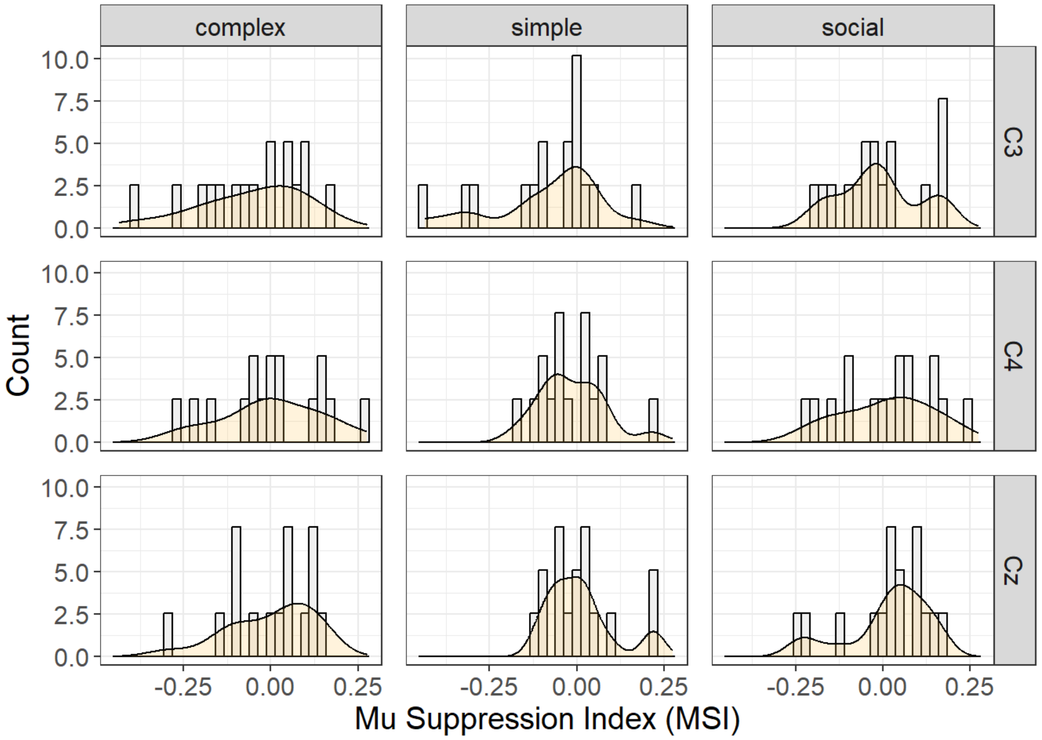

Check the normality of the data

```
#--------------- Check the normality of the data ----------------

EEG<-read.csv(file=("C:/Users/dastg/Desktop/Main-Projects/Empra_2019_Dastgheib/Statistical_Analysis/Input/15VP-NFT/EEG-NF
T-onlyC3C4Cz.csv"),header = TRUE)
EEG_before <-subset(EEG,session=="before NFT")

simple_before<-subset(EEG_before,EEG_before$condition== "simple")
stat.desc(simple_before$mu, basic = FALSE, norm = TRUE)
```

```
##       median         mean      SE.mean CI.mean.0.95          var      std.dev
##  0.010496269  0.014938016  0.017258510  0.034719644  0.014297096  0.119570466
##     coef.var     skewness     skew.2SE     kurtosis     kurt.2SE   normtest.W
##  8.004441136  0.975171153  1.420913764  1.344167981  0.996569631  0.933641530
##   normtest.p
##  0.009310708
```

```
simple_before<-subset(EEG_before,EEG_before$condition== "complex")
stat.desc(simple_before$mu, basic = FALSE, norm = TRUE)
```

```
##       median         mean      SE.mean CI.mean.0.95          var      std.dev
##   0.03534465   0.02599901   0.01871041   0.03764049   0.01680381   0.12962953
##     coef.var     skewness     skew.2SE     kurtosis     kurt.2SE   normtest.W
##   4.98594101  -0.24756636  -0.36072687   0.69091516   0.51224629   0.97763104
##   normtest.p
##   0.48420818
```

```
social_before<-subset(EEG_before,EEG_before$condition== "social")
stat.desc(simple_before$mu, basic = FALSE, norm = TRUE)
```

```
##       median         mean      SE.mean CI.mean.0.95          var      std.dev
##   0.03534465   0.02599901   0.01871041   0.03764049   0.01680381   0.12962953
##     coef.var     skewness     skew.2SE     kurtosis     kurt.2SE   normtest.W
##   4.98594101  -0.24756636  -0.36072687   0.69091516   0.51224629   0.97763104
##   normtest.p
##   0.48420818
```

```
EEG_after <-subset(EEG,session=="after NFT")

simple_after<-subset(EEG_after,EEG_after$condition== "simple")
stat.desc(simple_after$mu, basic = FALSE, norm = TRUE)
```

```
##       median         mean      SE.mean CI.mean.0.95          var      std.dev
## -0.021295908 -0.028970698  0.017936470  0.036083524  0.015442414  0.124267510
##     coef.var     skewness     skew.2SE     kurtosis     kurt.2SE   normtest.W
## -4.289420653 -0.697979598 -1.017020258  1.819922793  1.349295483  0.926862523
##   normtest.p
##  0.005234142
```

```
simple_after<-subset(EEG_after,EEG_after$condition== "complex")
stat.desc(simple_after$mu, basic = FALSE, norm = TRUE)
```

```
##       median         mean      SE.mean CI.mean.0.95          var      std.dev
##  0.009061889 -0.017150889  0.020381789  0.041002870  0.019940031  0.141209175
##     coef.var     skewness     skew.2SE     kurtosis     kurt.2SE   normtest.W
## -8.233344391 -0.508088680 -0.740331783 -0.209441793 -0.155280689  0.973112310
##   normtest.p
##  0.333219211
```

```
social_after<-subset(EEG_after,EEG_after$condition== "social")
stat.desc(simple_after$mu, basic = FALSE, norm = TRUE)
```

```
##       median         mean      SE.mean CI.mean.0.95          var      std.dev
##  0.009061889 -0.017150889  0.020381789  0.041002870  0.019940031  0.141209175
##     coef.var     skewness     skew.2SE     kurtosis     kurt.2SE   normtest.W
## -8.233344391 -0.508088680 -0.740331783 -0.209441793 -0.155280689  0.973112310
##   normtest.p
##  0.333219211
```

```
#Levene's test
leveneTest(data=EEG, EEG$mu, EEG$session)
```

	Df <int>	F value <dbl>	Pr(>F) <dbl>
group	1	0.2873003	0.5923724
	286	NA	NA

2 rows

```
leveneTest(data=EEG, EEG$mu, EEG$condition)
```

	Df <int>	F value <dbl>	Pr(>F) <dbl>
group	2	1.851475	0.1588888
	285	NA	NA

2 rows

```
leveneTest(data=EEG, EEG$mu, EEG$channel)
```

	Df <int>	F value <dbl>	Pr(>F) <dbl>
group	2	1.984253	0.1393781
	285	NA	NA

2 rows

Descriptive statistics

```
#--------------- descriptive statistics----------------


desEEG<-EEG %>% group_by (session,condition, channel) %>%
  summarise_at(vars(mu, delta, theta, beta, gamma1, gamma2), funs(min,median, mean, max, sd))

desEEG2<-EEG %>% group_by (condition,session) %>%
  summarise_at(vars(mu, delta, theta, beta, gamma1, gamma2), funs(min,median, mean, max, sd))

desEEG3<-EEG %>% group_by (session) %>%
  summarise_at(vars(mu, delta, theta, beta, gamma1, gamma2), funs(min,median, mean, max, sd))

write.csv(desEEG,"C:/Users/dastg/Desktop/Main-Projects/Empra_2019_Dastgheib/Statistical_Analysis/Input/15VP-NFT/desEEG-16
VP-overConditionSessionChannel.CSV", row.names = FALSE)

write.csv(desEEG2,"C:/Users/dastg/Desktop/Main-Projects/Empra_2019_Dastgheib/Statistical_Analysis/Input/15VP-NFT/desEEG-1
6VP-overConditionSession.CSV", row.names = FALSE)

write.csv(desEEG3,"C:/Users/dastg/Desktop/Main-Projects/Empra_2019_Dastgheib/Statistical_Analysis/Input/15VP-NFT/desEEG-1
6VP-overSession.CSV", row.names = FALSE)

#--------------- descriptive statistics (calculating CIs)----------------

attach(desEEG)

confidence_level <-0.95 # a is considered <0.05 before starting the research => thus, a/2 should be 0.05/2 =0.025

n<-16

z<-qt((1+confidence_level)/2, df= n-1 )

#mu_se <- desEEG$mu_sd/sqrt(n)
#mu_CI <-z*mu_se

#mu
desEEG<-mutate(desEEG, mu_se=mu_sd/sqrt(10))

desEEG<-mutate(desEEG, mu_CI= z*mu_se)

#delta
desEEG<-mutate(desEEG, delta_se = delta_sd/sqrt(10))

desEEG<-mutate(desEEG, delta_CI= z*delta_se)

#theta
desEEG<-mutate(desEEG, theta_se = theta_sd/sqrt(10))

desEEG<-mutate(desEEG, theta_CI= z*theta_se)

#beta
desEEG<-mutate(desEEG, beta_se = beta_sd/sqrt(10))

desEEG<-mutate(desEEG, beta_CI= z*beta_se)

#gamma1
desEEG<-mutate(desEEG, gamma1_se = gamma1_sd/sqrt(10))

desEEG<-mutate(desEEG, gamma1_CI= z*gamma1_se)

#gamma2
desEEG<-mutate(desEEG, gamma2_se = gamma2_sd/sqrt(10))

desEEG<-mutate(desEEG, gamma2_CI= z*gamma2_se)

desEEG_Spare <- desEEG

write.csv(desEEG,"C:/Users/dastg/Desktop/Main-Projects/Empra_2019_Dastgheib/Statistical_Analysis/Input/15VP-NFT/desEEG-16
VP-overConditionSessionChannel.CSV", row.names = FALSE)
```

```
#**********************************************************
rm(desEEG2,desEEG3,desEEG_Spare)
```

ANOVAS and Post hocs

ANOVA for mu

```
#--------------- ANOVA and Effect sizes (Mu) ----------------
#ANOVA using afex and without need to aggregate:

#load the data set:

EEG<-read.csv(file=("C:/Users/dastg/Desktop/Main-Projects/Empra_2019_Dastgheib/Statistical_Analysis/Input/15VP-NFT/EEG-NFT-onlyC3C4Cz.csv"),header = TRUE)

#As you see, contrasts are automatically set to effect-coding (contr.sum) and, since we have more than one observation per cell, the data were automatically aggregated.

#The output is similar to the usual analysis of variance table with three differences. We have two additional columns showing the mean square error (MSE) and generalized 2 (ges) as an effect size measure. Also, p-values are displayed nicer.

#If we don’t use the return argument, the object storing the result is a list with 6 elements. This is fine for small datasets where speed does not matter, but the more data you have, the more expensive it becomes to not specify a return argument. These elements are explained in numbers below when you operate the following code:


mu_all <- aov_ez("VP","mu", EEG,within=c("condition", "channel","session"))

names(mu_all)
```

```
## [1] "anova_table" "aov"         "Anova"       "lm"          "data"
```

```
summary(mu_all)
```

```
##
## Univariate Type III Repeated-Measures ANOVA Assuming Sphericity
##
##                            Sum Sq num Df Error SS den Df F value   Pr(>F)
## (Intercept)              0.019668      1  1.23260     15  0.2393 0.631758
## condition                0.034246      2  0.84317     30  0.6092 0.550356
## channel                  0.022536      2  0.56936     30  0.5937 0.558631
## session                  0.007388      1  0.83017     15  0.1335 0.719947
## condition:channel        0.039175      4  0.25084     60  2.3426 0.064984 .
## condition:session        0.160646      2  0.32549     30  7.4033 0.002436 **
## channel:session          0.091369      2  0.39825     30  3.4413 0.045131 *
## condition:channel:session 0.008770      4  0.22106     60  0.5951 0.667520
## ---
## Signif. codes:  0 '***' 0.001 '**' 0.01 '*' 0.05 '.' 0.1 ' ' 1
##
##
## Mauchly Tests for Sphericity
##
##                           Test statistic p-value
## condition                        0.92573 0.58261
## channel                          0.94766 0.68640
## condition:channel                0.36911 0.14981
## condition:session                0.91292 0.52849
## channel:session                  0.99449 0.96203
## condition:channel:session        0.33247 0.10005
##
##
## Greenhouse-Geisser and Huynh-Feldt Corrections
##  for Departure from Sphericity
##
##                           GG eps Pr(>F[GG])
## condition                0.93086   0.539527
## channel                  0.95027   0.550717
## condition:channel        0.62299   0.098863 .
## condition:session        0.91990   0.003276 **
## channel:session          0.99452   0.045438 *
## condition:channel:session 0.70832   0.612406
## ---
## Signif. codes:  0 '***' 0.001 '**' 0.01 '*' 0.05 '.' 0.1 ' ' 1
##
##                             HF eps  Pr(>F[HF])
## condition                1.0575032 0.550356426
## channel                  1.0843370 0.558630976
## condition:channel        0.7569440 0.085092385
## condition:session        1.0424133 0.002436026
## channel:session          1.1461289 0.045131444
## condition:channel:session 0.8903856 0.648843675
```

```
#As you see, the output shows the results for a RM-ANOVA assuming sphericity. In addition, Mauchly Test for Sphercity as
well as Greenhouse Geisser and Huynh-Feldt corrected p-values were computed for the respective effects.

capture.output(mu_all, file = "C:/Users/dastg/Desktop/Main-Projects/Empra_2019_Dastgheib/Statistical_Analysis/Input/15VP-
NFT/Output/Tests/NFT-ANOVA-Mu.txt")


#---------------Effect sizes for ANOVA ----------------

#calculate the effect sizes Condition*Session interaction:

library(effectsize)
#General form of the function:
#F_to_eta2(f, df, df_error, ci = 0.95, alternative = "greater", ...)

F_to_eta2(
  f = c(0.6092,0.5937,0.1335,2.3426,7.4033,3.4413,0.5951),
  df = c(2,2,1,4,2,2,4),
  df_error = c(30,30,15,60,30,30,60)
)
```

Eta2_partial <dbl>	CI <dbl>	CI_low <dbl>	CI_high <dbl>
0.039028265	0.95	0.000000000	1
0.038073068	0.95	0.000000000	1
0.008821489	0.95	0.000000000	1
0.135077785	0.95	0.000000000	1
0.330455781	0.95	0.095526092	1
0.186608319	0.95	0.002538101	1
0.038159422	0.95	0.000000000	1

7 rows

```
#Eta2 (partial) |       95% CI

#0.04           | [0.00, 1.00]
#0.04           | [0.00, 1.00]
#8.82e-03       | [0.00, 1.00]
#0.14           | [0.00, 1.00]
#0.33           | [0.10, 1.00]
#0.19           | [0.00, 1.00]
#0.04           | [0.00, 1.00]
Effectsize<-F_to_eta2(
  f = c(0.6092,0.5937,0.1335,2.3426,7.4033,3.4413,0.5951),
  df = c(2,2,1,4,2,2,4),
  df_error = c(30,30,15,60,30,30,60)
)
capture.output(Effectsize, file = "C:/Users/dastg/Desktop/Main-Projects/Empra_2019_Dastgheib/Statistical_Analysis/Input/1
5VP-NFT/Output/Tests/effectsizes.txt")

#Plot the interaction session*condition -----------
#Gray
EEG$session <- factor(EEG$session, levels=c("before NFT", "after NFT"))
EEG$channel <- factor(EEG$channel, levels=c("C3", "Cz", "C4"))
afex_plot(mu_all, x = "session", trace = "condition",error = "between", mapping = c("shape"))+geom_hline(yintercept = 0,
colour ="#979797", linewidth=0.2)+labs( x = "Session", y = "Mu Suppression Index (MSI)")+ scale_color_manual(values = my_
colors)+ theme_bw()+ theme(text = element_text(size = 12, family = "Arial"))+scale_y_continuous( limits = c(-0.5,0.5), br
eaks= c(-0.4,0.0,0.4))
```

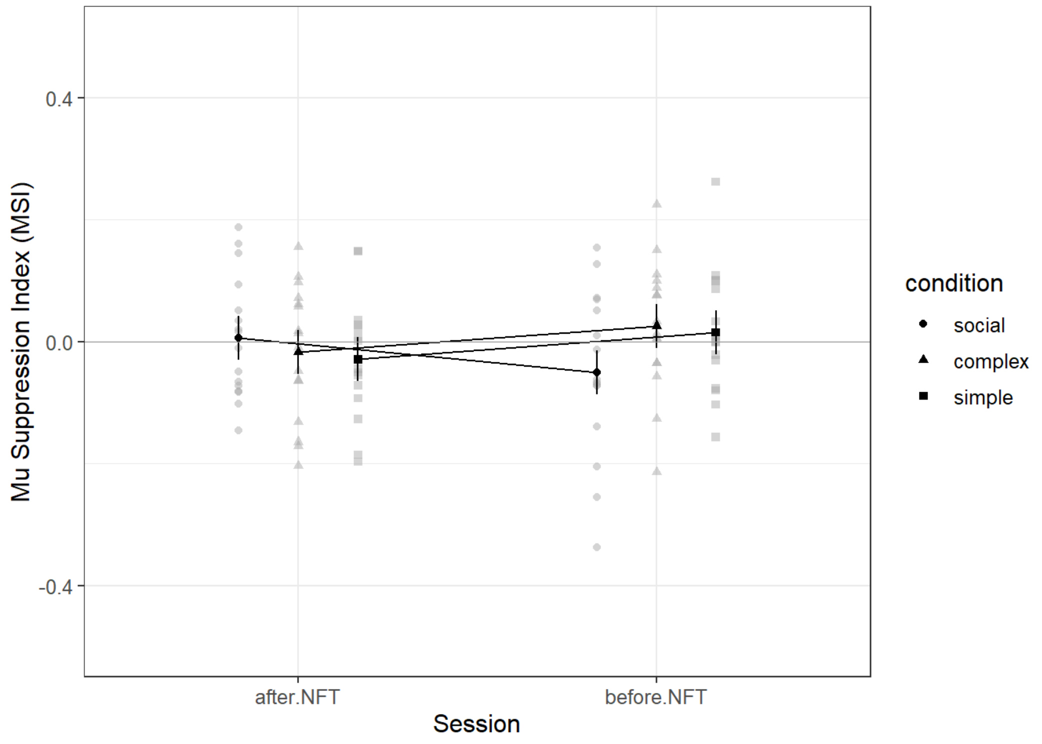

```
#Colored
afex_plot(mu_all, x = "session", trace = "condition",error = "within", mapping = c("shape","colour"))+geom_hline(yintercept = 0, colour ="#979797", linewidth=0.2)+labs( x = "Session", y = "Mu Suppression Index (MSI)")+ scale_color_manual(values = my_colors)+ theme_bw()+ theme(text = element_text(size = 12, family = "Arial"))+scale_y_continuous( limits = c(-0.5, 0.5), breaks= c(-0.4,0.0,0.4))
```

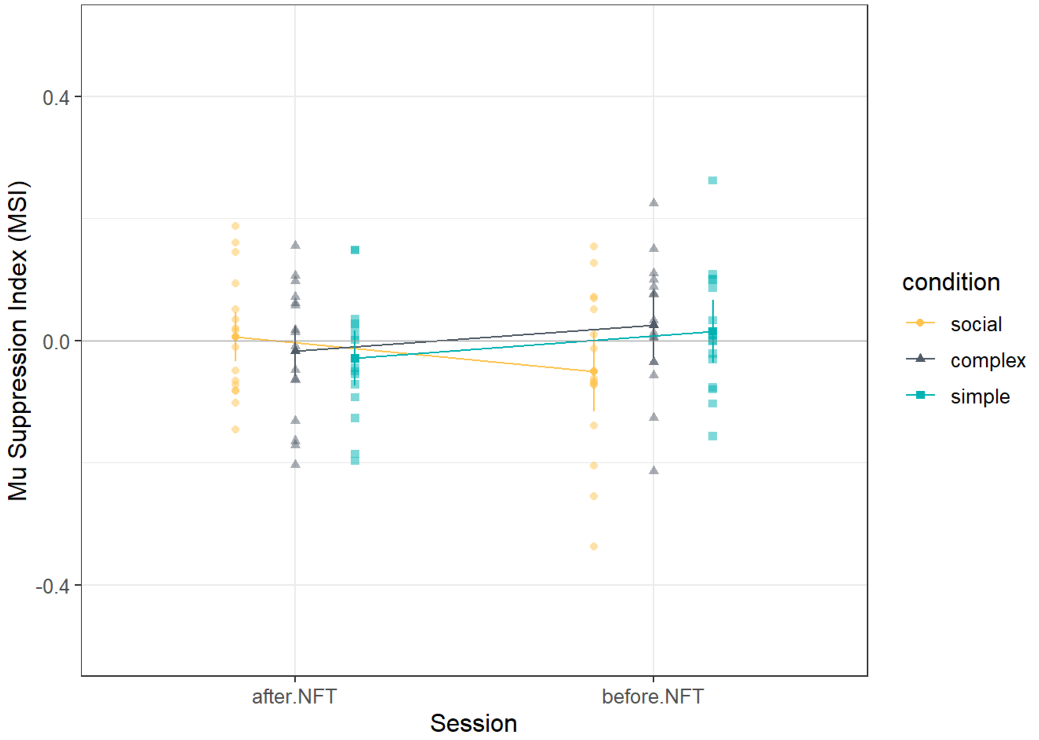


```
#Plot the interaction session*channel-----------
#Gray

afex_plot(mu_all, x = "session", trace = "channel",error = "between", mapping = c("shape"))+geom_hline(yintercept = 0, colour ="#979797", linewidth=0.2)+labs( x = "Session", y = "Mu Suppression Index (MSI)")+ scale_color_manual(values = my_colors)+ theme_bw()+ theme(text = element_text(size = 12, family = "Arial"))
```

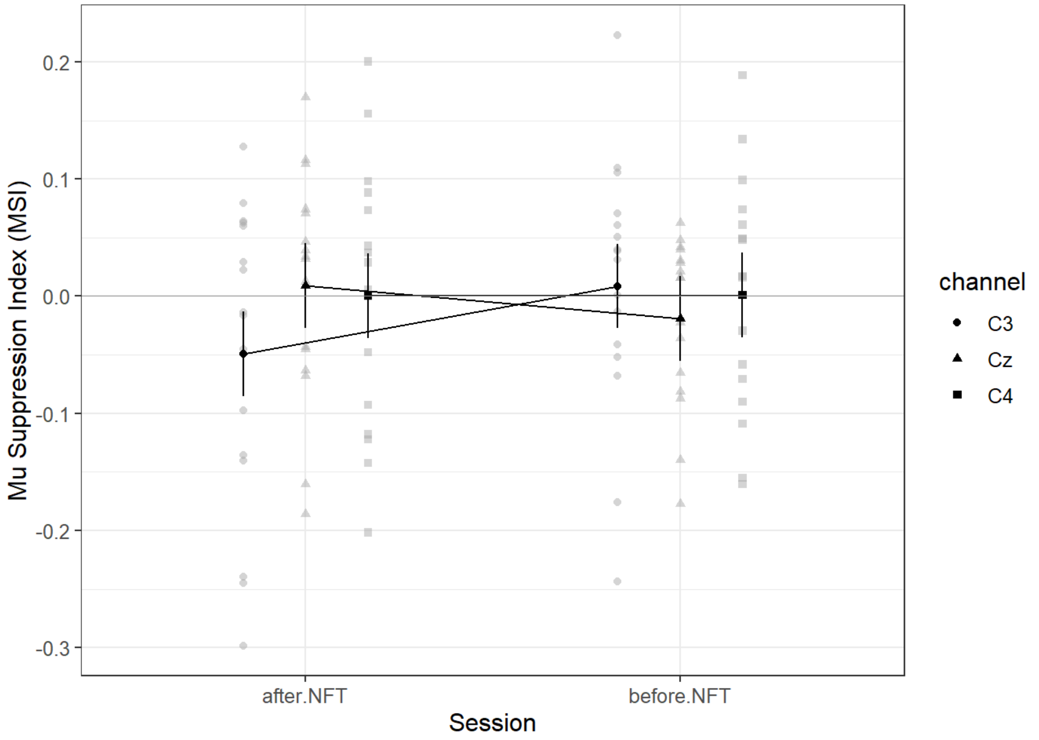

```
#Colored
afex_plot(mu_all, x = "session", trace = "channel",error = "within", mapping = c("shape","colour"))+geom_hline(yintercept
= 0, colour ="#979797", linewidth=0.2)+labs( x = "Session", y = "Mu Suppression Index (MSI)")+ scale_color_manual(values
= my_colors)+ theme_bw()+ theme(text = element_text(size = 12, family = "Arial"))
```

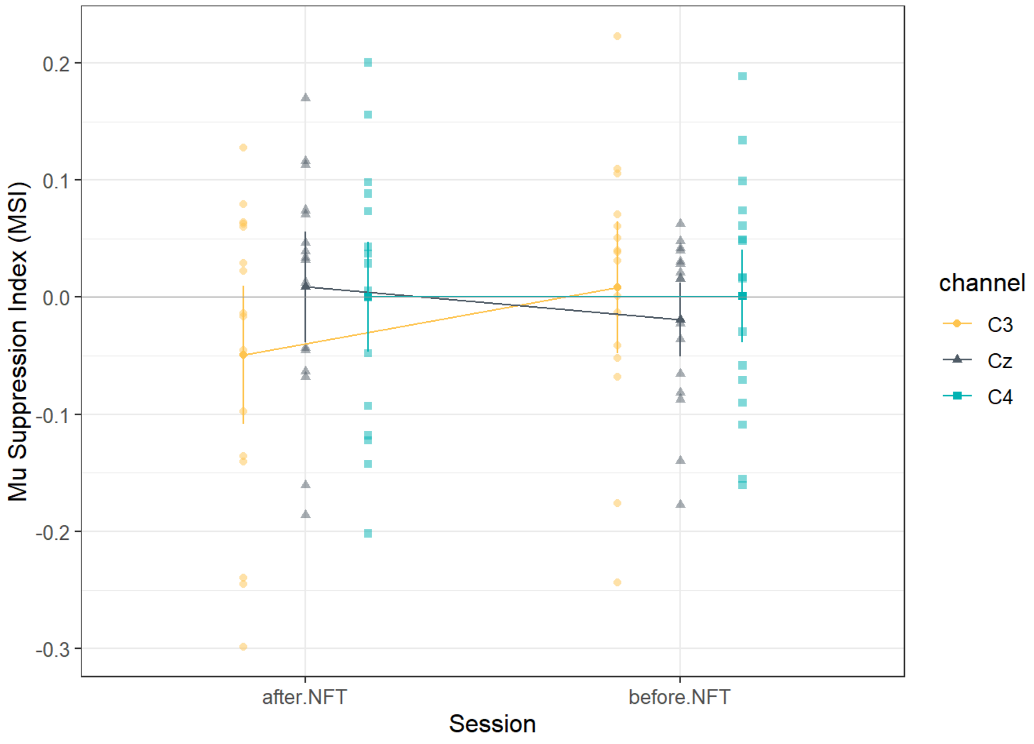

For further investigation of the above interaction between session and condition two one way Anova and three extra t-tests were performed

Following upon the interaction

```
# one way Anova----

EEG<-read.csv(file=("C:/Users/dastg/Desktop/Main-Projects/Empra_2019_Dastgheib/Statistical_Analysis/Input/15VP-NFT/EEG-NF
T-onlyC3C4Cz.csv"),header = TRUE)

#Create an object which has all the data but only for one session

#before

EEGbefore<-EEG %>% filter(session=="before NFT")

mu_allbefore <- aov_ez("VP","mu", EEGbefore,within=c("condition", "channel"))

names(mu_allbefore)
```

```
## [1] "anova_table" "aov"         "Anova"       "lm"          "data"
```

```
summary(mu_allbefore)
```

```
##
## Univariate Type III Repeated-Measures ANOVA Assuming Sphericity
##
##                      Sum Sq num Df Error SS den Df F value  Pr(>F)
## (Intercept)       0.001474      1  0.92347     15  0.0239 0.87911
## condition         0.164261      2  0.88487     30  2.7845 0.07776 .
## channel           0.019510      2  0.34034     30  0.8599 0.43337
## condition:channel 0.026383      4  0.28551     60  1.3861 0.24958
## ---
## Signif. codes:  0 '***' 0.001 '**' 0.01 '*' 0.05 '.' 0.1 ' ' 1
##
##
## Mauchly Tests for Sphericity
##
##                   Test statistic p-value
## condition                0.87349 0.38797
## channel                  0.97630 0.84542
## condition:channel        0.54421 0.52127
##
##
## Greenhouse-Geisser and Huynh-Feldt Corrections
##  for Departure from Sphericity
##
##                    GG eps Pr(>F[GG])
## condition         0.88769    0.08528 .
## channel           0.97685    0.43127
## condition:channel 0.76775    0.25862
## ---
## Signif. codes:  0 '***' 0.001 '**' 0.01 '*' 0.05 '.' 0.1 ' ' 1
##
##                     HF eps Pr(>F[HF])
## condition         0.9983743  0.0778637
## channel           1.1213540  0.4333726
## condition:channel 0.9878459  0.2500773
```

```
#after

EEGafter<-EEG %>% filter(session=="after NFT")

mu_allafter <- aov_ez("VP","mu", EEGafter,within=c("condition", "channel"))

names(mu_allafter)
```

```
## [1] "anova_table" "aov"         "Anova"       "lm"          "data"
```

```
summary(mu_allafter)
```

```
##
## Univariate Type III Repeated-Measures ANOVA Assuming Sphericity
##
##                      Sum Sq num Df Error SS den Df F value Pr(>F)
## (Intercept)       0.025582      1  1.13930     15  0.3368 0.5703
## condition         0.030631      2  0.28379     30  1.6191 0.2149
## channel           0.094394      2  0.62727     30  2.2572 0.1221
## condition:channel 0.021563      4  0.18639     60  1.7353 0.1540
##
##
## Mauchly Tests for Sphericity
##
##                   Test statistic p-value
## condition                0.89049 0.44403
## channel                  0.97850 0.85885
## condition:channel        0.22962 0.02049
##
##
## Greenhouse-Geisser and Huynh-Feldt Corrections
##  for Departure from Sphericity
##
##                    GG eps Pr(>F[GG])
## condition         0.90130     0.2177
## channel           0.97895     0.1234
## condition:channel 0.60286     0.1853
##
##                      HF eps Pr(>F[HF])
## condition         1.0169279  0.2149167
## channel           1.1242979  0.1221235
## condition:channel 0.7265125  0.1751690
```

```
#t-tests to answer whether there was significant effect of session in different conditions--------

#Simple condition
simple<-subset(EEG, condition=="simple", select = c(VP,mu,channel,session))
simple <- simple %>% group_by(VP, session) %>% summarise(Mean = mean(mu),
                                                          SD = sd(mu),
                                                          N = length(mu),
                                                          SE = SD/sqrt(N),
                                                          CI = SE*2.13)

test1 <- t.test(Mean ~ session, data = simple , paired = TRUE, na.rm = TRUE)

test1
```

```
##
##  Paired t-test
##
## data:  Mean by session
## t = -1.4414, df = 15, p-value = 0.17
## alternative hypothesis: true mean difference is not equal to 0
## 95 percent confidence interval:
##  -0.10883595  0.02101852
## sample estimates:
## mean difference
##     -0.04390871
```

```
#Complex condition

complex<-subset(EEG, condition=="complex", select = c(VP,mu,channel,session))

complex <- complex %>% group_by(VP, session) %>% summarise(Mean = mean(mu),
                                                           SD = sd(mu),
                                                           N = length(mu),
                                                           SE = SD/sqrt(N),
                                                           CI = SE*2.13)

test2 <- t.test(Mean ~ session, data = complex , paired = TRUE, na.rm = TRUE)

test2
```

```
##
##  Paired t-test
##
## data:  Mean by session
## t = -1.297, df = 15, p-value = 0.2142
## alternative hypothesis: true mean difference is not equal to 0
## 95 percent confidence interval:
##  -0.11406359  0.02776379
## sample estimates:
## mean difference
##      -0.0431499
```

```
#Social condition

social<-subset(EEG, condition=="social", select = c(VP,mu,channel,session))

social <- social %>% group_by(VP, session) %>% summarise(Mean = mean(mu),
                                                           SD = sd(mu),
                                                           N = length(mu),
                                                           SE = SD/sqrt(N),
                                                           CI = SE*2.13)

test3 <- t.test(Mean ~ session, data = social , paired = TRUE, na.rm = TRUE)

test3
```

```
##
##  Paired t-test
##
## data:  Mean by session
## t = 1.653, df = 15, p-value = 0.1191
## alternative hypothesis: true mean difference is not equal to 0
## 95 percent confidence interval:
##  -0.01640309  0.12974340
## sample estimates:
## mean difference
##      0.05667016
```

ANOVA for Beta and Theta

```
#------------ANOVAS and Post hocs (Beta & Theta) ----------------
#---------------Beta----------------

beta_all <- aov_ez("VP","beta", EEG,within=c("condition", "channel","session"))

names(beta_all)
```

```
## [1] "anova_table" "aov"         "Anova"       "lm"          "data"
```

```
summary(beta_all)
```

```
##
## Univariate Type III Repeated-Measures ANOVA Assuming Sphericity
##
##                              Sum Sq num Df Error SS den Df F value  Pr(>F)
## (Intercept)                0.019682      1  0.62084     15  0.4755 0.50098
## condition                  0.000412      2  0.24218     30  0.0255 0.97483
## channel                    0.000653      2  0.14161     30  0.0692 0.93331
## session                    0.033361      1  0.26594     15  1.8817 0.19031
## condition:channel          0.009720      4  0.07849     60  1.8576 0.12969
## condition:session          0.042915      2  0.19692     30  3.2689 0.05196 .
## channel:session            0.002605      2  0.10515     30  0.3716 0.69276
## condition:channel:session 0.002288      4  0.05782     60  0.5936 0.66861
## ---
## Signif. codes:  0 '***' 0.001 '**' 0.01 '*' 0.05 '.' 0.1 ' ' 1
##
##
## Mauchly Tests for Sphericity
##
##                            Test statistic p-value
## condition                         0.69074 0.07502
## channel                           0.97726 0.85129
## condition:channel                 0.32394 0.09018
## condition:session                 0.49622 0.00741
## channel:session                   0.96826 0.79786
## condition:channel:session         0.27948 0.04891
##
##
## Greenhouse-Geisser and Huynh-Feldt Corrections
##  for Departure from Sphericity
##
##                             GG eps Pr(>F[GG])
## condition                  0.76379    0.94732
## channel                    0.97777    0.93009
## condition:channel          0.65748    0.15876
## condition:session          0.66499    0.07573 .
## channel:session            0.96923    0.68638
## condition:channel:session 0.60078    0.58738
## ---
## Signif. codes:  0 '***' 0.001 '**' 0.01 '*' 0.05 '.' 0.1 ' ' 1
##
##                              HF eps Pr(>F[HF])
## condition                  0.8328587 0.95766111
## channel                    1.1226442 0.93330540
## condition:channel          0.8099899 0.14514185
## condition:session          0.7051798 0.07240938
## channel:session            1.1107199 0.69276059
## condition:channel:session 0.7233991 0.61670812
```

```
capture.output(beta_all, file = "C:/Users/dastg/Desktop/Main-Projects/Empra_2019_Dastgheib/Statistical_Analysis/Input/15V
P-NFT/Output/Tests/NFT-ANOVA-Beta.txt")

#---------------Theta----------------

theta_all <- aov_ez("VP","theta", EEG,within=c("condition", "channel","session"))

names(theta_all)
```

```
## [1] "anova_table" "aov"         "Anova"       "lm"          "data"
```

```
summary(theta_all)
```

```
##
## Univariate Type III Repeated-Measures ANOVA Assuming Sphericity
##
##                              Sum Sq num Df Error SS den Df F value  Pr(>F)
## (Intercept)                0.101182      1  0.50836     15  2.9855 0.10454
## condition                  0.040738      2  0.48437     30  1.2616 0.29781
## channel                    0.019962      2  0.23236     30  1.2887 0.29045
## session                    0.000088      1  0.45157     15  0.0029 0.95766
## condition:channel          0.012699      4  0.17346     60  1.0982 0.36581
## condition:session          0.065158      2  0.21475     30  4.5512 0.01878 *
## channel:session            0.001435      2  0.21012     30  0.1024 0.90295
## condition:channel:session 0.013621      4  0.09733     60  2.0991 0.09205 .
## ---
## Signif. codes:  0 '***' 0.001 '**' 0.01 '*' 0.05 '.' 0.1 ' ' 1
##
##
## Mauchly Tests for Sphericity
##
##                            Test statistic p-value
## condition                         0.33143 0.00044
## channel                           0.54065 0.01350
## condition:channel                 0.24420 0.02715
## condition:session                 0.88887 0.43840
## channel:session                   0.27914 0.00013
## condition:channel:session         0.39705 0.19575
##
##
## Greenhouse-Geisser and Huynh-Feldt Corrections
##  for Departure from Sphericity
##
##                             GG eps Pr(>F[GG])
## condition                  0.59932    0.28586
## channel                    0.68524    0.28414
## condition:channel          0.59461    0.35286
## condition:session          0.89998    0.02281 *
## channel:session            0.58110    0.79022
## condition:channel:session 0.68465    0.12013
## ---
## Signif. codes:  0 '***' 0.001 '**' 0.01 '*' 0.05 '.' 0.1 ' ' 1
##
##                               HF eps Pr(>F[HF])
## condition                  0.6223327 0.28712449
## channel                    0.7310452 0.28568136
## condition:channel          0.7141609 0.35845387
## condition:session          1.0151308 0.01878356
## channel:session            0.5996381 0.79768759
## condition:channel:session 0.8526333 0.10421242
```

```
capture.output(theta_all, file = "C:/Users/dastg/Desktop/Main-Projects/Empra_2019_Dastgheib/Statistical_Analysis/Input/15
VP-NFT/Output/Tests/NFT-ANOVA-theta.txt")

thetaTukey = lsmeans(theta_all,pairwise ~ session:condition,adjust = "tukey")
thetaTukey$contrasts
```

```
##  contrast                                estimate      SE df t.ratio p.value
##  after.NFT social - before.NFT social    0.04355 0.03164 15   1.377  0.7396
##  after.NFT social - after.NFT complex    0.05957 0.02024 15   2.944  0.0870
##  after.NFT social - before.NFT complex   0.04193 0.02834 15   1.480  0.6812
##  after.NFT social - after.NFT simple     0.04497 0.02109 15   2.132  0.3231
##  after.NFT social - before.NFT simple    0.02237 0.04048 15   0.553  0.9927
##  before.NFT social - after.NFT complex   0.01602 0.02823 15   0.568  0.9918
##  before.NFT social - before.NFT complex -0.00162 0.02721 15  -0.059  1.0000
##  before.NFT social - after.NFT simple    0.00142 0.02824 15   0.050  1.0000
##  before.NFT social - before.NFT simple  -0.02118 0.03065 15  -0.691  0.9802
##  after.NFT complex - before.NFT complex -0.01764 0.01515 15  -1.164  0.8465
##  after.NFT complex - after.NFT simple   -0.01461 0.00772 15  -1.893  0.4424
##  after.NFT complex - before.NFT simple  -0.03721 0.02490 15  -1.494  0.6728
##  before.NFT complex - after.NFT simple   0.00303 0.01302 15   0.233  0.9999
##  before.NFT complex - before.NFT simple -0.01957 0.01786 15  -1.096  0.8757
##  after.NFT simple - before.NFT simple   -0.02260 0.02491 15  -0.907  0.9387
##
## Results are averaged over the levels of: channel
## P value adjustment: tukey method for comparing a family of 6 estimates
```

```
thetaposthoc <-thetaTukey
capture.output(thetaposthoc, file = "C:/Users/dastg/Desktop/Main-Projects/Empra_2019_Dastgheib/Statistical_Analysis/Input/15VP-NFT/Output/Tests/thetaposthoc.txt")
```

T-test against Zero In this chunk, we investigate if mu suppression happend during the human action observation before and after neurofeedback. Thus, we compare the MSI average in every condition before and after NFT to zero. considering the formula of the MSI, if any MSI in any condition is significantly less than zero, mu suppression has happend meaning that the Mu rhythm amplitude during watching that specific condition was less than the mu rhythm amplitude during the observation of baseline scenario.

```
#--------------- T-test against Zero ----------------

#This Analysis has two parts: 1. T-test against zero for all participants  MSI after neurofeedback 2. T-test against zero
for all participants  MSI before neurofeedback.

#--------------------Part1: Before NFT------------------------------

#Load the data set:

EEG<-read.csv(file=("C:/Users/dastg/Desktop/Main-Projects/Empra_2019_Dastgheib/Statistical_Analysis/Input/15VP-NFT/EEG-NF
T-onlyC3C4Cz.csv"),header = TRUE)

#Create an object which has all the data but only for one session

EEGbefore<-EEG %>% filter(session=="before NFT")

#------------Condition=social-----------------

#Create an object which has all the data but only for one condition

EEGsocialbefore<-EEGbefore %>% filter(condition=="social")

# Calculate mean across C3, C4 and Cz

#Before,we need the t-distribution since n<30, central limit theorem does not work.
confidence_level <-0.95 # a is considered <0.05 before starting the research => thus, a/2 should be 0.05/2 =0.025

n<-16

z<-qt((1+confidence_level)/2, df= n-1 )

#Then we calculate a grand mean across the electrodes:

EEGsocialbefore_acrossCs <- EEGsocialbefore %>% group_by(`VP`) %>% summarise(mean_mu = mean(mu),
                                                                          SD = sd(mu),
                                                                          N  = length(mu),
                                                                          SE = SD/sqrt(N),
                                                                          CI = SE*z)


socialBVszero<-t.test(EEGsocialbefore_acrossCs$mean_mu, mean_mu=0, alternative = "two.sided")

t.test(EEGsocialbefore_acrossCs$mean_mu, mean_mu=0, alternative = "two.sided")
```

```
##
##  One Sample t-test
##
## data:  EEGsocialbefore_acrossCs$mean_mu
## t = -1.4912, df = 15, p-value = 0.1566
## alternative hypothesis: true mean is not equal to 0
## 95 percent confidence interval:
##  -0.12276474  0.02169635
## sample estimates:
##  mean of x
## -0.0505342
```

```
capture.output(socialBVszero, file = "C:/Users/dastg/Desktop/Main-Projects/Empra_2019_Dastgheib/Statistical_Analysis/Inpu
t/15VP-NFT/Output/Tests/Tagainst0beforesocial.txt")

#----------------Effect size---------------
#use lsr package:
install.packages("lsr")
library(lsr)
cohensD(x=EEGsocialbefore_acrossCs$mean_mu,mu=0)
```

```
## [1] 0.3728031
```

```
#---------------For every channels----------------


C3EEGbeforesocial <-subset(EEGbefore, subset = condition== "social" & channel== "C3")
C3socialVszero<-t.test(C3EEGbeforesocial$mu, mu=0, alternative = "two.sided")


C4EEGbeforesocial <-subset(EEGbefore, subset = condition== "social" & channel== "C4")
C4socialVszero<-t.test(C4EEGbeforesocial$mu, mu=0, alternative = "two.sided")


CzEEGbeforesocial <-subset(EEGbefore, subset = condition== "social" & channel== "Cz")
CzsocialVszero<-t.test(CzEEGbeforesocial$mu, mu=0, alternative = "two.sided")

#------------Condition=Complex-----------------

EEGbeforecomplex<-EEGbefore %>% filter(condition=="complex")

EEGbeforecomplex_acrossCs <- EEGbeforecomplex %>% group_by(`VP`) %>% summarise(mean_mu = mean(mu),
                                                                        SD = sd(mu),
                                                                        N  = length(mu),
                                                                        SE = SD/sqrt(N),
                                                                        CI = SE*z)

complexVszerobefore<-t.test(EEGbeforecomplex_acrossCs$mean_mu, mean_mu=0, alternative = "two.sided")
t.test(EEGbeforecomplex_acrossCs$mean_mu, mean_mu=0, alternative = "two.sided")
```

```
##
##  One Sample t-test
##
## data:  EEGbeforecomplex_acrossCs$mean_mu
## t = 0.96951, df = 15, p-value = 0.3477
## alternative hypothesis: true mean is not equal to 0
## 95 percent confidence interval:
##  -0.03115937  0.08315739
## sample estimates:
##  mean of x
## 0.02599901
```

```
#-------------Effect size---------------
#use lsr package:
install.packages("lsr")
library(lsr)
cohensD(x=EEGbeforecomplex_acrossCs$mean_mu,mu=0) #Here is 0.2423773
```

```
## [1] 0.2423773
```

```
mean(EEGbeforecomplex_acrossCs$mean_mu)
```

```
## [1] 0.02599901
```

```
sd(EEGbeforecomplex_acrossCs$mean_mu)
```

```
## [1] 0.1072667
```

```
capture.output(complexVszerobefore, file = "C:/Users/dastg/Desktop/Main-Projects/Empra_2019_Dastgheib/Statistical_Analysi
s/Input/15VP-NFT/Output/Tests/Tagainst0beforecomplex.txt")

#----------------For every channel------------------

C3EEGbeforecomplex <-subset(EEGbefore, subset = condition== "complex" & channel== "C3")
t.test(C3EEGbeforecomplex$mu, mu=0, alternative = "two.sided")
```

```
##
##  One Sample t-test
##
## data:  C3EEGbeforecomplex$mu
## t = 0.76238, df = 15, p-value = 0.4577
## alternative hypothesis: true mean is not equal to 0
## 95 percent confidence interval:
##  -0.04483630  0.09477151
## sample estimates:
## mean of x
## 0.0249676
```

```
C4EEGbeforecomplex <-subset(EEGbefore, subset = condition== "complex" & channel== "C4")
t.test(C4EEGbeforecomplex$mu, mu=0, alternative = "two.sided")
```

```
##
##  One Sample t-test
##
## data:  C4EEGbeforecomplex$mu
## t = 1.5437, df = 15, p-value = 0.1435
## alternative hypothesis: true mean is not equal to 0
## 95 percent confidence interval:
##  -0.02098103  0.13119063
## sample estimates:
## mean of x
## 0.0551048
```

```
CzEEGbeforecomplex <-subset(EEGbefore, subset = condition== "complex" & channel== "Cz")
t.test(CzEEGbeforecomplex$mu, mu=0, alternative = "two.sided")
```

```
##
##  One Sample t-test
##
## data:  CzEEGbeforecomplex$mu
## t = -0.071827, df = 15, p-value = 0.9437
## alternative hypothesis: true mean is not equal to 0
## 95 percent confidence interval:
##  -0.06366162  0.05951087
## sample estimates:
##    mean of x
## -0.002075373
```

```
#------------Condition=Simple-----------------

EEGbeforesimple<-EEGbefore %>% filter(condition=="simple")
EEGbeforesimple_acrossCs <- EEGbeforesimple %>% group_by(`VP`) %>% summarise(mean_mu = mean(mu),
                                                                          SD = sd(mu),
                                                                          N  = length(mu),
                                                                          SE = SD/sqrt(N),
                                                                          CI = SE*z)

simpleVszerobefore<-t.test(EEGbeforesimple_acrossCs$mean_mu, mean_mu=0, alternative = "two.sided")
t.test(EEGbeforesimple_acrossCs$mean_mu, mean_mu=0, alternative = "two.sided")
```

```
##
##  One Sample t-test
##
## data:  EEGbeforesimple_acrossCs$mean_mu
## t = 0.58861, df = 15, p-value = 0.5649
## alternative hypothesis: true mean is not equal to 0
## 95 percent confidence interval:
##  -0.03915484  0.06903087
## sample estimates:
##  mean of x
## 0.01493802
```

```
#------------------Effect size------------------
#use lsr package:
install.packages("lsr")
library(lsr)
cohensD(x=EEGbeforesimple_acrossCs$mean_mu,mu=0) #Here is 0.1471527
```

```
## [1] 0.1471527
```

```
capture.output(simpleVszerobefore, file = "C:/Users/dastg/Desktop/Main-Projects/Empra_2019_Dastgheib/Statistical_Analysi
s/Input/15VP-NFT/Output/Tests/Tagainst0beforesimple.txt")


#----------------For every channel------------------

C3EEGbeforesimple <-subset(EEGbefore, subset = condition== "simple" & channel== "C3")
C3simpleVszero<-t.test(C3EEGbeforesimple$mu, mu=0, alternative = "two.sided")


C4EEGbeforesimple <-subset(EEGbefore, subset = condition== "simple" & channel== "C4")
C4simpleVszero<-t.test(C4EEGbeforesimple$mu, mu=0, alternative = "two.sided")


CzEEGbeforesimple <-subset(EEGbefore, subset = condition== "simple" & channel== "Cz")
CzsimpleVszero<-t.test(CzEEGbeforesimple$mu, mu=0, alternative = "two.sided")

#--------------------------Part2: After NFT--------------------------

#load the data set:

EEG<-read.csv(file=("C:/Users/dastg/Desktop/Main-Projects/Empra_2019_Dastgheib/Statistical_Analysis/Input/15VP-NFT/EEG-NF
T-onlyC3C4Cz.csv"),header = TRUE)

#Create an object which has all the data but only for one session

EEGafter<-EEG %>% filter(session=="after NFT")

#------------Condition=Social-----------------

#Create an object which has all the data but only for one condition

EEGsocialafter<-EEGafter %>% filter(condition=="social")

# Calculate mean across C3, C4 and Cz

#Before,we need the t-distribution since n<30, central limit theorem does not work.
confidence_level <-0.95 # a is considered <0.05 before starting the research => thus, a/2 should be 0.05/2 =0.025

n<-16

z<-qt((1+confidence_level)/2, df= n-1 )

#Then we calculate a grand mean across the electrodes:

EEGsocialafter_acrossCs <- EEGsocialafter %>% group_by(`VP`) %>% summarise(mean_mu = mean(mu),
                                                                       SD = sd(mu),
                                                                       N  = length(mu),
                                                                       SE = SD/sqrt(N),
                                                                       CI = SE*z)

socialafterVszero<-t.test(EEGsocialafter_acrossCs$mean_mu, mean_mu=0, alternative = "two.sided")

t.test(EEGsocialafter_acrossCs$mean_mu, mean_mu=0, alternative = "two.sided")
```

```
##
##  One Sample t-test
##
## data:  EEGsocialafter_acrossCs$mean_mu
## t = 0.24407, df = 15, p-value = 0.8105
## alternative hypothesis: true mean is not equal to 0
## 95 percent confidence interval:
##  -0.04744799  0.05971991
## sample estimates:
##   mean of x
## 0.006135959
```

```
capture.output(socialafterVszero, file = "C:/Users/dastg/Desktop/Main-Projects/Empra_2019_Dastgheib/Statistical_Analysis/
Input/15VP-NFT/Output/Tests/Tagainst0aftersocial.txt")

#----------------Effect size---------------
#use lsr package:
install.packages("lsr")
library(lsr)
cohensD(x=EEGsocialafter_acrossCs$mean_mu,mu=0)
```

```
## [1] 0.06101868
```

```
#Here it equals = 0.06101868

#-------------------------For every channels------------


C3EEGaftersocial <-subset(EEGafter, subset = condition== "social" & channel== "C3")
t.test(C3EEGaftersocial$mu, mu=0, alternative = "two.sided")
```

```
##
##  One Sample t-test
##
## data:  C3EEGaftersocial$mu
## t = -0.31677, df = 15, p-value = 0.7558
## alternative hypothesis: true mean is not equal to 0
## 95 percent confidence interval:
##  -0.07219082  0.05350953
## sample estimates:
##    mean of x
## -0.009340648
```

```
C4EEGaftersocial <-subset(EEGafter, subset = condition== "social" & channel== "C4")
t.test(C4EEGaftersocial$mu, mu=0, alternative = "two.sided")
```

```
##
##  One Sample t-test
##
## data:  C4EEGaftersocial$mu
## t = 0.34173, df = 15, p-value = 0.7373
## alternative hypothesis: true mean is not equal to 0
## 95 percent confidence interval:
##  -0.05885026  0.08132412
## sample estimates:
##  mean of x
## 0.01123693
```

```
CzEEGaftersocial <-subset(EEGafter, subset = condition== "social" & channel== "Cz")
t.test(CzEEGaftersocial$mu, mu=0, alternative = "two.sided")
```

```
##
##  One Sample t-test
##
## data:  CzEEGaftersocial$mu
## t = 0.55092, df = 15, p-value = 0.5898
## alternative hypothesis: true mean is not equal to 0
## 95 percent confidence interval:
##  -0.04737024  0.08039343
## sample estimates:
## mean of x
## 0.0165116
```

```
#------------Condition=complex-----------------

EEGaftercomplex<-EEGafter %>% filter(condition=="complex")

EEGaftercomplex_acrossCs <- EEGaftercomplex %>% group_by(`VP`) %>% summarise(mean_mu = mean(mu),
                                                                          SD = sd(mu),
                                                                          N  = length(mu),
                                                                          SE = SD/sqrt(N),
                                                                          CI = SE*z)

complexVszeroafter<-t.test(EEGaftercomplex_acrossCs$mean_mu, mean_mu=0, alternative = "two.sided")
t.test(EEGaftercomplex_acrossCs$mean_mu, mean_mu=0, alternative = "two.sided")
```

```
##
##  One Sample t-test
##
## data:  EEGaftercomplex_acrossCs$mean_mu
## t = -0.62944, df = 15, p-value = 0.5385
## alternative hypothesis: true mean is not equal to 0
## 95 percent confidence interval:
##  -0.07522808  0.04092630
## sample estimates:
##   mean of x
## -0.01715089
```

```
#-----------------Effect size--------------
#use lsr package:
install.packages("lsr")
library(lsr)
cohensD(x=EEGaftercomplex_acrossCs$mean_mu,mu=0) #Here is 0.1573606
```

```
## [1] 0.1573606
```

```
mean(EEGaftercomplex_acrossCs$mean_mu)
```

```
## [1] -0.01715089
```

```
sd(EEGaftercomplex_acrossCs$mean_mu)
```

```
## [1] 0.108991
```

```
capture.output(complexVszeroafter, file = "C:/Users/dastg/Desktop/Main-Projects/Empra_2019_Dastgheib/Statistical_Analysi
s/Input/15VP-NFT/Output/Tests/Tagainst0aftercomplex.txt")

#----------------For every channel------------------

C3EEGaftercomplex <-subset(EEGafter, subset = condition== "complex" & channel== "C3")
t.test(C3EEGaftercomplex$mu, mu=0, alternative = "two.sided")
```

```
##
##  One Sample t-test
##
## data:  C3EEGaftercomplex$mu
## t = -1.4418, df = 15, p-value = 0.1699
## alternative hypothesis: true mean is not equal to 0
## 95 percent confidence interval:
##  -0.1362789  0.0263031
## sample estimates:
##   mean of x
## -0.05498792
```

```
C4EEGaftercomplex <-subset(EEGafter, subset = condition== "complex" & channel== "C4")
t.test(C4EEGaftercomplex$mu, mu=0, alternative = "two.sided")
```

```
##
##  One Sample t-test
##
## data:  C4EEGaftercomplex$mu
## t = 0.10062, df = 15, p-value = 0.9212
## alternative hypothesis: true mean is not equal to 0
## 95 percent confidence interval:
##  -0.07471913  0.08212309
## sample estimates:
##   mean of x
## 0.003701981
```

```
CzEEGaftercomplex <-subset(EEGafter, subset = condition== "complex" & channel== "Cz")
t.test(CzEEGaftercomplex$mu, mu=0, alternative = "two.sided")
```

```
##
##  One Sample t-test
##
## data:  CzEEGaftercomplex$mu
## t = -0.0054008, df = 15, p-value = 0.9958
## alternative hypothesis: true mean is not equal to 0
## 95 percent confidence interval:
##  -0.06596637  0.06563292
## sample estimates:
##    mean of x
## -0.000166727
```

```
#------------Condition=Simple-----------------

EEGaftersimple<-EEGafter %>% filter(condition=="simple")
EEGaftersimple_acrossCs <- EEGaftersimple %>% group_by(`VP`) %>% summarise(mean_mu = mean(mu),
                                                                        SD = sd(mu),
                                                                        N  = length(mu),
                                                                        SE = SD/sqrt(N),
                                                                        CI = SE*z)

simpleVszeroafter<-t.test(EEGaftersimple_acrossCs$mean_mu, mean_mu=0, alternative = "two.sided")
t.test(EEGaftersimple_acrossCs$mean_mu, mean_mu=0, alternative = "two.sided")
```

```
##
##  One Sample t-test
##
## data:  EEGaftersimple_acrossCs$mean_mu
## t = -1.1807, df = 15, p-value = 0.2561
## alternative hypothesis: true mean is not equal to 0
## 95 percent confidence interval:
##  -0.08127015  0.02332875
## sample estimates:
##  mean of x
## -0.0289707
```

```
#------------Effect size--------------------
#use lsr package:
install.packages("lsr")
library(lsr)
cohensD(x=EEGaftersimple_acrossCs$mean_mu,mu=0) #Here is 0.2951732
```

```
## [1] 0.2951732
```

```
capture.output(simpleVszeroafter, file = "C:/Users/dastg/Desktop/Main-Projects/Empra_2019_Dastgheib/Statistical_Analysis/
Input/15VP-NFT/Output/Tests/Tagainst0aftersimple.txt")


#----------------For every channel------------------

C3EEGaftersimple <-subset(EEGafter, subset = condition== "simple" & channel== "C3")
t.test(C3EEGaftersimple$mu, mu=0, alternative = "two.sided")
```

```
##
##  One Sample t-test
##
## data:  C3EEGaftersimple$mu
## t = -2.1241, df = 15, p-value = 0.0507
## alternative hypothesis: true mean is not equal to 0
## 95 percent confidence interval:
##  -0.1667794411  0.0002888847
## sample estimates:
##   mean of x
## -0.08324528
```

```
C4EEGaftersimple <-subset(EEGafter, subset = condition== "simple" & channel== "C4")
t.test(C4EEGaftersimple$mu, mu=0, alternative = "two.sided")
```

```
##
##  One Sample t-test
##
## data:  C4EEGaftersimple$mu
## t = -0.59057, df = 15, p-value = 0.5636
## alternative hypothesis: true mean is not equal to 0
## 95 percent confidence interval:
##  -0.06495378  0.03676904
## sample estimates:
##   mean of x
## -0.01409237
```

```
CzEEGaftersimple <-subset(EEGafter, subset = condition== "simple" & channel== "Cz")
t.test(CzEEGaftersimple$mu, mu=0, alternative = "two.sided")
```

```
##
##  One Sample t-test
##
## data:  CzEEGaftersimple$mu
## t = 0.42415, df = 15, p-value = 0.6775
## alternative hypothesis: true mean is not equal to 0
## 95 percent confidence interval:
##  -0.0419649  0.0628160
## sample estimates:
##  mean of x
## 0.01042555
```

Overlayed graphs

```
#-------------overlayed graphs---------------

#I want to have scatter plots graphs of individual MSIs overlayed by mean and condifence interval of every condition and every electrode.

#-------------bofore NFT---------------

#First for the bofore NFT:

EEG<-read.csv(file=("C:/Users/dastg/Desktop/Main-Projects/Empra_2019_Dastgheib/Statistical_Analysis/Input/15VP-NFT/EEG-NFT-onlyC3C4Cz.csv"),header = TRUE)

#Create an object which has all the data but only for one session

EEGbefore<-EEG %>% filter(session=="before NFT")


EEGbefore$channel <- as.factor(EEGbefore$channel)
levels(EEGbefore$channel)
```

```
## [1] "C3" "C4" "Cz"
```

```
#re-order factor levels for region
EEGbefore$channel <- factor(EEGbefore$channel, levels=c("C3", "Cz", "C4"))
#display factor levels for region
levels(EEGbefore$channel)
```

```
## [1] "C3" "Cz" "C4"
```

```
EEGbefore$condition <- as.factor(EEGbefore$condition)
levels(EEGbefore$condition)
```

```
## [1] "complex" "simple"  "social"
```

```
#re-order factor levels for region
EEGbefore$condition <- factor(EEGbefore$condition, levels=c("simple", "complex", "social"))
#display factor levels for region
levels(EEGbefore$condition)
```

```
## [1] "simple"  "complex" "social"
```

```
line2 <- ggplot( EEGbefore, aes(channel, mu))

line2 + stat_summary(fun = mean, geom = "point") + stat_summary(fun = mean, geom = "line", aes(group = condition))+  facet_wrap( ~condition) +stat_summary(fun.data = mean_cl_normal, geom = "pointrange")+ labs(title= "MSIs with non-biological movements (moving balls) as baseline", x = "Channel", y = "Mu Suppression Index (MSI)", colour = "Conditon")+ theme_bw()
```

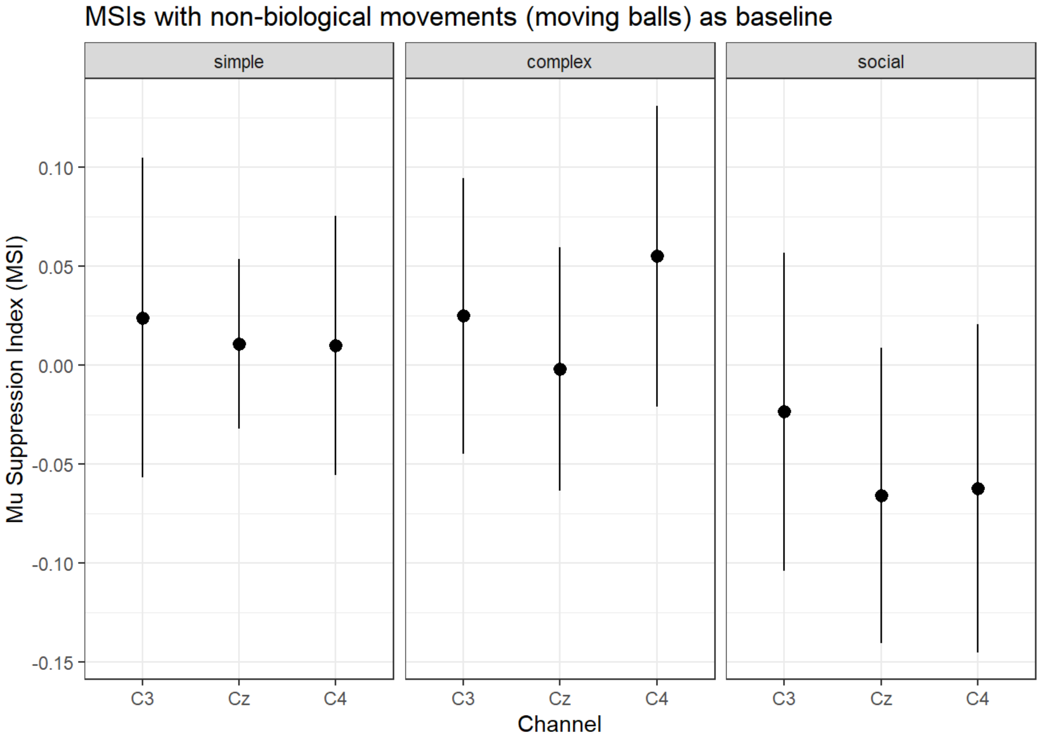


```
 scatter1 <- ggplot(EEGbefore, aes(channel,mu))

scatter1 + geom_point(color="#fec44f")+facet_wrap( ~condition) + labs(title= "MSIs with non-biological movements (moving
balls) as baseline", x = "Channel", y = "Mu Suppression Index (MSI)", colour = "Conditon")+ theme_bw()
```

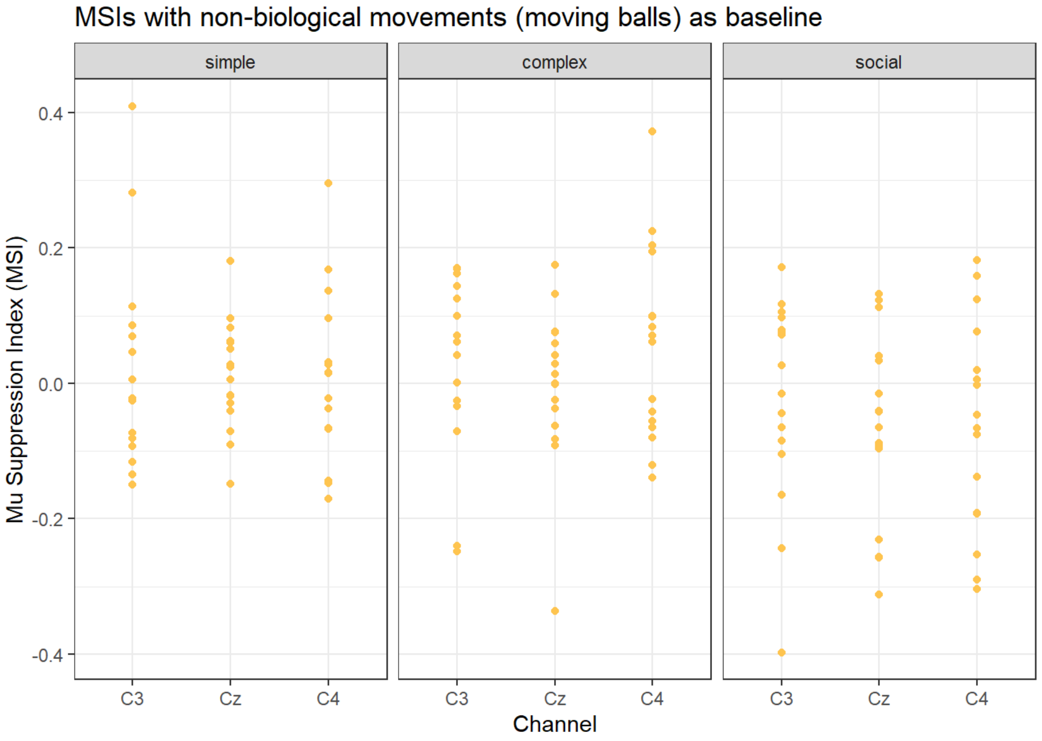


```
#trying to overy lay

windowsFonts("Arial" = windowsFont("Arial"))
ggplot(EEGbefore, aes(x=channel, y=mu)) +geom_hline(yintercept = 0, colour ="#bdbdbd")+ geom_point(aes(y= mu),color="#fec
44f",position =position_dodge(width = 0.5))+ stat_summary(fun = mean, geom = "point") + stat_summary(fun = mean, geom =
"line", aes(group = condition))+  facet_wrap( ~condition) +stat_summary(fun.data = mean_cl_normal, geom = "pointrange")+
labs( x = "Channel", y = "Mu Suppression Index (MSI)", colour = "Conditon")+ theme_bw()+scale_y_continuous( limits = c(-
0.5,0.5))+ theme(text = element_text(size = 15, family = "Arial"))
```

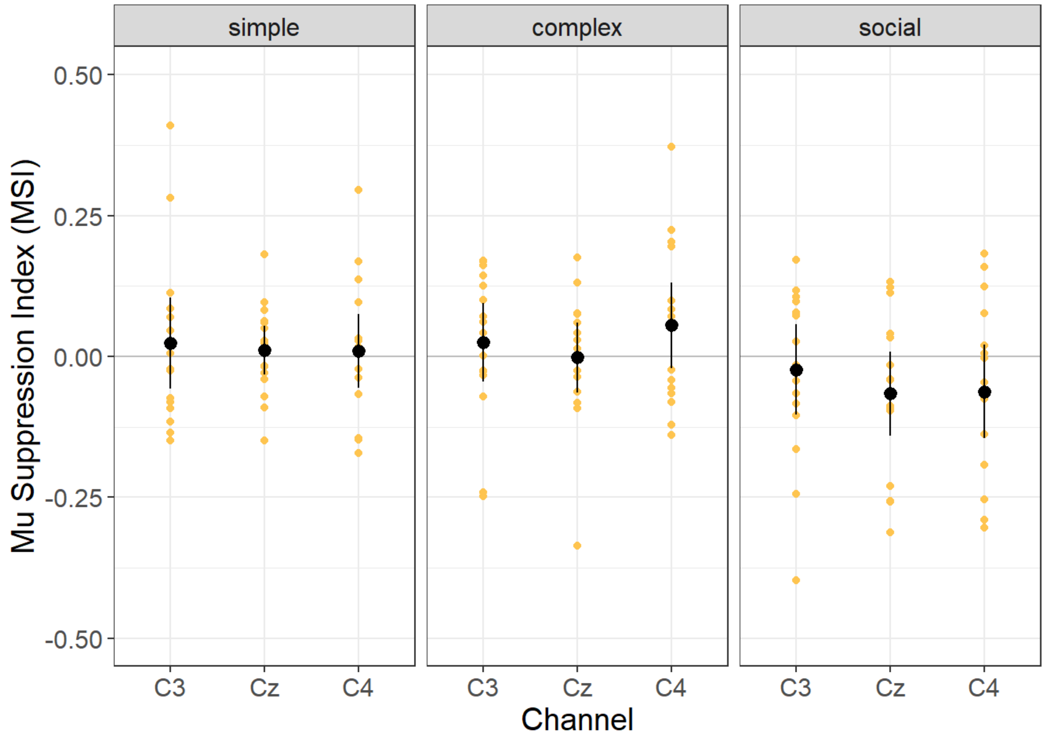


```
#title= "MSIs with non-biological movements (moving balls) as baseline"

ggsave("C:/Users/dastg/Desktop/Main-Projects/Empra_2019_Dastgheib/Statistical_Analysis/Input/15VP-NFT/Output/Plots/MSI-beforeNFT-overlayed-plot.png", width = 8, height = 4, dpi = 300)


#-------------after NFT---------------
#Second for the after NFT:

EEG<-read.csv(file=("C:/Users/dastg/Desktop/Main-Projects/Empra_2019_Dastgheib/Statistical_Analysis/Input/15VP-NFT/EEG-NFT-onlyC3C4Cz.csv"),header = TRUE)

#Create an object which has all the data but only for one session

EEGafter<-EEG %>% filter(session=="after NFT")


EEGafter$channel <- as.factor(EEGafter$channel)
levels(EEGafter$channel)
```

```
## [1] "C3" "C4" "Cz"
```

```
#re-order factor levels for region
EEGafter$channel <- factor(EEGafter$channel, levels=c("C3", "Cz", "C4"))
#display factor levels for region
levels(EEGafter$channel)
```

```
## [1] "C3" "Cz" "C4"
```

```
EEGafter$condition <- as.factor(EEGafter$condition)
levels(EEGafter$condition)
```

```
## [1] "complex" "simple"  "social"
```

```
#re-order factor levels for region
EEGafter$condition <- factor(EEGafter$condition, levels=c("simple", "complex", "social"))
#display factor levels for region
levels(EEGafter$condition)
```

```
## [1] "simple"  "complex" "social"
```

```
line2 <- ggplot( EEGafter, aes(channel, mu))

line2 + stat_summary(fun = mean, geom = "point") + stat_summary(fun = mean, geom = "line", aes(group = condition))+  face
t_wrap( ~condition) +stat_summary(fun.data = mean_cl_normal, geom = "pointrange")+ labs(title= "MSIs with non-biological
movements (moving balls) as baseline", x = "Channel", y = "Mu Suppression Index (MSI)", colour = "Conditon")+ theme_bw()
```

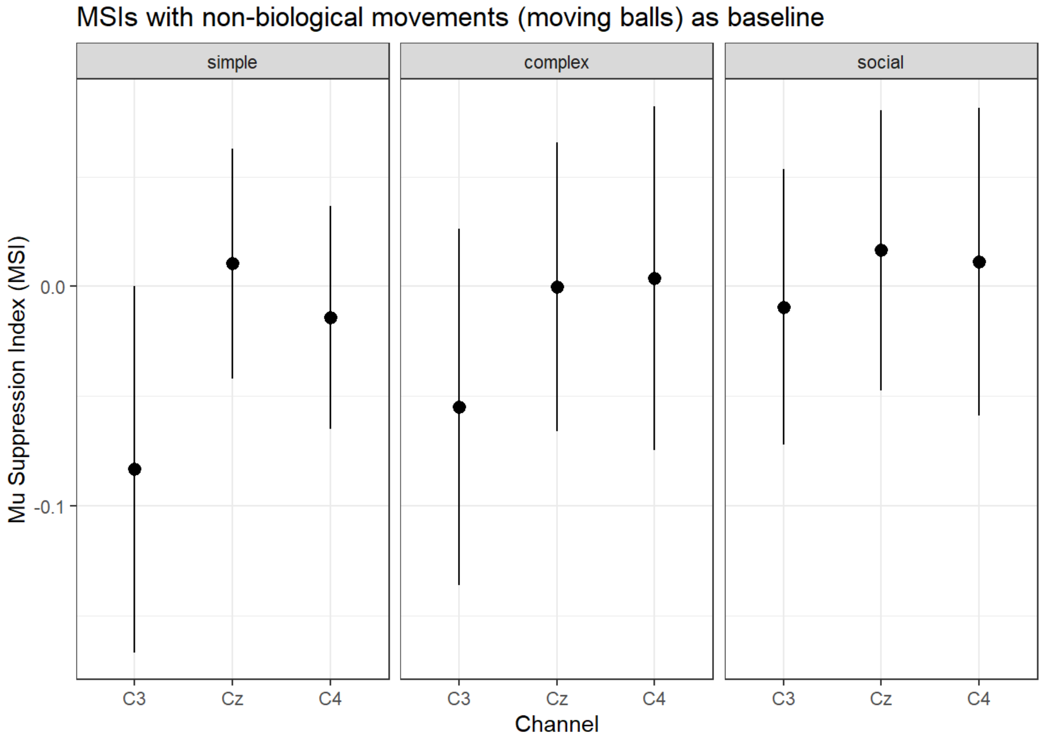


```
 scatter1 <- ggplot(EEGafter, aes(channel,mu))

scatter1 + geom_point(color="#fec44f")+facet_wrap( ~condition) + labs(title= "MSIs with non-biological movements (moving
balls) as baseline", x = "Channel", y = "Mu Suppression Index (MSI)", colour = "Conditon")+ theme_bw()
```

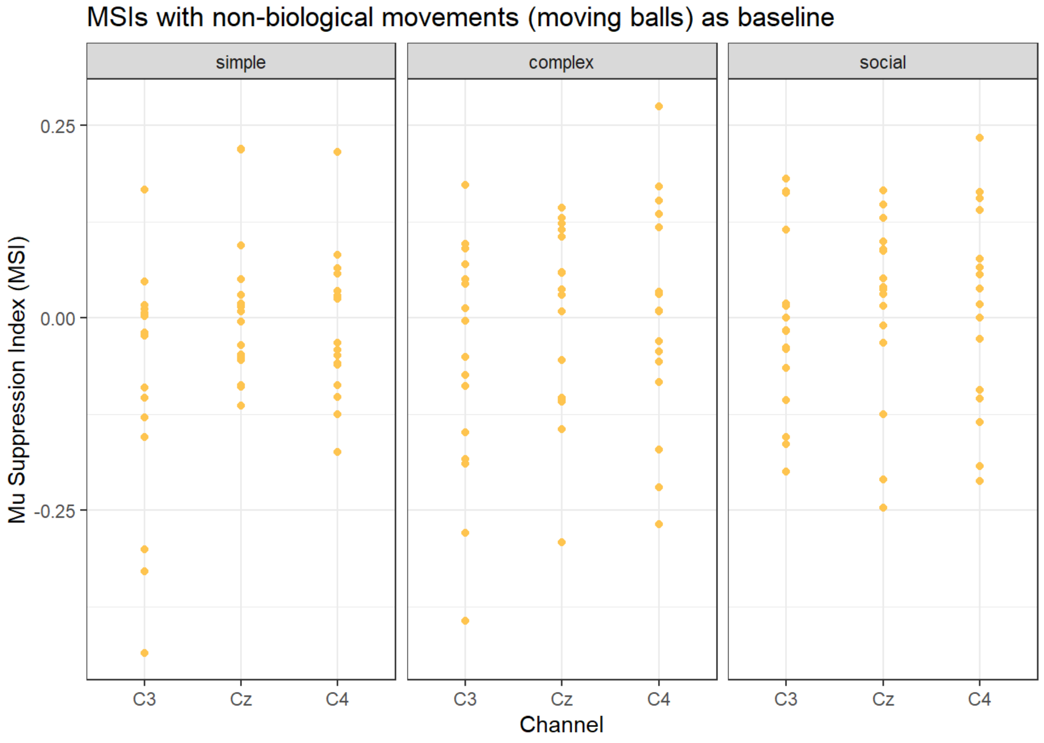

```
#trying to overy lay
windowsFonts("Arial" = windowsFont("Arial"))
ggplot(EEGafter, aes(x=channel, y=mu)) +geom_hline(yintercept = 0, colour ="#bdbdbd")+ geom_point(aes(y= mu),color="#fec4
4f",position =position_dodge(width = 0.5))+ stat_summary(fun = mean, geom = "point") + stat_summary(fun = mean, geom = "l
ine", aes(group = condition))+  facet_wrap( ~condition) +stat_summary(fun.data = mean_cl_normal, geom = "pointrange")+ la
bs( x = "Channel", y = "Mu Suppression Index (MSI)", colour = "Conditon")+ theme_bw()+scale_y_continuous( limits = c(-0.
5,0.5))+ theme(text = element_text(size = 15, family = "Arial"))
```

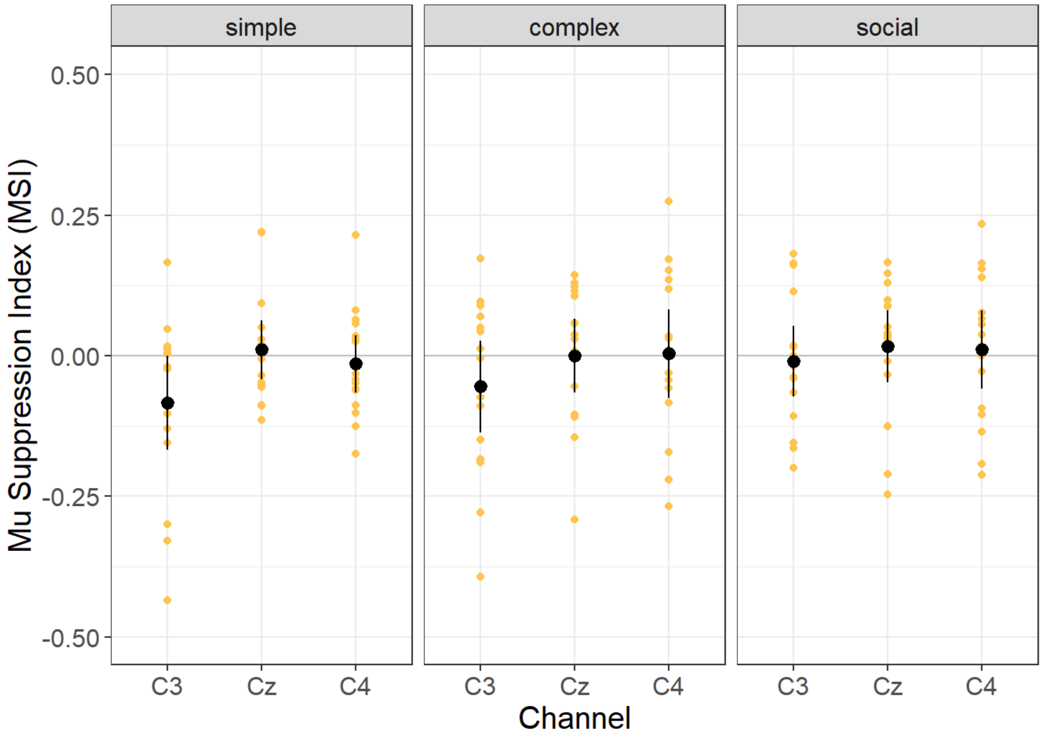


```
#title= "MSIs with non-biological movements (moving balls) as baseline"

ggsave("C:/Users/dastg/Desktop/Main-Projects/Empra_2019_Dastgheib/Statistical_Analysis/Input/15VP-NFT/Output/Plots/MSI-af
terNFT-overlayed-plot.png", width = 8, height = 4, dpi = 300)

#*************************************************************************
#----------Paired-observation-plots----------------

#re-order factor levels for region
EEG$session <- factor(EEG$session, levels=c("before NFT", "after NFT"))
windowsFonts("Arial" = windowsFont("Arial"))

#Now I want to overlay mean individual connected lines for every VP and the mean plots for every two conditions to be mix
ed with the corresponding per-t-tests(source space) in one figure

simple<-EEG %>% filter(condition==c("simple"))

line3 <- ggplot( simple, aes(session, mu))

line3 +geom_hline(yintercept = 0, colour ="#bdbdbd")+stat_summary(fun = mean, geom = "line", aes(group = VP),color="#fec4
4f")+stat_summary(fun = mean, geom = "line", aes(group= index),color="black")+ stat_summary(fun.data = mean_cl_normal, ge
om = "pointrange")+ labs( x = "Session", y = "Mu Suppression Index (MSI)", colour = "session")+ theme_bw()+ theme(text =
element_text(size = 20, family = "Arial"))+geom_hline(yintercept = 0, colour ="#bdbdbd")
```

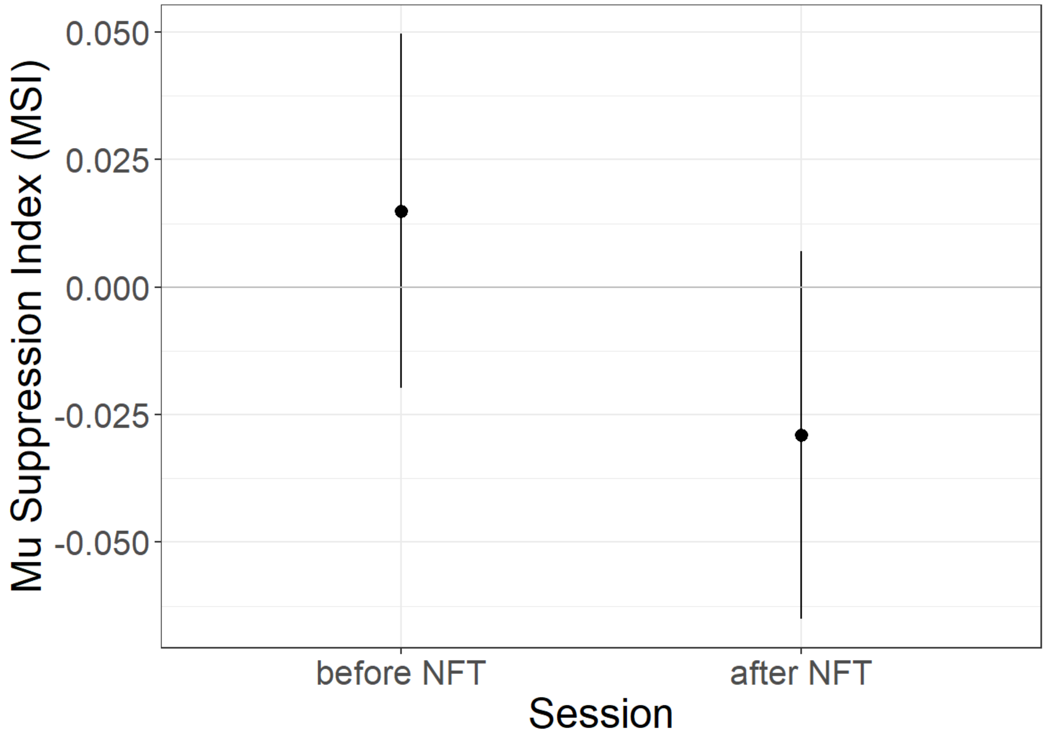


```
ggsave("C:/Users/dastg/Desktop/Main-Projects/Empra_2019_Dastgheib/Statistical_Analysis/Input/15VP-NFT/Output/Plots/simple
-Paired-observation.png", width = 8, height = 4.2, dpi = 300)

#***************************************

complex <-EEG %>% filter(condition==c("complex"))

line4 <- ggplot( complex, aes(session, mu))

line4 +geom_hline(yintercept = 0, colour ="#bdbdbd")+stat_summary(fun = mean, geom = "line", aes(group = VP),color="#fec4
4f")+stat_summary(fun = mean, geom = "line", aes(group= index),color="black")+ stat_summary(fun.data = mean_cl_normal, ge
om = "pointrange")+ labs( x = "Session", y = "Mu Suppression Index (MSI)", colour = "session")+ theme_bw()+ theme(text =
element_text(size = 20, family = "Arial"))+geom_hline(yintercept = 0, colour ="#bdbdbd")
```

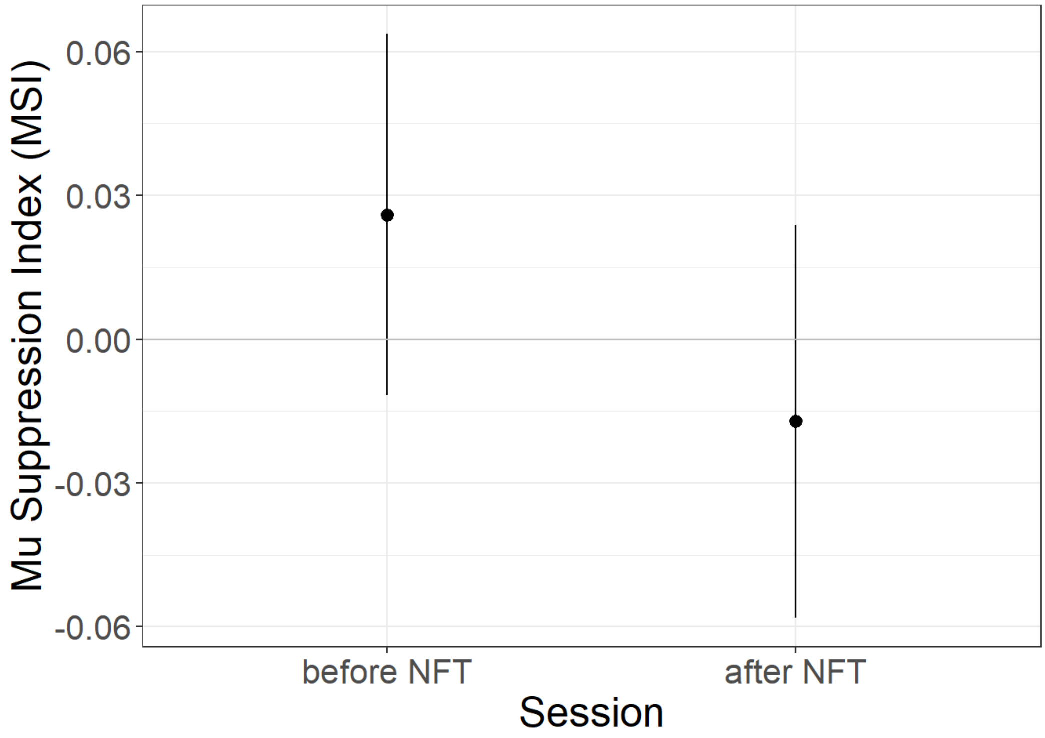

```
ggsave("C:/Users/dastg/Desktop/Main-Projects/Empra_2019_Dastgheib/Statistical_Analysis/Input/15VP-NFT/Output/Plots/comple
x-Paired-observation.png", width = 8, height = 4.2, dpi = 300)

#***************************************************************

social <-EEG %>% filter(condition==c("social"))

line5 <- ggplot(social, aes(session, mu))

line5 +geom_hline(yintercept = 0, colour ="#bdbdbd")+stat_summary(fun = mean, geom = "line", aes(group = VP),color="#fec4
4f")+stat_summary(fun = mean, geom = "line", aes(group= index),color="black")+ stat_summary(fun.data = mean_cl_normal, ge
om = "pointrange")+ labs( x = "Session", y = "Mu Suppression Index (MSI)", colour = "session")+ theme_bw()+ theme(text =
element_text(size = 20, family = "Arial"))+geom_hline(yintercept = 0, colour ="#bdbdbd")
```

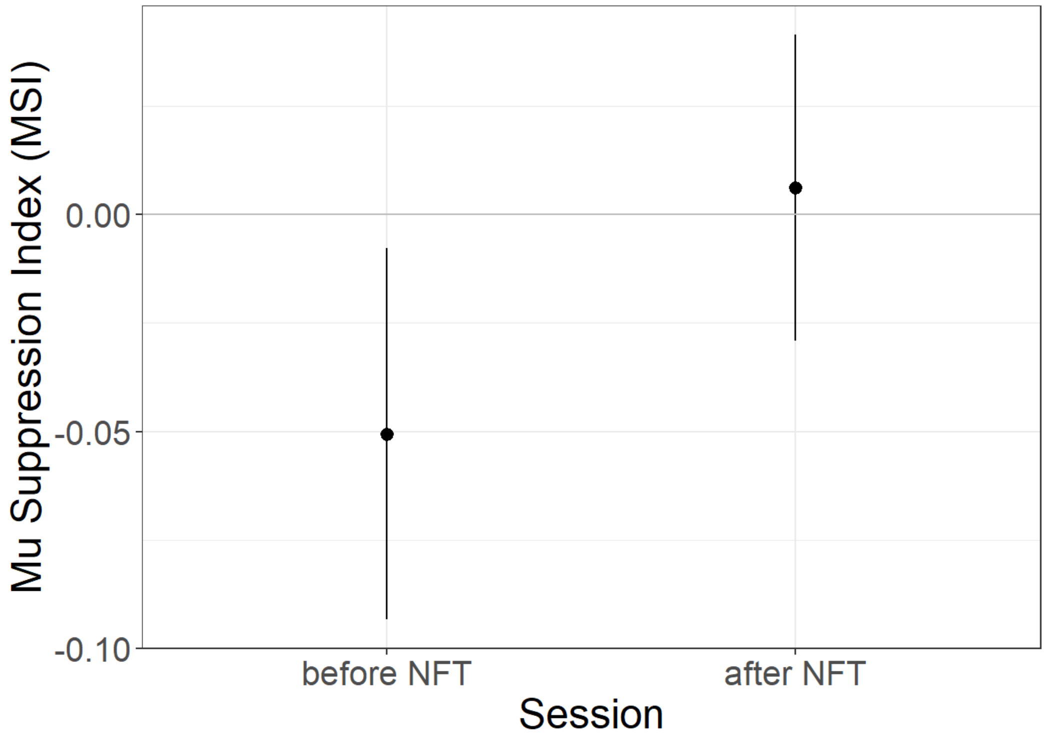


```
ggsave("C:/Users/dastg/Desktop/Main-Projects/Empra_2019_Dastgheib/Statistical_Analysis/Input/15VP-NFT/Output/Plots/Social
-Paired-observation.png", width = 8, height = 4.2, dpi = 300)
```

Neurofeedback data analysis In the following chuncks the data extracted from neurofeedback device are investigated. The data shows the information during the neurofeedback sessions in two states: The one minute baseline before each session which is called the Baseline, and the amplitud during the NFT training which is called feedback state.

```
NFT<-read.csv(file=("C:/Users/dastg/Desktop/Main-Projects/Empra_2019_Dastgheib/Statistical_Analysis/Input/15VP-NFT/Neurof
eedback.csv"),header = TRUE)

NFT[ ,c('VP', 'Date','Anzahl_Trials')] <- list(NULL);setnames(NFT, "VP_number", "VP")

#-----------------Creating a clean Data set-----------

#I want to average sessions that were divided
#VP3, Session 13a & 13b
#VP3, Session 14a & 14b
#VP6, Session 9a & 9 b
#VP7, Session 1a & 1b

VP3<-NFT%>% filter(Session=="1a" | Session== "1b"  )
AV<-NFT%>% filter(Session=="1a");AV$Session<-"1"
AV$MUE_C3_Baseline<-mean(VP3$MUE_C3_Baseline);AV$MUE_C3_Feedback<-mean(VP3$MUE_C3_Feedback);AV$MUE_C3.C4_Baseline<-mean(V
P3$MUE_C3.C4_Baseline);AV$MUE_C3.C4_Feedback<-mean(VP3$MUE_C3.C4_Feedback);AV$MUE_C4_Baseline<-mean(VP3$MUE_C4_Baseline);
AV$MUE_C4_Feedback<-mean(VP3$MUE_C4_Feedback);AV$THETA_C3.THETA_C4_baseline<-mean(VP3$THETA_C3.THETA_C4_baseline);AV$THET
A_C3.THETA_C4_feedback<-mean(VP3$THETA_C3.THETA_C4_feedback);AV$BETA_C3.BETA_C4_baseline<-mean(VP3$BETA_C3.BETA_C4_baseli
ne);AV$EMGLOW_C3.EMGLOW_C4_baseline<-mean(VP3$EMGLOW_C3.EMGLOW_C4_baseline);AV$EMGLOW_C3.EMGLOW_C4_feedback<-mean(VP3$EMG
LOW_C3.EMGLOW_C4_feedback)
NFT<-rbind(NFT,AV)

#The clean NFT database:

NFT<-NFT%>% filter(Session=="1" | Session== "2"|Session=="3" | Session== "4"|Session=="5" | Session== "6"|Session=="7" |
Session== "8"|Session=="9" | Session== "10"|Session=="11" | Session== "12"|Session=="13" | Session== "14"|Session=="15" )

#If you look at the data set, you would notice that participant number 7 has super high amplitude in almost all of the ba
nds in her first session. That is because she closed her eyes in that session! Thus we have to remove this session from o
ur data

NFTout<-subset(NFT, subset = VP== 7 & Session== 1)
NFT<-NFT[setdiff(rownames(NFT),rownames(NFTout)),]

write.csv(NFT,"C:/Users/dastg/Desktop/Main-Projects/Empra_2019_Dastgheib/Statistical_Analysis/Input/15VP-NFT/NFT_data.cs
v", row.names = FALSE)

#I want to group every 5 neurofeedback sessions so that we could compare data during NFT and see when the effects start t
o get significant. I add a value called Sgroup which means session group that has 3 levels: A (session1-5), B(Session 6-1
0), C(Session 11-15)

#newDataframe <- oldDataframe[rows, columns]

NFT<-read.csv(file=("C:/Users/dastg/Desktop/Main-Projects/Empra_2019_Dastgheib/Statistical_Analysis/Input/15VP-NFT/NFT_da
ta.csv"),header = TRUE)

NFT$Session <- as.numeric(NFT$Session)
NFTA<-NFT[NFT$Session<6,];NFTA$Sgroup<-"A"
NFTB<-NFT%>% filter(Session== "6"|Session=="7" | Session== "8"|Session=="9" | Session== "10");NFTB$Sgroup<-"B"
NFTC<-NFT[NFT$Session>10,];NFTC$Sgroup<-"C"
NFT<-rbind(NFTA,NFTB,NFTC)

#NFTF<-NFT[NFT$Session==1,];NFTF$twoG<-"first"
#NFTL<-NFT[NFT$Session==15,];NFTL$twoG<-"last"
#NFTA<- rbind(NFTF,NFTL)

write.csv(NFT,"C:/Users/dastg/Desktop/Main-Projects/Empra_2019_Dastgheib/Statistical_Analysis/Input/15VP-NFT/NFT_data.cs
v", row.names = FALSE)
```

Creating scatter plot of NFT data

```
#-------Creating scatter plot of NFT data --------------


NFT<-read.csv(file=("C:/Users/dastg/Desktop/Main-Projects/Empra_2019_Dastgheib/Statistical_Analysis/Input/15VP-NFT/NFT_data.csv"),header = TRUE)

attach(NFT)
as.factor( c(Session,MUE_C3_Baseline,MUE_C3_Feedback,MUE_C4_Baseline,MUE_C4_Feedback,MUE_C3.C4_Baseline,MUE_C3.C4_Feedback, BETA_C3.BETA_C4_baseline,BETA_C3.BETA_C4_feedback,THETA_C3.THETA_C4_baseline,THETA_C3.THETA_C4_feedback,EMGLOW_C3.EMGLOW_C4_baseline,EMGLOW_C3.EMGLOW_C4_feedback, Sgroup))
```

```
##    [1] 1     2     3     4     5     1     2     3     4     5     1     2    
##   [13] 3     4     5     1     2     3     4     5     1     2     3     4    
##   [25] 5     1     2     3     4     5     2     3     4     5     1     2    
##   [37] 3     4     5     1     2     3     4     5     1     2     3     4    
##   [49] 5     1     2     3     4     5     1     2     3     4     5     1    
##   [61] 2     3     4     5     1     2     3     4     5     1     2     3    
##   [73] 4     5     1     2     3     4     5     6     7     8     9     10   
##   [85] 6     7     8     9     10    6     7     8     9     10    6     7    
##   [97] 8     9     10    6     7     8     9     10    6     7     8     10   
##  [109] 6     7     8     9     10    6     7     8     9     10    6     7    
##  [121] 8     9     10    6     7     8     9     10    6     7     8     9    
##  [133] 10    6     7     8     9     10    6     7     8     9     10    6    
##  [145] 7     8     9     10    6     7     8     9     10    6     7     8    
##  [157] 9     10    11    12    13    14    15    11    12    13    14    15   
##  [169] 11    12    15    11    12    13    14    15    11    12    13    14   
##  [181] 15    11    12    13    14    15    11    12    13    14    15    11   
##  [193] 12    13    14    15    11    12    13    14    15    11    12    13   
##  [205] 14    15    11    12    13    14    15    11    12    13    14    15   
##  [217] 11    12    13    14    15    11    12    13    14    15    11    12   
##  [229] 13    14    15    11    12    13    14    15    8.97  9.31  6.83  7.03 
##  [241] 6.8   5.9   15.82 5.62  5.74  6.42  10.31 8.16  9.44  11.53 8.4   10.54
##  [253] 9.85  13.17 10.32 9.77  9.05  9.19  8.43  10.02 9.21  7.17  9.34  8.06 
##  [265] 7.29  7.87  9.38  7.73  9.16  9.21  10.85 9.02  14.56 10.16 9.07  9.65 
##  [277] 7.11  5.25  8.44  7.26  7.58  7.2   7.74  6.32  6.98  7.15  7.23  7.81 
##  [289] 9.64  9.17  4.72  4.92  4.1   4.46  4.69  6.68  7.38  7.66  7.84  7.63 
##  [301] 6.46  6.31  7.04  7.05  6.6   7.64  8.72  9.36  8.68  9.02  6.29  6.53 
##  [313] 6.03  6.68  6.66  8.18  10.11 9.87  7.87  8.37  6.26  7.11  5.86  5.25 
##  [325] 5.33  14.53 11.29 12.16 11.13 10.54 14.8  12.7  17.73 16.62 13.36 8.24 
##  [337] 7.49  10.05 8.95  8.06  8.53  8.86  10.49 7.75  10.47 9.49  10.38 9.89 
##  [349] 8.64  11.75 9.4   14.63 10.06 9.11  6.25  6.16  7.47  6.44  6.88  6.97 
##  [361] 9.8   8.48  9.1   7.6   7.49  7.92  9.44  8.32  8.74  4.57  4.69  4.08 
##  [373] 4.31  4.4   7.3   6.99  7.74  9.36  7.97  7     6.9   7.47  6.92  6.55 
##  [385] 9.16  8.24  8.38  8.57  8.97  6.98  6.59  7.7   5.98  6.76  8.96  10.35
##  [397] 8.32  10.25 9.77  5.2   9.8   7.66  6.32  5.56  14.57 11.07 14.11 13.12
##  [409] 15.08 10.86 11.08 12.57 8.34  8.69  8.21  9.91  9.03  8.94  8.48  9.16 
##  [421] 8.25  8.35  10    10.44 11.15 11.47 11.34 14.09 10.84 13.22 12    12.62
##  [433] 6.14  6.53  8.27  7.27  6.41  9.24  8.92  9.02  8.17  8.26  6.64  7.91 
##  [445] 8.03  6.49  6.59  4.06  4.5   3.99  4.14  4.52  9.22  6.99  6.89  7.6  
##  [457] 7.65  6.43  6.93  7.1   6.56  7.26  8.91  8.9   9.75  7.88  7.81  6.37 
##  [469] 6.3   6.32  6.26  7.14  9.52  8.22  8.1   7.9   8.58  5.29  6.24  5.03 
##  [481] 4.97  5.93  11.5  9.13  10.17 11.05 9.72  15.77 11.55 12.64 12.47 12.38
##  [493] 9.92  8.99  9.63  9.64  9.84  6.36  6.31  7.13  6.35  5.89  8.47  8.2  
##  [505] 9.68  9.08  14.02 11.5  16.35 11.92 9.74  9.5   7.73  6.83  9.16  7.32 
##  [517] 8.33  7.39  8.36  6.85  7.48  7.74  8.46  8.8   10.1  7.9   4.83  4.66 
##  [529] 4.53  4.19  4.59  7.44  7.88  8     8.03  7.26  6.36  6.19  6.24  6.49 
##  [541] 6.28  9.68  8.75  9.03  8.52  8.87  6.14  5.94  6.4   6.98  6.27  9.57 
##  [553] 9.43  9.21  8.93  9.63  5.62  4.86  5.3   5.18  4.89  11.35 10.56 11.25
##  [565] 10.9  10.19 13.98 11.69 16.18 16.07 13.9  9.23  9.79  9.8   9.61  8.69 
##  [577] 6.05  6.11  8.3   6.21  10.62 8.86  9.79  9.35  8.3   13.69 11.07 13.37
##  [589] 11.52 12.55 7.23  7.11  7.22  7.63  7.03  7.75  8.46  7.6   8.17  7.93 
##  [601] 8.58  9.07  9.3   9     8.78  4.36  4.21  4.43  4.52  4.45  8.17  7.22 
##  [613] 8.14  8.26  7.91  6.56  6.36  6.67  6.53  6.14  9.17  9.48  9.36  9.76 
##  [625] 9.28  6.39  6.43  6.75  5.89  6.22  9.52  8.75  8.95  8.81  9.16  5.17 
##  [637] 5.33  5.33  5.89  5.19  11.19 9.95  11.38 13.49 14.26 12.79 13.8  12.38
##  [649] 10.02 9.46  9.74  10.18 9.31  7.46  7.52  6.84  7.38  6.51  8.88  9.7  
##  [661] 9.78  10.1  10.05 11.65 11.61 13.69 11.86 11.93 6.32  5.75  6.92  7.09 
##  [673] 7.15  9.06  9.09  7.93  8.07  7.66  7.82  8.71  8.86  7.6   6.97  4.22 
##  [685] 4.49  4.16  4.61  4.38  8.28  6.79  7.1   7.19  7.58  6.45  6.55  6.68 
##  [697] 6.27  6.38  9.52  9.13  9.58  9.15  8.58  6.17  6.62  6.16  6.93  6.42 
##  [709] 8.07  8.55  6.69  7.56  6.59  5.96  5.37  5.04  4.95  5.57  9.93  8.28 
##  [721] 9.61  11.31 8.3   10.84 11.31 13.03 10.15 10.24 8.85  8.88  8.4   8.88 
##  [733] 8.51  7.61  13.43 8.78  7.52  8.84  8.89  7.36  7.86  8.33  19.18 9.38 
##  [745] 14.17 9.49  9.12  9.86  6.29  6.5   8.89  7.95  9.42  6.91  7.82  6.52 
##  [757] 7.02  6.55  8.35  7.89  10.44 8.64  5.47  4.97  4.34  4.44  4.25  8.87 
##  [769] 10.15 11.15 9.33  9.47  6.97  6     7.43  7.4   7.87  8.27  10.38 9.74 
##  [781] 9.74  9.06  6.63  6.34  7.01  6.3   6.63  7.13  8.47  7.84  7.33  7.11 
##  [793] 5.66  5.26  5.11  4.99  5.01  11.52 11.07 12.56 11.17 9.65  13.91 11.64
##  [805] 16.21 16.4  12.84 8.12  7.38  8.72  8.03  7.63  8.19  10.22 8.37  7.56 
##  [817] 8.28  9.15  9.59  8.54  8.33  11.35 10.8  13.28 11.22 10.38 6.99  5.81 
##  [829] 8.04  6.62  6.91  6.95  9.62  8.31  9.81  7.46  15.06 8.22  8.67  7.52 
##  [841] 7.72  4.33  4.53  4.64  4.67  4.27  7.71  7.93  9.49  11.56 9.43  6.85 
```

```
##  [853] 7.43  6.71  6.7   6.42  10.73 8.81  8.51  8.97  8.64  6.86  6.24  7.11
##  [865] 6.17  6.67  6.88  9.72  7.54  8.29  8.38  4.69  5.49  5.7   5.91  5.89
##  [877] 12.92 10.86 13.02 14.15 13.23 11.16 10.62 12.26 8.22  8     7.52  9.12
##  [889] 8.82  8.95  9.38  8.5   8.3   8.43  8.78  8.57  10.35 9.53  9.82  13.5
##  [901] 12.92 13.19 13.49 14.62 6.15  7.18  6.48  7.08  7.79  9.12  9.09  8.52
##  [913] 8.25  8.65  6.86  7.56  6.87  6.81  5.9   4.15  4.56  4.11  4.13  4.32
##  [925] 11.42 8.9   8.65  9.46  8.89  6.97  6.39  7.12  6.35  6.39  8.31  9.35
##  [937] 9.33  8.23  8.09  6.39  6.33  10.78 5.68  6.5   7.94  8.26  8.32  7.25
##  [949] 8.4   5.32  5     5.06  5.03  5.27  11.64 9.02  9.41  11.01 9.92  14.42
##  [961] 11.06 12.33 12.53 12.94 9.36  8.91  9.06  8.91  8.72  6.89  6.32  7.42
##  [973] 6.79  6.4   7.48  6.83  8.2   7.59  14.74 10.16 15.4  11.5  9.56  9.56
##  [985] 7.41  7.39  9.51  7.07  8.22  7.13  8.21  7.91  10.99 7.46  10.05 9.18
##  [997] 10.84 8.78  5.05  4.61  4.79  3.88  4.2   8.49  9.09  9.51  9.23  8.83
## [1009] 6.22  6.01  6.56  6.57  6.39  9.73  9.82  10.01 10.33 9.2   6.12  6.09
## [1021] 6.55  6.55  6.54  8.24  7.49  7.75  8.64  8.89  4.8   4.38  4.68  5
## [1033] 4.44  11.17 10.36 11.62 10.53 9.73  14.42 10.97 15.93 16.01 13.14 8.48
## [1045] 9.2   8.74  8.56  8.3   6.01  6.75  7.88  5.9   9.05  7.35  8.08  7.65
## [1057] 7.68  13.7  11.39 13.14 15.9  13.31 6.37  6.67  6.79  7.16  7.18  7.7
## [1069] 8.47  7.62  8.05  7.83  10.56 8.49  8.85  8.6   9.17  4.44  4.45  4.34
## [1081] 4.46  4.38  9.34  8.35  10.16 9.9   9.01  5.94  5.73  6.61  6.09  6.26
## [1093] 10.41 10.33 9.76  10.31 8.89  6.32  6.44  6.59  5.66  6.28  7.7   8.27
## [1105] 7.42  7.2   7.59  4.93  5.13  5.1   5.49  4.9   11.02 10.14 11.21 13.73
## [1117] 12.42 12.86 13.76 11.45 9.35  8.62  9.1   9.16  8.69  7.48  8.22  6.74
## [1129] 7.33  6.43  7.63  8.67  8.81  8.93  9.3   11.55 12.16 14.53 11.47 13.17
## [1141] 6.45  6.04  6.41  6.91  6.81  8.48  8.78  7.68  7.66  7.96  8.44  8.57
## [1153] 7.59  7.06  6.98  4.2   4.41  4.42  4.39  4.44  9.21  8.7   8.97  8.59
## [1165] 8.88  6.76  6.3   6.32  6.2   5.82  9.89  10.37 9.9   9.74  9.02  6.39
## [1177] 7.14  6.81  7.33  6.02  17.04 17.86 13.53 14.59 13.39 11.86 21.19 10.66
## [1189] 10.69 11.99 20.24 16.43 19.05 22.84 16.7  21.38 21.16 26.21 20.47 20.01
## [1201] 17.91 18.07 16.83 18.91 17.72 14.79 22.77 16.85 14.81 16.72 18.28 15.09
## [1213] 17.01 17.54 30.03 18.4  28.73 19.65 18.02 19.5  13.4  11.75 17.33 15.21
## [1225] 17    14.11 15.56 12.84 14    13.7  15.67 15.69 20.07 17.81 10.19 9.89
## [1237] 8.44  8.9   8.94  15.55 17.54 18.81 17.16 17.1  13.43 12.32 14.46 14.45
## [1249] 14.47 15.91 19.11 19.1  18.41 18.08 12.92 12.87 13.04 12.98 13.3  15.31
## [1261] 18.58 17.71 15.2  15.48 11.91 12.36 10.97 10.24 10.34 26.05 22.36 24.71
## [1273] 22.3  20.2  28.71 24.34 33.94 33.02 26.2  16.36 14.87 18.76 16.98 15.69
## [1285] 16.72 19.08 18.86 15.31 18.75 18.64 19.98 18.42 16.97 23.1  20.2  27.91
## [1297] 21.28 19.48 13.24 11.96 15.52 13.06 13.79 13.93 19.42 16.79 18.92 15.07
## [1309] 22.55 16.15 18.11 15.84 16.46 8.9   9.23  8.73  8.97  8.68  15.02 14.92
## [1321] 17.23 20.92 17.4  13.84 14.33 14.17 13.61 12.97 19.89 17.04 16.9  17.53
## [1333] 17.61 13.84 12.83 14.81 12.15 13.43 15.84 20.07 15.85 18.54 18.15 9.9
## [1345] 15.29 13.37 12.22 11.46 27.49 21.92 27.13 27.27 28.31 22.03 21.7  24.83
## [1357] 16.55 16.69 15.72 19.03 17.85 17.89 17.86 17.66 16.55 16.78 18.78 19.01
## [1369] 21.49 21    21.16 27.59 23.76 26.4  25.48 27.24 12.29 13.72 14.75 14.35
## [1381] 14.21 18.36 18.01 17.54 16.42 16.91 13.49 15.46 14.9  13.3  12.49 8.22
## [1393] 9.06  8.1   8.27  8.84  20.64 15.89 15.54 17.05 16.53 13.4  13.31 14.21
## [1405] 12.91 13.65 17.22 18.25 19.08 16.11 15.9  12.76 12.63 17.1  11.93 13.64
## [1417] 17.46 16.48 16.41 15.15 16.97 10.61 11.24 10.09 10    11.19 23.14 18.15
## [1429] 19.58 22.06 19.65 30.19 22.61 24.97 25    25.33 19.28 17.9  18.7  18.55
## [1441] 18.56 13.26 12.62 14.55 13.13 12.29 15.94 15.03 17.87 16.67 27.76 21.66
## [1453] 31.75 23.42 19.3  19.06 15.14 14.22 18.68 14.39 16.55 14.52 16.57 14.76
## [1465] 18.47 15.2  18.51 17.97 20.94 16.69 9.87  9.27  9.32  8.07  8.79  15.93
## [1477] 16.97 17.51 17.26 16.09 12.58 12.2  12.8  13.07 12.67 19.4  18.57 19.04
## [1489] 18.85 18.06 12.26 12.03 12.95 13.52 12.81 17.82 16.92 16.96 17.57 18.53
## [1501] 10.42 9.25  9.98  10.18 9.33  22.52 20.92 22.87 21.44 19.92 28.4  22.66
## [1513] 32.11 32.07 27.05 17.71 18.99 18.54 18.26 16.99 12.06 12.86 16.18 12.11
## [1525] 19.68 16.21 17.87 17    15.98 27.39 22.46 26.52 27.42 25.86 13.59 13.78
## [1537] 14.01 14.78 14.21 15.45 16.92 15.22 16.21 15.76 19.14 17.56 18.14 17.6
## [1549] 17.95 8.8   8.66  8.78  8.98  8.83  17.5  15.57 18.3  18.16 16.92 12.51
## [1561] 12.1  13.29 12.62 12.41 19.58 19.81 19.12 20.07 18.17 12.72 12.87 13.34
## [1573] 11.55 12.5  17.22 17.01 16.37 16.02 16.75 10.1  10.46 10.43 11.38 10.09
## [1585] 22.21 20.09 22.58 27.22 26.67 25.65 27.56 23.83 19.36 18.07 18.84 19.34
## [1597] 18    14.94 15.74 13.58 14.71 12.93 16.5  18.37 18.59 19.02 19.8  23.19
## [1609] 23.78 28.22 23.33 25.11 12.78 11.78 13.34 14    13.96 17.54 17.87 15.61
## [1621] 15.73 15.62 16.25 17.27 16.45 14.67 13.95 8.42  8.9   8.58  9     8.82
## [1633] 17.49 15.49 16.07 15.78 16.46 13.21 12.86 13    12.47 12.2  19.41 19.5
## [1645] 19.48 18.89 17.59 12.56 13.76 12.97 14.26 12.44 22.54 24.17 31.46 27.74
## [1657] 23.19 36.07 42    24.28 20.25 20.9  34.35 51.64 37.03 43.07 36.13 22.39
## [1669] 24.3  26.03 24.5  24.54 27.08 32.37 23.98 28.24 25.14 28.2  58.02 24.79
## [1681] 24.96 27.96 37.28 31.94 33.66 34.13 53.03 27.75 31.55 27.94 29.04 26.91
## [1693] 27.15 26.14 29.78 37.13 43.95 25.09 28.65 27.68 27.15 19.23 18.55 20.87
## [1705] 20.42 21.94 33.03 31.96 27.59 27.03 19.02 21.06 21.58 23.32 25.47 21.8
```

```
## [1717] 35.22 29.96 36.94 36.05 36.97 28.33 33.29 36.43 35.61 29.36 28.28 40.94
## [1729] 25.9  35.32 40.52 22.37 26.92 21    23.14 21.06 25.39 23.77 20.3  20.25
## [1741] 22.84 26.9  45.7  29.81 40.23 44.09 33.16 29.9  28.36 27.84 28.29 24.37
## [1753] 25.35 26.52 24.07 23.73 24.84 38.06 31.06 22.53 38.68 38.05 40.08 40.48
## [1765] 34.51 30.61 36.69 29.94 30.19 33.92 34.15 25.67 33.29 31.45 31.17 31.17
## [1777] 31.92 31.35 50.58 28.32 26.09 22.19 19.96 19.64 20.54 21.09 22.66 21.71
## [1789] 22.29 20.27 22.64 24.12 22.27 23.5  23.43 34.89 38.5  38.21 31.48 32.23
## [1801] 38.88 27.79 25.11 31.44 27.95 43.11 29.55 35.04 28.01 28.51 26.78 41.29
## [1813] 22.18 39.54 27.69 21.48 23.61 21.53 19.99 20.32 63.96 67.73 39.85 32.23
## [1825] 33.3  26.98 24.4  24.71 23.45 23.69 21.94 26.25 24.05 28.99 24.45 29.98
## [1837] 25.8  33.7  37.73 34    41.6  36.73 35.76 33.34 26.22 28.28 31.19 29.44
## [1849] 23.83 25.58 27.46 22.82 29    33.11 40.63 29.74 33.55 40.69 19.03 19.75
## [1861] 20.24 27.18 18.69 21.22 25.12 16.73 20.62 24.18 22.4  21.24 19.58 21.56
## [1873] 23.1  33.27 28.8  36.58 29.47 35.16 26.76 29.82 28.63 25.03 25.42 25.6
## [1885] 32.97 32.55 40.48 28.68 22.48 20.11 19.75 23.18 20.22 22.28 20.85 19.25
## [1897] 18.72 20    35.26 30.15 26.42 30.45 27.93 24.45 22.35 22.88 22.44 23.45
## [1909] 27.05 25.49 25.11 25.31 24.85 21.82 22.54 25.01 22.34 22.61 31.76 31.18
## [1921] 33.97 30.66 32.3  29.53 33.95 29.98 29.15 30.06 27.06 25.66 27.16 23.85
## [1933] 29.06 26.3  29.13 29.9  35.95 20.36 20.92 20.69 21.61 20.4  28.02 25.65
## [1945] 22.1  18.92 19.2  21.22 22.59 21.88 21.34 20.57 25.78 24.48 25.94 27.89
## [1957] 24.3  33.75 28.53 34.68 40.72 30.31 24.41 22.62 23.55 23.28 23.78 21.57
## [1969] 22.01 20.12 21.84 20.9  19    17.91 18.95 18.94 18.32 29.17 30.62 28.44
## [1981] 26.51 26.15 25.11 24.12 26.51 25.66 24.36 23.99 26.71 25.8  24.88 24.11
## [1993] 20.99 22.72 23.18 21.22 36.35 31.77 34.3  33.69 30.31 30.91 29.74 29.78
## [2005] 40.2  30.37 24.39 25.3  24.87 26.07 25.54 30.89 29.25 28.48 28.88 26.78
## [2017] 21.04 22.53 21.4  21.39 21.81 18.63 19.42 20.16 22.69 21.41 21.75 20.66
## [2029] 22.6  21.85 22.07 26.02 22.35 27.1  23.73 24.99 33.59 32.27 28.34 33.28
## [2041] 28.54 22.75 22.85 23.15 22.62 23.66 23.41 35.59 22.91 22.71 21.39 18.45
## [2053] 18.69 19.15 18.77 18.46 28.75 31.11 30.01 24.51 26.12 23.73 23.49 25.3
## [2065] 24.33 24.16 24.54 26.43 24.93 21.97 23.74 21.84 23.08 21.22 33.14 34.49
## [2077] 35.03 35.47 36.75 28.45 28.73 30.74 28.48 30.87 24.98 23.3  23.89 24.34
## [2089] 24.1  28.87 30.92 26.65 28.72 27.31 21.1  21.45 21.61 21.08 21.13 20.72
## [2101] 20.68 19.63 20.34 20.43 22.15 20.28 20.64 20.4  21.09 26.43 24.21 24.7
## [2113] 23.44 22.74 32.66 32.35 31.48 19.39 28.48 23.71 25.21 26.09 25.33 22.39
## [2125] 10.43 11.24 12.51 9.28  8.67  11.87 20.77 9.69  8.93  11.61 14.93 12.75
## [2137] 12.49 14.8  12.67 17.57 22.56 19.06 18.92 16    17.56 17.56 17.26 16.68
## [2149] 15.9  14.96 19.17 18.44 18.09 20.24 15.87 13.07 13.81 14.96 24.87 14.86
## [2161] 22.88 16.08 14.27 14.6  14.25 10.4  12.6  15.27 13.99 15.92 12.25 10.33
## [2173] 12.89 9.48  10.75 10    11.72 12.98 9.46  11.28 9.4   10.39 9.06  11.61
## [2185] 12.29 11.57 13.44 11.07 15.82 15.34 15.52 15.9  16.08 14.57 14.3  14.22
## [2197] 14.16 15.08 13.93 16.14 15.13 17.94 13.38 9.56  10.67 10.36 9.95  9.08
## [2209] 11.27 12.97 9.8   8.51  9.32  14.85 17.39 15.24 14.85 14.12 20.27 18.39
## [2221] 18.96 20.41 18.79 16.54 16.02 17.14 17.9  16.47 18.35 21.65 20.56 18.07
## [2233] 16.1  14.29 15.99 13.63 12.73 16.07 16.42 17.17 15.72 15.24 13.43 10.59
## [2245] 13.5  10.65 12.02 12.76 13.97 11.1  12.98 13.79 19.33 12.01 10.44 10.47
## [2257] 10.92 8.93  9.6   9.76  8.89  8.81  12.27 13.85 12.62 12.72 12.25 15.5
## [2269] 16.65 17.99 17.23 16.83 14.43 12.32 13.89 13.75 14.12 14.54 18.18 24.41
## [2281] 13.78 19.85 10.4  11.35 10.13 10.3  10.83 8.19  15.53 14.15 10.69 11.14
## [2293] 18.23 14.21 16.65 21.15 22.1  17.68 14.78 24.33 15.58 15.57 14.01 15.33
## [2305] 14.71 18.56 18.79 19.84 20.68 22.35 13.53 13.64 14.82 14.57 15.11 15.25
## [2317] 13.56 16.64 14.73 16    11.54 12.34 13.33 11.7  10.3  13.22 12.79 11.91
## [2329] 15.74 14.78 10.07 9.61  11.03 10.42 10.39 8.5   9.7   8.39  9.21  9.51
## [2341] 14.48 11.06 11.27 10.86 12.39 16.3  16.74 17.78 15.92 16.15 13.2  13.86
## [2353] 15.54 12.3  14.36 18.72 13.6  30.28 12.96 19.86 10.85 10.54 9.89  10.2
## [2365] 9.98  10.52 10.11 8.52  7.94  9.76  16.51 14.58 14    14.78 14.86 20.68
## [2377] 22.31 18.34 19.46 18.01 16.72 15.24 17.7  15.78 17.17 12.61 13.37 14.48
## [2389] 14.33 14.7  13.69 13.17 13.86 13.46 17.45 16.33 21.48 18.19 15.65 14.69
## [2401] 12.74 11.06 12.14 10.32 11.65 12.52 11.83 12.45 14.21 9.73  11.15 10.8
## [2413] 11.3  11.31 9.8   9.33  9.38  8.37  9.19  12.71 13.51 13.3  12.72 12.89
## [2425] 15.27 15.51 15.05 15.01 15.07 15.63 14.15 14.76 14.31 14.61 12.42 12.75
## [2437] 13.4  13.15 13.76 10.84 10.59 10.4  10.29 10.83 8.64  8.47  8.29  8.49
## [2449] 8.27  16.2  14.9  15.13 14.47 14.45 19.9  18.35 20.34 22.23 19.84 15.5
## [2461] 18.16 17.1  17.66 16.17 13.32 13.77 16.43 13.52 15.24 13.18 14.36 13.15
## [2473] 12.78 18.21 16.65 17.6  19.43 16.01 10.2  11.48 10.97 12    11    12.19
## [2485] 11.52 11.39 10.81 11.2  11.8  12.48 11.48 11.45 11.63 9.13  9.12  9.14
## [2497] 9.28  9.19  13.61 12.96 13.05 12.83 13    15.1  15.69 16.14 16.08 15.61
## [2509] 14.58 14.85 15.24 16.28 15.39 13.33 19.51 14.74 12.05 12.98 11.09 11.36
## [2521] 10.48 10.46 11.07 8.59  8.52  8.74  10.23 8.43  15.16 14.32 16.45 20.49
## [2533] 19.29 22.07 20.88 18.92 16.51 15.71 16    17.44 15.24 14.41 15.11 14.07
## [2545] 15.01 13.53 12.93 14.44 14.27 15.18 15.98 14.97 15.57 15.54 15.48 16.43
## [2557] 11.21 10.16 11.46 11.94 10.9  11.5  11.84 10.69 12.32 13.27 11.04 11.32
## [2569] 10.84 10.64 10.47 8.74  9.14  9.01  9.3   9.07  13.18 12.48 12.75 12.2
```

```
## [2581] 12.95 15.81 16.31 15.69 15.44 15.09 15.34 15.14 15.32 14.88 15.2  12.98
## [2593] 13.29 17.04 12.4  12.73 23.3  26.33 38.56 31.58 25.79 42.68 45.65 26.73
## [2605] 20.43 21.2  39.02 65.97 43.53 51.48 43.94 23.16 25.04 27.45 24.48 26.6
## [2617] 28.09 36.87 25.11 30.56 25.89 29.29 66.37 23.71 25.56 29.01 33.12 27.9
## [2629] 28.53 30.67 53.31 25.12 24.51 25.49 25.81 23.8  30.16 27.59 31.94 42.21
## [2641] 52.63 24.98 28.33 29.36 28.13 19.39 18.15 21.47 19.38 21.58 43.89 42.69
## [2653] 35.3  33.93 21.76 23.1  21.76 23.05 27.21 22.6  39.31 34.8  43.89 44.59
## [2665] 43.52 25.42 33.85 35.27 36.69 27.39 33.09 51.65 29.74 43.05 50.49 24.11
## [2677] 28.92 21.93 24.97 22.57 27.42 23.38 20.33 21.08 25.71 28.31 54.61 32.03
## [2689] 47.61 53.43 37.39 32.94 28.41 28.83 30.5  25.73 26.99 27.53 24.87 24.36
## [2701] 23.71 36.97 34.19 23.28 36.16 33.92 36.74 38.31 30.63 26.66 35.25 24.17
## [2713] 27.76 33.58 41.49 26.27 38.63 37.05 35.41 30.77 31.61 29.7  61.26 29.97
## [2725] 25.88 22.32 18.63 18.96 19.66 26.06 29.42 26.54 27.58 24.42 23.73 24.97
## [2737] 22.23 23.52 24.29 43.46 47.09 44.15 35.51 37.36 38.35 25.78 21.82 30.6
## [2749] 25.25 54.64 35.04 42.91 32.78 32.53 30.14 52.88 23.54 48.52 31.04 23.41
## [2761] 22.53 20.34 19.84 21.13 79.91 86    42.98 36.65 36.58 29.73 25.56 25.63
## [2773] 24.05 24.4  22.21 26.75 25.17 29.29 23.18 30.56 24.54 37.89 36.04 27.76
## [2785] 38.89 33.16 28.47 29.47 23.05 24.63 29.21 26.12 25.78 27.51 27.63 25.33
## [2797] 30.52 33.71 44.46 28.22 34.51 45.66 19.09 19.33 20.04 32.13 19.23 26.39
## [2809] 32.74 18.91 24.99 31.39 21.44 23.15 19.68 22.94 24.39 38.04 32.33 44.43
## [2821] 35.46 41.17 23.82 27.96 26.27 22.45 23.92 28.21 38.64 39.65 50.31 33.49
## [2833] 23.45 20.71 20.7  25.53 20.97 23.55 21.08 19.67 18.84 19.58 35.99 33.82
## [2845] 27.18 33.29 30.07 24.91 23.27 23.25 22.46 23.75 27.27 25.79 25.55 26.01
## [2857] 25.06 21.2  22.43 25.33 22.02 22.11 28.88 28.08 31.14 27.52 24.12 24.73
## [2869] 25.94 24.9  24.97 28.71 28.37 23.79 25.18 22.63 28.69 25.86 27.42 31.56
## [2881] 37.68 20.37 20.39 19.86 19.83 20.55 35.52 31.61 26.07 22.24 22.18 21.28
## [2893] 24.02 21.27 21.08 20.49 27.49 26.3  27.16 30.61 25.09 30.04 25.08 32.76
## [2905] 41.73 27.68 26.62 24.25 25.18 24.34 25.22 22.41 23.19 20.55 22.58 20.99
## [2917] 18.76 18.07 19.13 18.74 18.96 28.91 33.56 27.92 27.07 25.39 25.35 25.31
## [2929] 26.51 25.72 24.81 24.88 26.83 25.66 25.39 23.97 20.87 20.95 21.64 21.86
## [2941] 31.34 29.13 29.85 29.63 27.42 24.08 24.17 22.69 39.64 24.31 25.37 23.47
## [2953] 25.71 25.89 25.26 30.29 27.13 26.82 26.79 26.13 20.44 22.25 19.68 19.79
## [2965] 19.76 21.21 22.61 23.76 27.59 25.56 21.45 20.5  21.77 20.77 21.47 29.44
## [2977] 22.88 28.75 24.39 26.56 29.97 28.17 23.28 30.31 24.5  23.85 24.71 24.54
## [2989] 24.1  24.44 24.57 45.02 24.84 24.15 22.17 18.54 18.89 19.48 18.04 18.86
## [3001] 28.35 33.57 27.54 24.7  26.96 24.01 23.41 26.88 24.02 24.99 24.23 26.34
## [3013] 25.53 20.42 22.07 21.48 21.65 20.52 31.26 20.33 32.3  32.78 32.07 24.73
## [3025] 23.01 26.18 24.01 25.93 24.92 22.72 22.39 22.51 22.35 25.32 27.04 24.77
## [3037] 26.03 25.27 20.28 19.7  20.64 21.14 22.13 25.05 24.28 23.03 24.03 24.3
## [3049] 21.38 20.4  20.97 20.81 21.08 27.79 24.41 24.96 23.83 23.39 29.34 29.69
## [3061] 27.1  25.44 26.17 25.25 26.33 29.39 26.45 23.54 A     A     A     A
## [3073] A     A     A     A     A     A     A     A     A     A     A     A
## [3085] A     A     A     A     A     A     A     A     A     A     A     A
## [3097] A     A     A     A     A     A     A     A     A     A     A     A
## [3109] A     A     A     A     A     A     A     A     A     A     A     A
## [3121] A     A     A     A     A     A     A     A     A     A     A     A
## [3133] A     A     A     A     A     A     A     A     A     A     A     A
## [3145] A     A     A     B     B     B     B     B     B     B     B     B
## [3157] B     B     B     B     B     B     B     B     B     B     B     B
## [3169] B     B     B     B     B     B     B     B     B     B     B     B
## [3181] B     B     B     B     B     B     B     B     B     B     B     B
## [3193] B     B     B     B     B     B     B     B     B     B     B     B
## [3205] B     B     B     B     B     B     B     B     B     B     B     B
## [3217] B     B     B     B     B     B     B     B     B     B     C     C
## [3229] C     C     C     C     C     C     C     C     C     C     C     C
## [3241] C     C     C     C     C     C     C     C     C     C     C     C
## [3253] C     C     C     C     C     C     C     C     C     C     C     C
## [3265] C     C     C     C     C     C     C     C     C     C     C     C
## [3277] C     C     C     C     C     C     C     C     C     C     C     C
## [3289] C     C     C     C     C     C     C     C     C     C     C     C
## [3301] C     C     C     C
## 1807 Levels: 1 10 10.01 10.02 10.05 10.06 10.07 10.09 10.1 10.11 10.13 ... C
```

```
scatterNFT1 <- ggplot(NFT,aes(Session, MUE_C3_Baseline,))
scatterNFT1+ scale_y_continuous(limits=c(2, 18))+ geom_point() + labs(x = "Session", y = "Mu Voltage at C3-Baseline")+ ge
om_smooth(method = "lm", colour = "Red")
```

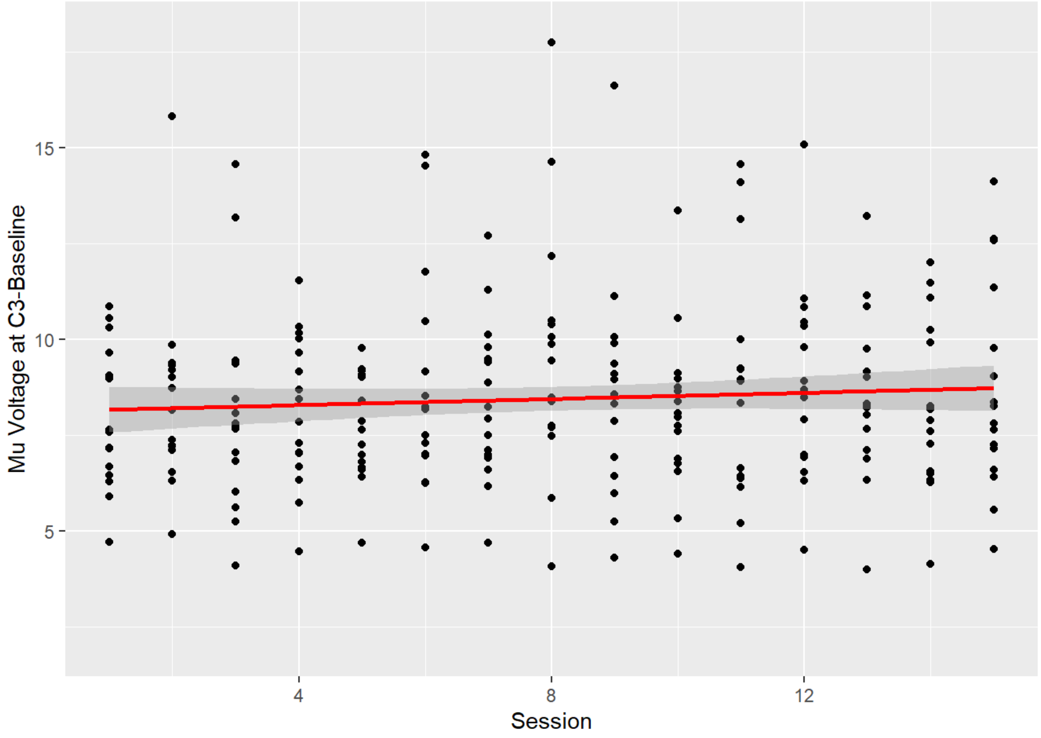


```
ggsave("C:/Users/dastg/Desktop/Main-Projects/Empra_2019_Dastgheib/Statistical_Analysis/Input/15VP-NFT/Output/Plots/scatte
rNFT-Mu-C3-F.png", width = 8, height = 4, dpi = 300)

scatterNFT2 <- ggplot(NFT,aes(Session, MUE_C4_Baseline))
scatterNFT2 + geom_point() + labs(x = "Session", y = "Mu Voltage at C4-Baseline")+ geom_smooth(method = "lm", colour = "R
ed")
```

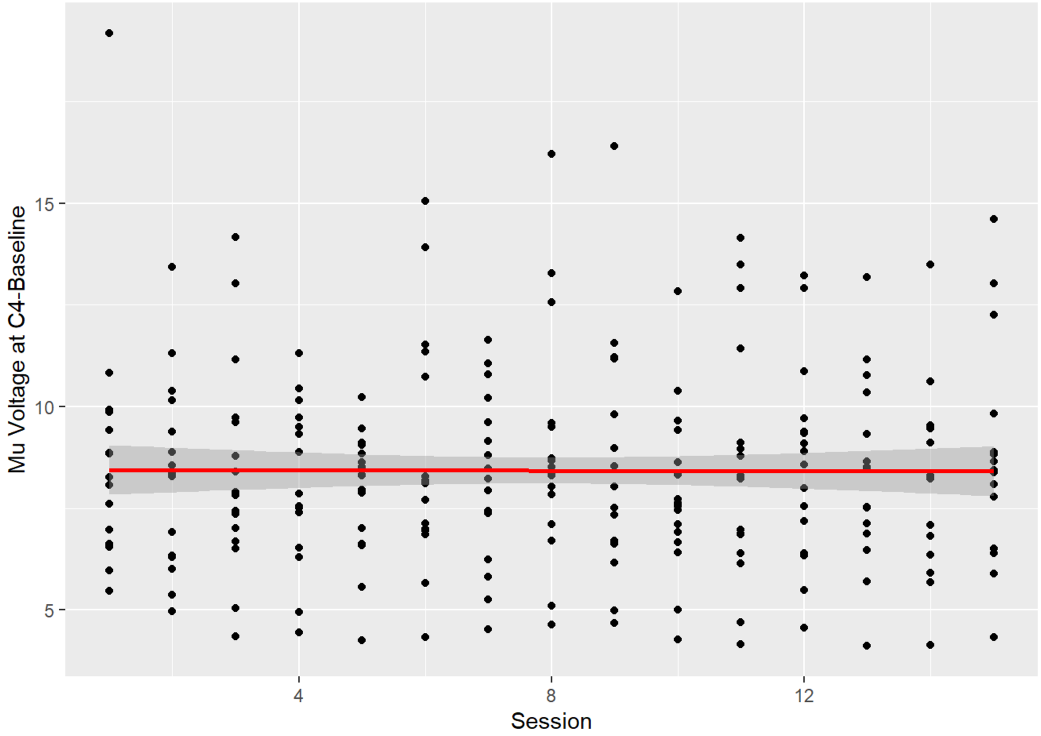


```
scatterNFT3 <- ggplot(NFT,aes(Session, MUE_C3_Feedback))
scatterNFT3 + geom_point() + labs(x = "Session", y = "Mu Voltage at C3-Feedback")+ geom_smooth(method = "lm", colour = "R
ed")
```

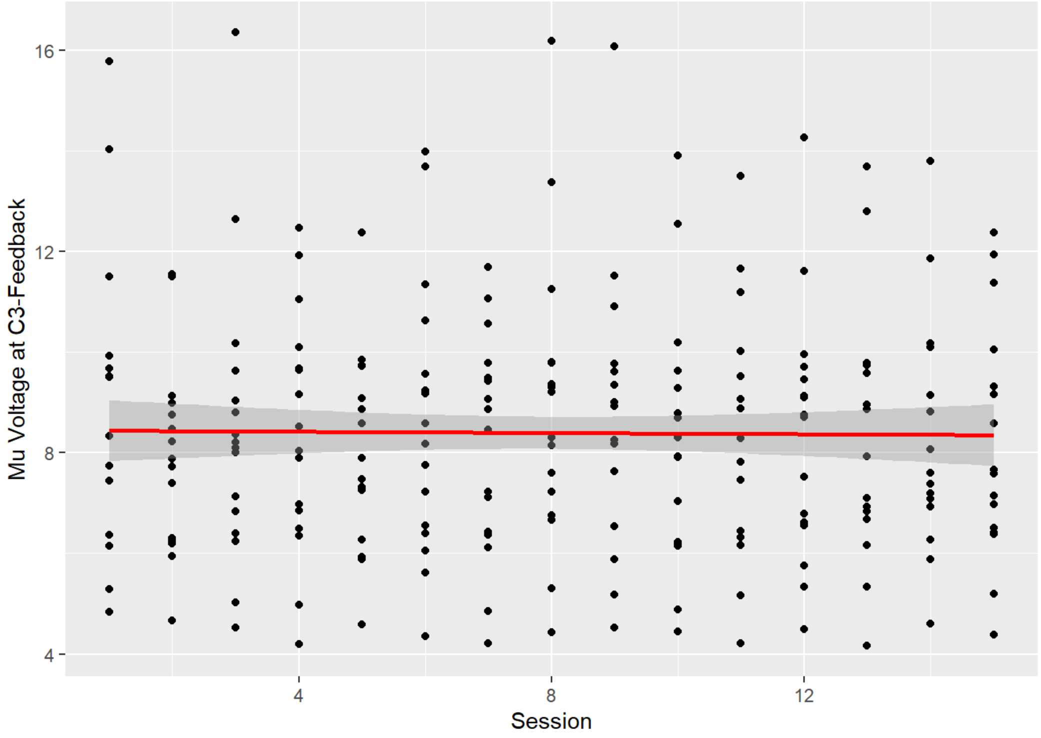


```
scatterNFT4 <- ggplot(NFT,aes(Session, MUE_C4_Feedback))
scatterNFT4 + geom_point() + labs(x = "Session", y = "Mu Voltage at C4-Feedback")+ geom_smooth(method = "lm", colour = "R
ed")
```

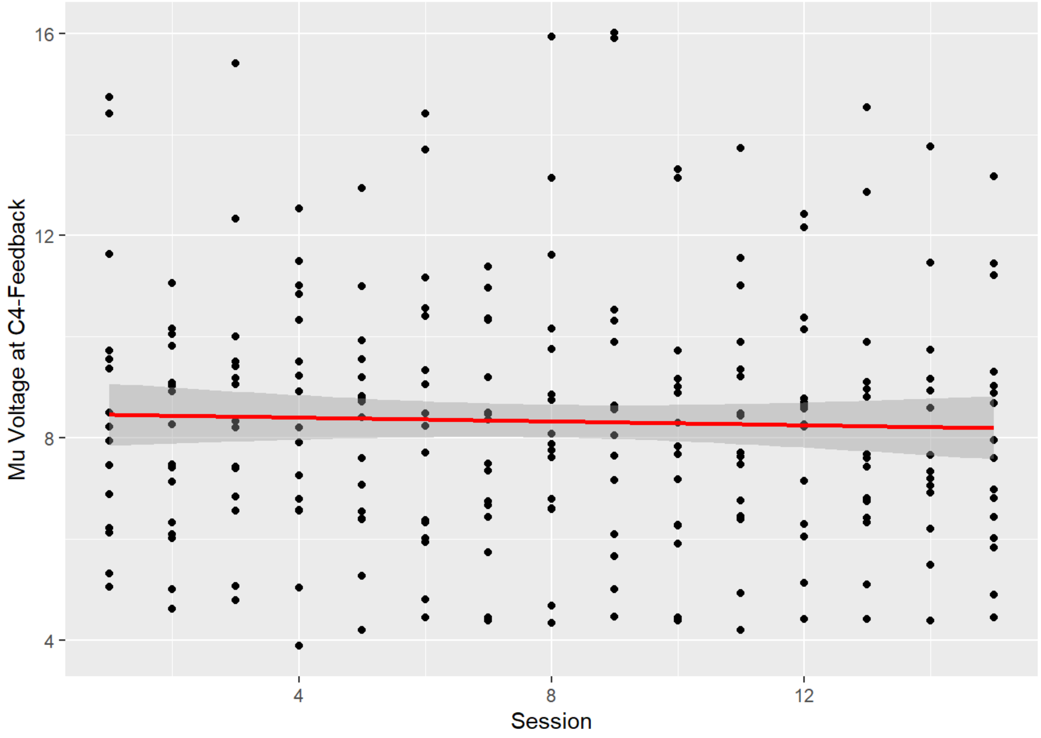


```
#


scatterNFT5 <- ggplot(NFT,aes(Session, MUE_C3.C4_Baseline))
windowsFonts("Arial" = windowsFont("Arial"))
scatterNFT5 + geom_point(color="#fec44f")+ labs(x = "Session", y = "Mu amplitude C3-C4 montage (µV)")+ geom_smooth(method
= "lm", colour = "Black") + scale_x_continuous(name="Sessions", limits=c(1, 10), breaks = c(1,2,3,4,5,6,7,8,9,10)) + them
e_bw()
```

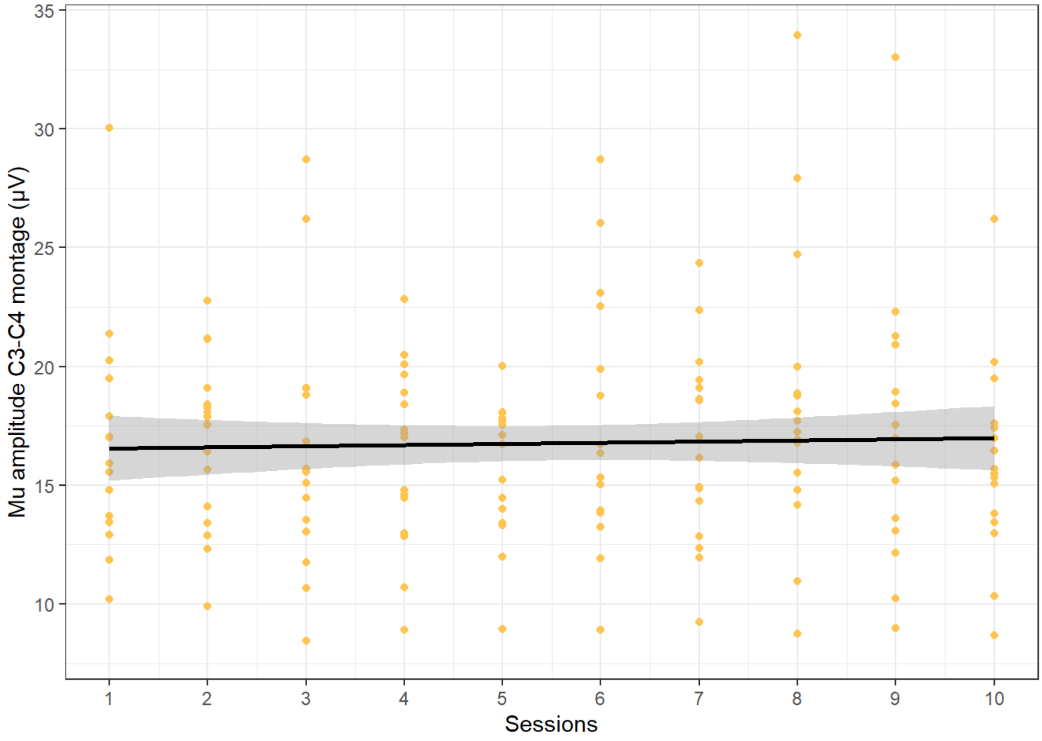


```
ggplot(NFT, aes(Session, MUE_C3.C4_Baseline))+ geom_point(aes(y= MUE_C3.C4_Baseline),color="#fec44f",position =position_dodge(width = 0.5))+ stat_summary(fun = mean, geom = "line") + stat_summary(fun = mean, geom = "point", aes(group = Session))+ labs(x = "Session", y = "Mu amplitude C3-C4 montage (µV)")+ scale_x_continuous(name="Sessions", limits=c(1, 10), breaks = c(1,2,3,4,5,6,7,8,9,10)) + theme_bw()+ theme(text = element_text(size = 15, family = "Arial"))
```

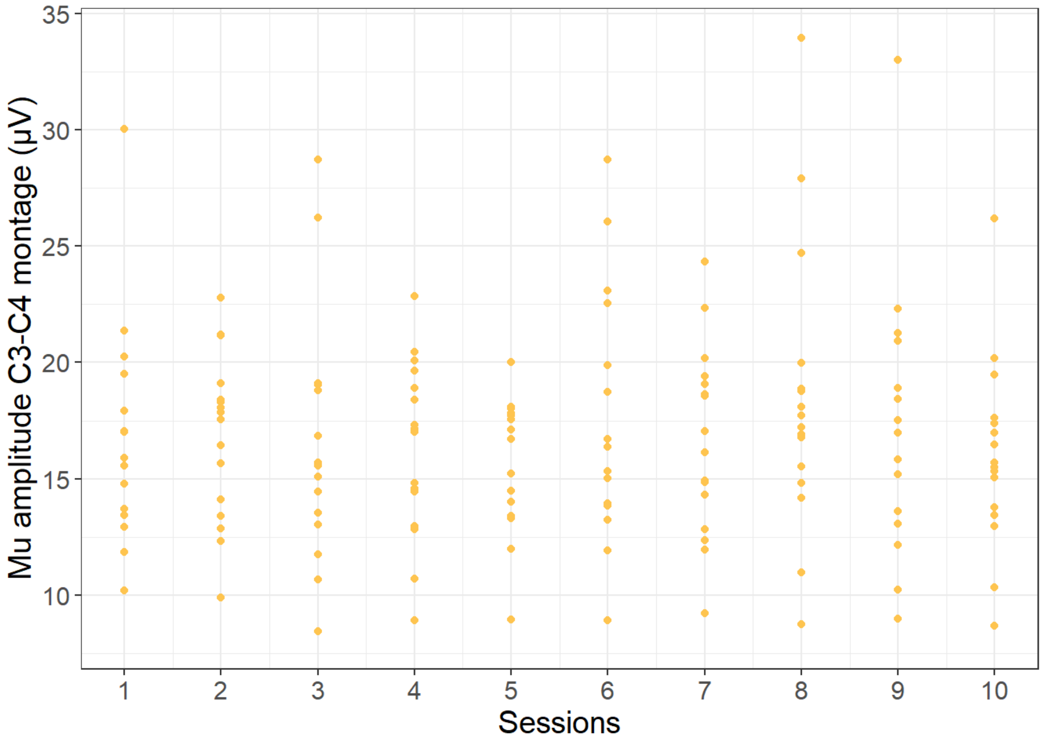


```
ggsave("C:/Users/dastg/Desktop/Main-Projects/Empra_2019_Dastgheib/Statistical_Analysis/Input/15VP-NFT/Output/Plots/-Scatter-NFT-Mu-C3C4-baseline.png", width = 8, height = 4, dpi = 300)

scatterNFT6 <- ggplot(NFT,aes(Session, MUE_C3.C4_Feedback))
windowsFonts("Arial" = windowsFont("Arial"))
scatterNFT6 + geom_point(color="#fec44f")+ labs(x = "Session", y = "Mu amplitude C3-C4 montage (µV)")+ geom_smooth(method = "lm", colour = "Black") + scale_x_continuous(name="Sessions", limits=c(1, 10), breaks = c(1,2,3,4,5,6,7,8,9,10)) + theme_bw()
```

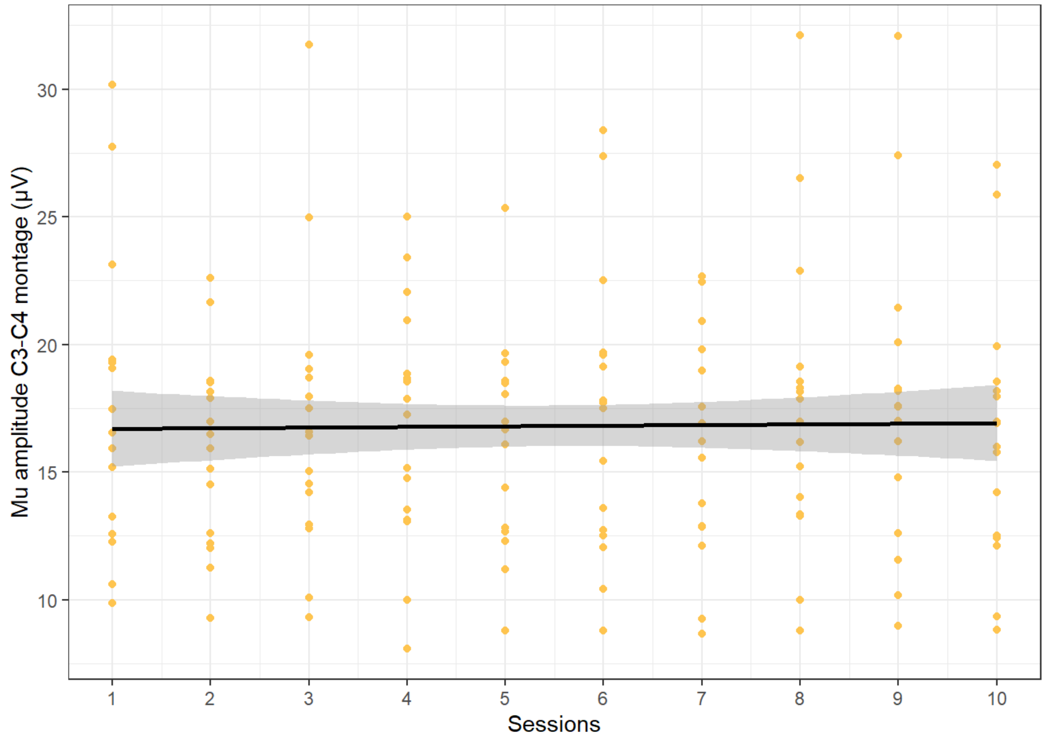


```
ggplot(NFT, aes(Session, MUE_C3.C4_Feedback))+ geom_point(aes(y= MUE_C3.C4_Feedback),color="#fec44f",position =position_d
odge(width = 0.5))+ stat_summary(fun = mean, geom = "line") + stat_summary(fun = mean, geom = "point", aes(group = Sessio
n))+ labs(x = "Session", y = "Mu amplitude C3-C4 montage (µV)")+ scale_x_continuous(name="Sessions", limits=c(1, 10), bre
aks = c(1,2,3,4,5,6,7,8,9,10)) + theme_bw()+ theme(text = element_text(size = 15, family = "Arial"))
```

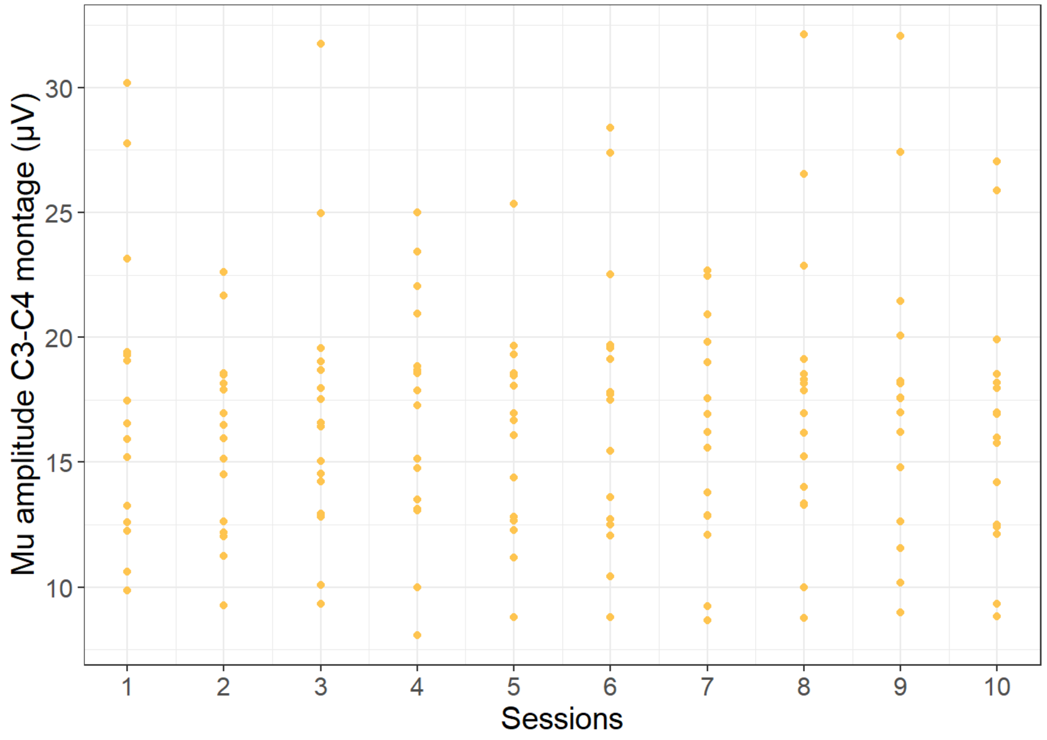


```
ggsave("C:/Users/dastg/Desktop/Main-Projects/Empra_2019_Dastgheib/Statistical_Analysis/Input/15VP-NFT/Output/Plots/-Scatt
er-NFT-Mu-C3C4-feedback.png", width = 8, height = 4, dpi = 300)

scatterNFT7 <- ggplot(NFT,aes(Session, BETA_C3.BETA_C4_baseline))
scatterNFT7 + geom_point() + labs(x = "Session", y = "Beta Voltage at C3.C4-Baseline")+ geom_smooth(method = "lm", colour
= "Red")
```

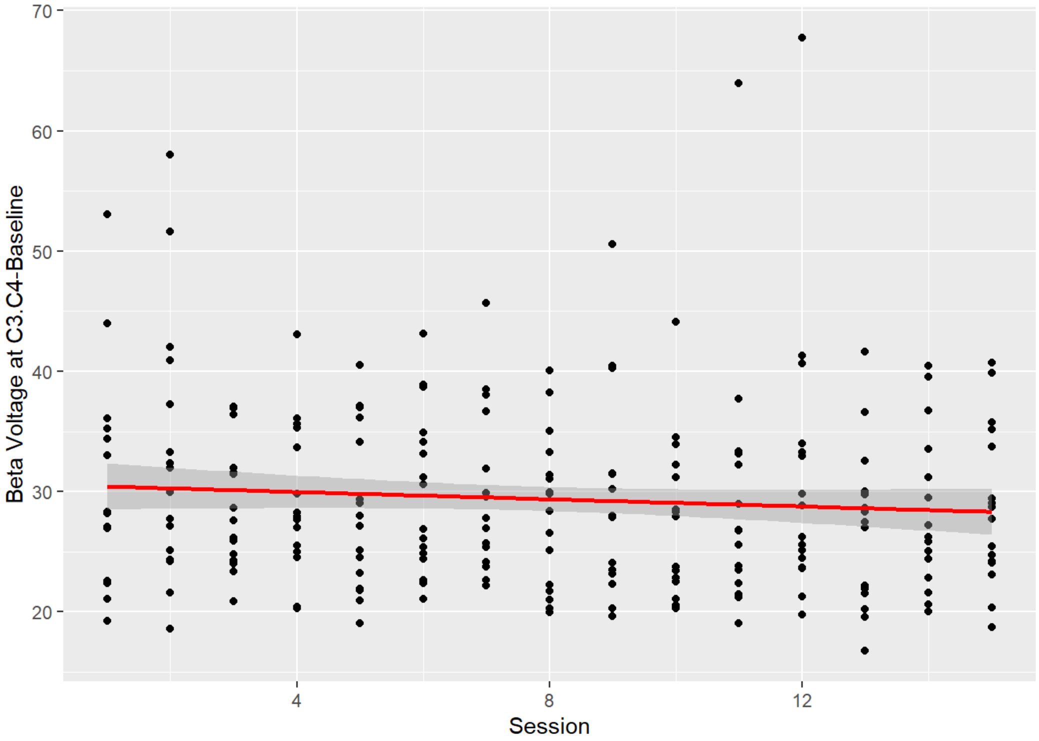


```
ggplot(NFT, aes(Session, MUE_C3.C4_Feedback))+ geom_point(aes(y= MUE_C3.C4_Feedback),color="#fec44f",position =position_dodge(width = 0.5))+ stat_summary(fun = mean, geom = "line") + stat_summary(fun = mean, geom = "point", aes(group = Session))+ labs(x = "Session", y = "Mu amplitude C3-C4 montage (µV)")+ scale_x_continuous(name="Sessions", limits=c(1, 15), breaks = c(1,2,3,4,5,6,7,8,9,10,11,12,13,14,15)) + theme_bw()+ theme(text = element_text(size = 15, family = "Arial"))+stat_summary(fun.data = mean_cl_normal, geom = "pointrange")+ geom_smooth(method = "lm", colour = "Red")
```

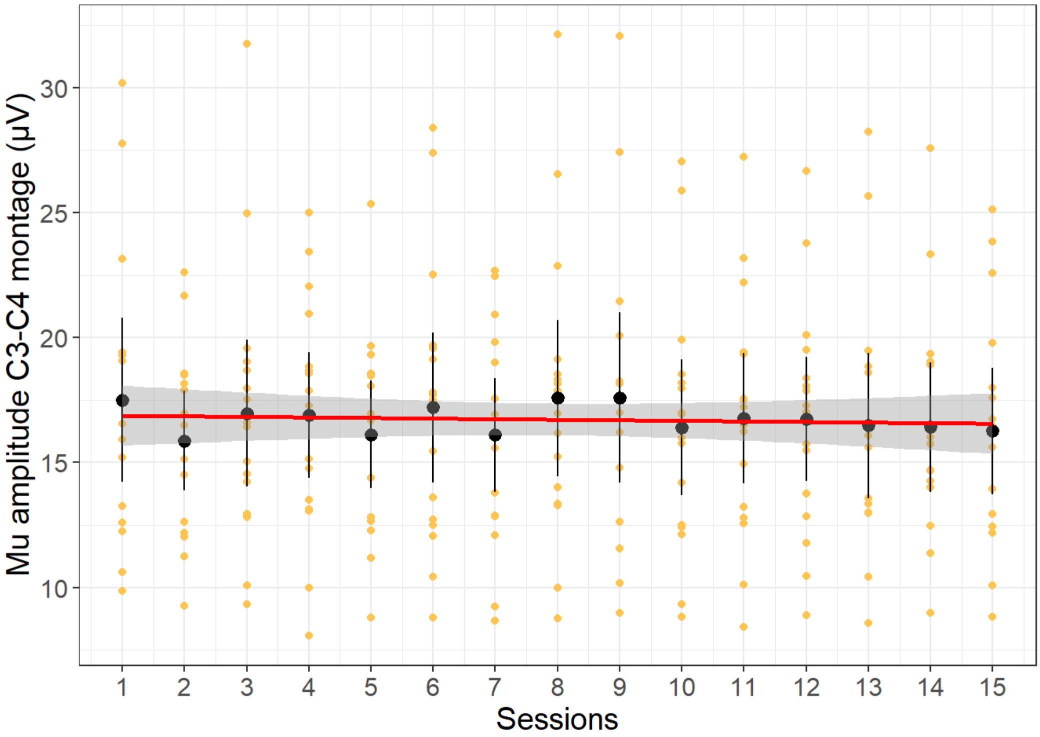


```
ggsave("C:/Users/dastg/Desktop/Main-Projects/Empra_2019_Dastgheib/Statistical_Analysis/Input/15VP-NFT/Output/Plots/Mean-err-Reg-NFT-Mu-C3C4-feedback.png", width = 8, height = 4, dpi = 300)

scatterNFT8 <- ggplot(NFT,aes(Session, BETA_C3.BETA_C4_feedback))
scatterNFT8 + geom_point(color="#fec44f") + labs(x = "Session", y = "Beta Voltage at C3.C4-Feedback")+ geom_smooth(method = "lm", colour = "Black")+scale_x_continuous(name="Sessions", limits=c(1, 15), breaks = c(1,2,3,4,5,6,7,8,9,10,11,12,13,14,15)) + theme_bw()
```

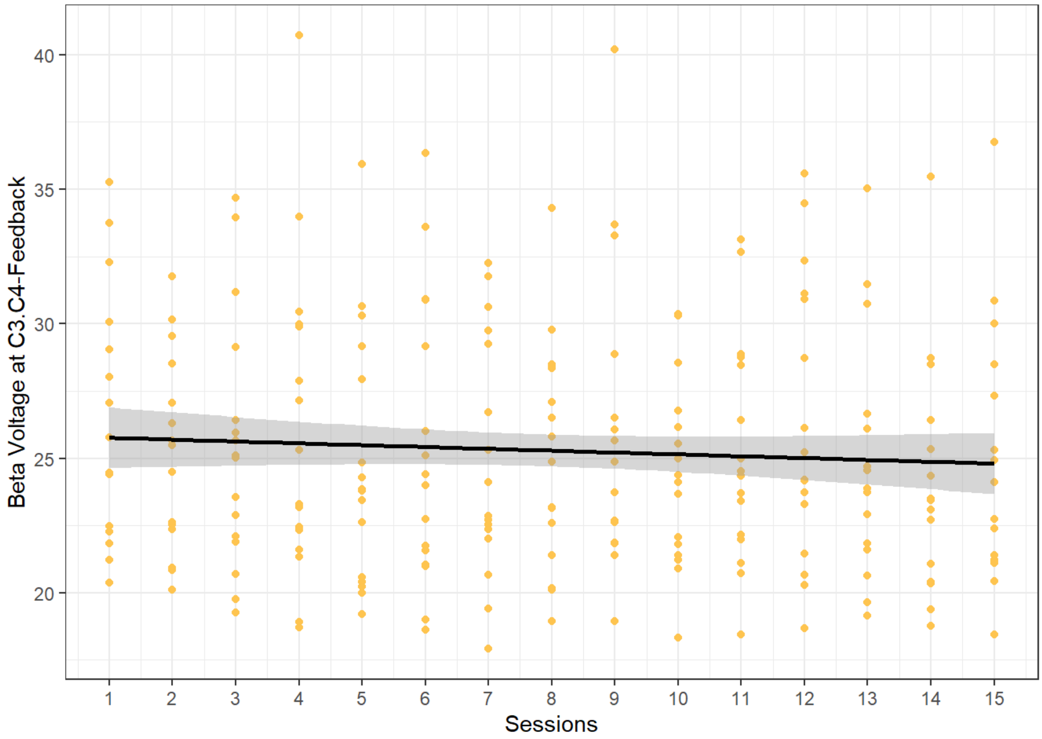


```
ggsave("C:/Users/dastg/Desktop/Main-Projects/Empra_2019_Dastgheib/Statistical_Analysis/Input/15VP-NFT/Output/Plots/Scatte
r-Reg-NFT-Mu-C3C4-feedback.png", width = 8, height = 4, dpi = 300)

scatterNFT9 <- ggplot(NFT,aes(Session, THETA_C3.THETA_C4_baseline))
scatterNFT9 + geom_point() + labs(x = "Session", y = "Theta Voltage at C3.C4-Baseline")+ geom_smooth(method = "lm", colou
r = "Red")
```

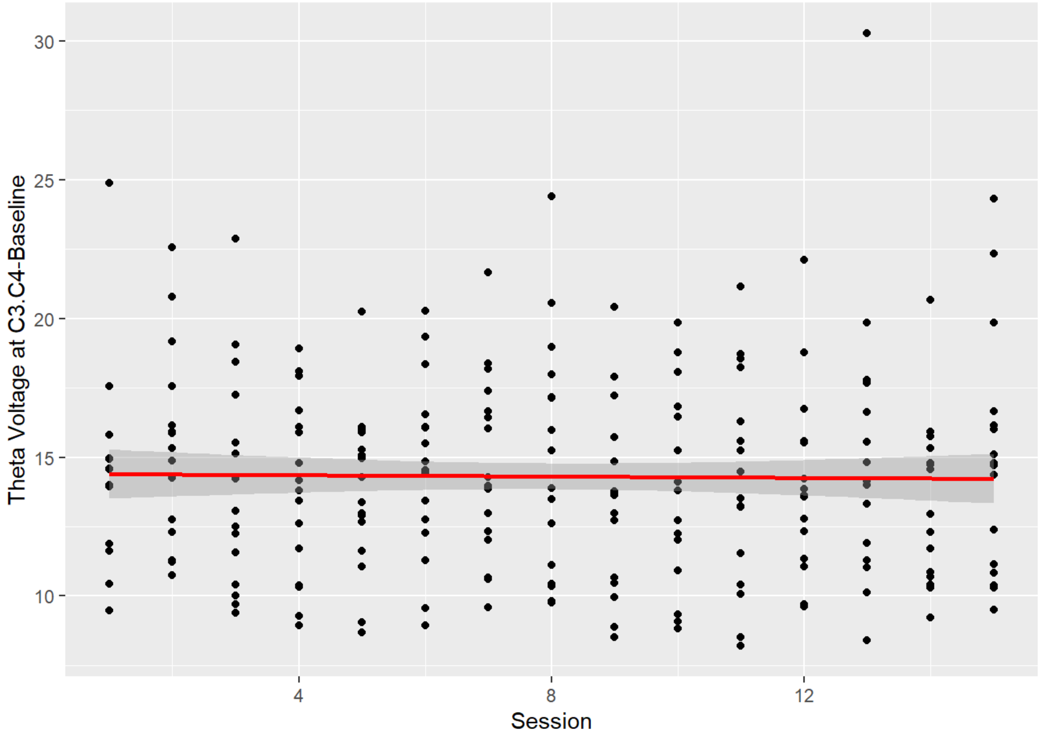


```
scatterNFT10 <- ggplot(NFT,aes(Session, THETA_C3.THETA_C4_feedback))
scatterNFT10 + geom_point() + labs(x = "Session", y = "Theta Voltage at C3.C4-Feedback")+ geom_smooth(method = "lm", colo
ur = "Red")
```

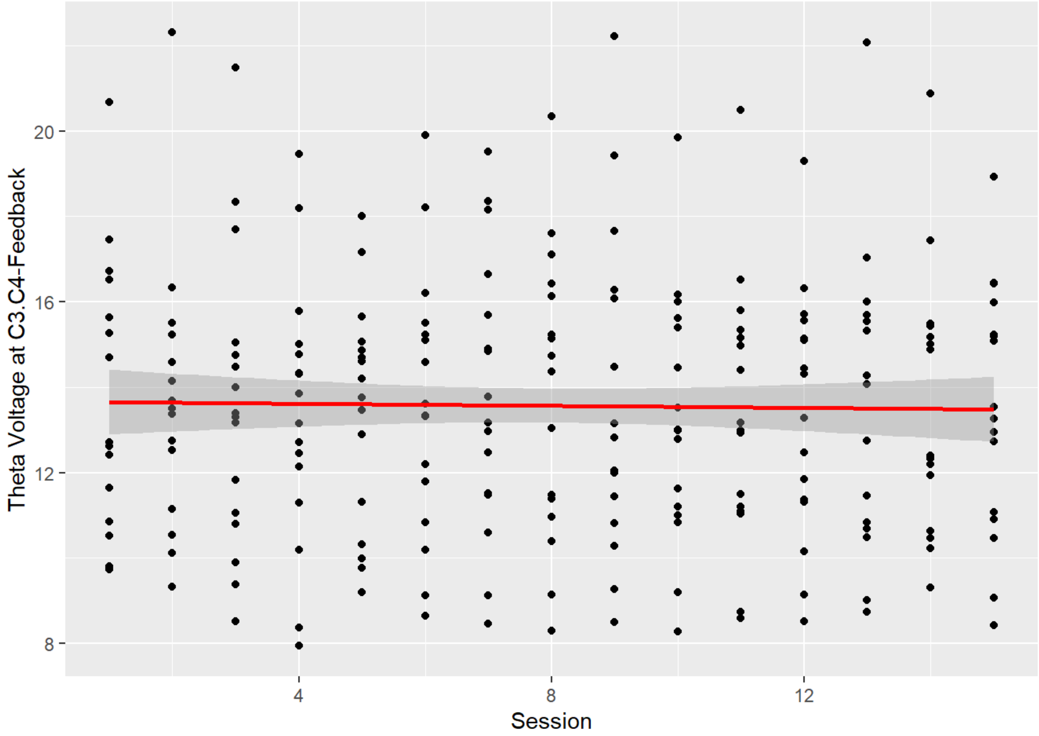


```
scatterNFT11 <- ggplot(NFT,aes(Session, EMGLOW_C3.EMGLOW_C4_baseline))
scatterNFT11 + geom_point() + labs(x = "Session", y = " Voltage at C3.C4-Baseline")+ geom_smooth(method = "lm", colour =
"Red")
```

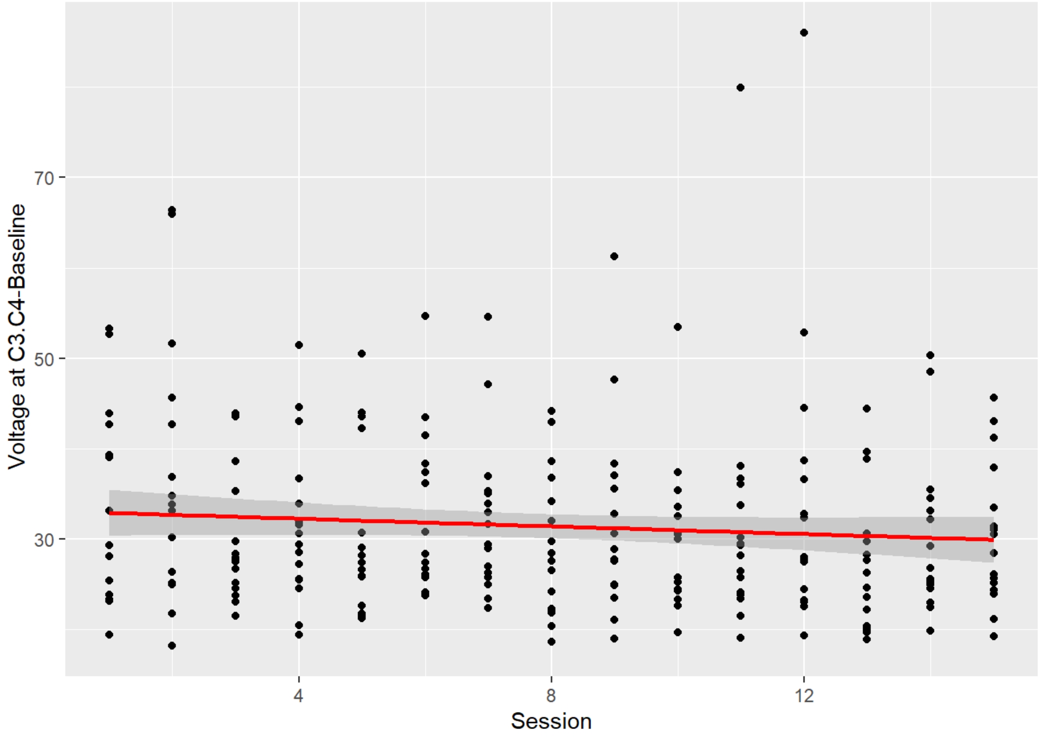


```
scatterNFT12 <- ggplot(NFT,aes(Session, EMGLOW_C3.EMGLOW_C4_feedback))
scatterNFT12 + geom_point() + labs(x = "Session", y = " Voltage at C3.C4-Feedbac")+ geom_smooth(method = "lm", colour =
"Red")
```

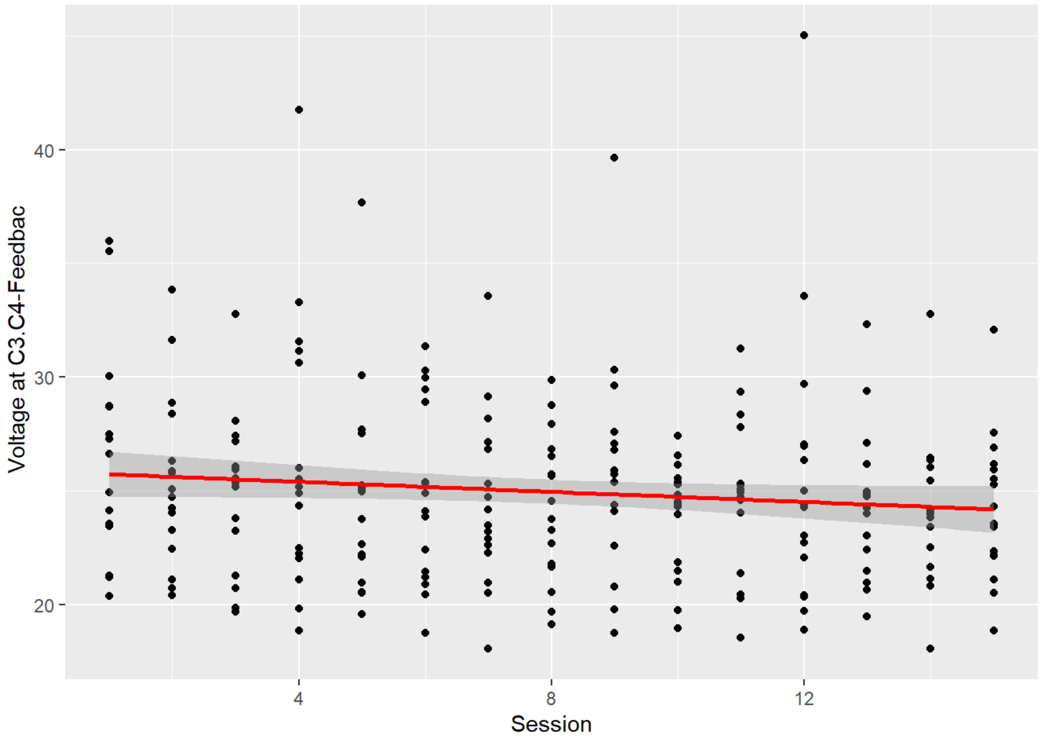

Plots based on the session groups

```
#---------------Plots based on the session groups-------

#Correctin names for Sgroup variable

NFT<-read.csv(file=("C:/Users/dastg/Desktop/Main-Projects/Empra_2019_Dastgheib/Statistical_Analysis/Input/15VP-NFT/NFT_da
ta.csv"),header = TRUE)

NFT$Session <- as.numeric(NFT$Session)
NFTA<-NFT[NFT$Session<6,];NFTA$Sgroup<-"Sessions 1-5"
NFTB<-NFT %>% filter(Session== "6"|Session=="7" | Session=="8"|Session=="9" | Session== "10");NFTB$Sgroup<-"Sessions 5-1
0"
NFTC<-NFT[NFT$Session>10,];NFTC$Sgroup<-"Sessions 10-15"
NFT<-rbind(NFTA,NFTB,NFTC)


NFT$Session <- factor(NFT$Session, levels=c("Sessions 1-5", "Sessions 5-10", "Sessions 10-15"))

write.csv(NFT,"C:\\Users\\dastg\\Desktop\\Main-Projects\\Empra_2019_Dastgheib\\Statistical_Analysis\\Raw-Data_17032021\\R
aw-NFT-16vp-Sessiongrouped.csv", row.names = FALSE)

#-------------Mu-C3-(baseline)-----------------

lineNFT<- ggplot( NFT, aes(Sgroup, MUE_C3_Baseline))
lineNFT + stat_summary(fun = mean, geom = "point") + stat_summary(fun = mean, geom = "line", aes(group = Sgroup))+ stat_s
ummary(fun.data = mean_cl_normal, geom = "errorbar")+ labs(x = "Session Group", y = "Voltage (µV)")+ theme_bw()
```

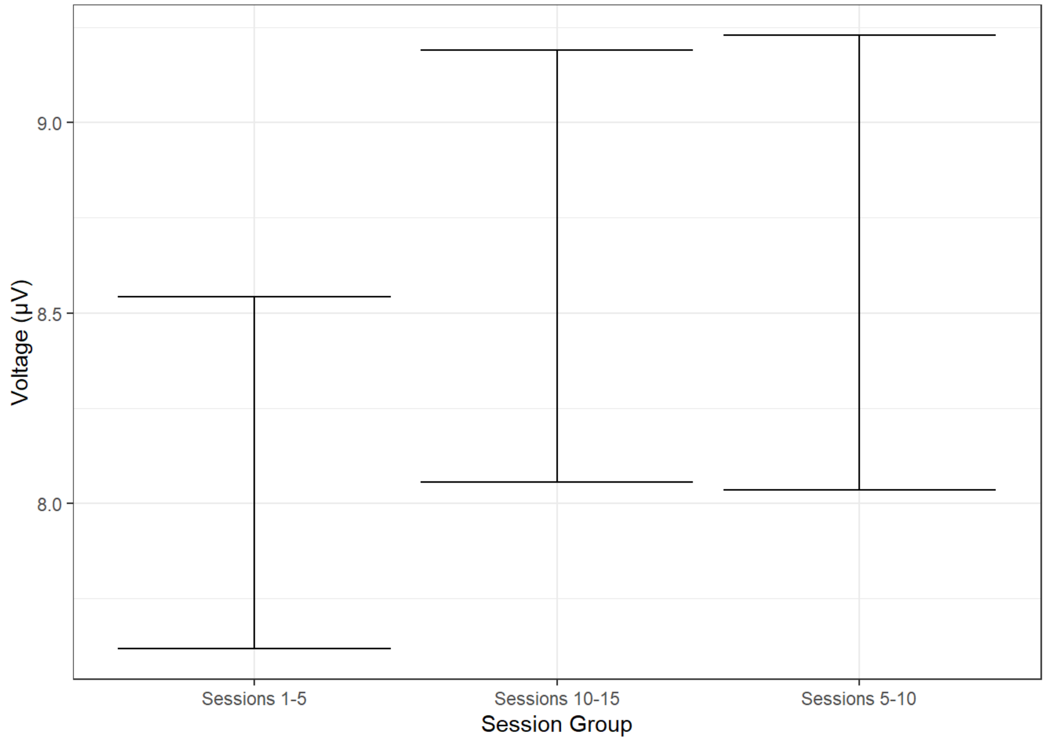


```
windowsFonts("Arial" = windowsFont("Arial"))
ggplot(NFT, aes(x=Sgroup, y=MUE_C3_Baseline))+ geom_point(aes(y= MUE_C3_Baseline),color="#fec44f",position =position_dodg
e(width = 0.5))+ stat_summary(fun = mean, geom = "point") + stat_summary(fun = mean, geom = "line", aes(group = Sgroup))+
stat_summary(fun.data = mean_cl_normal, geom = "errorbar")+ labs( x = "Session Group", y = "Voltage (µV)")+ theme_bw()+th
eme(text = element_text(size = 15, family = "Arial"))
```

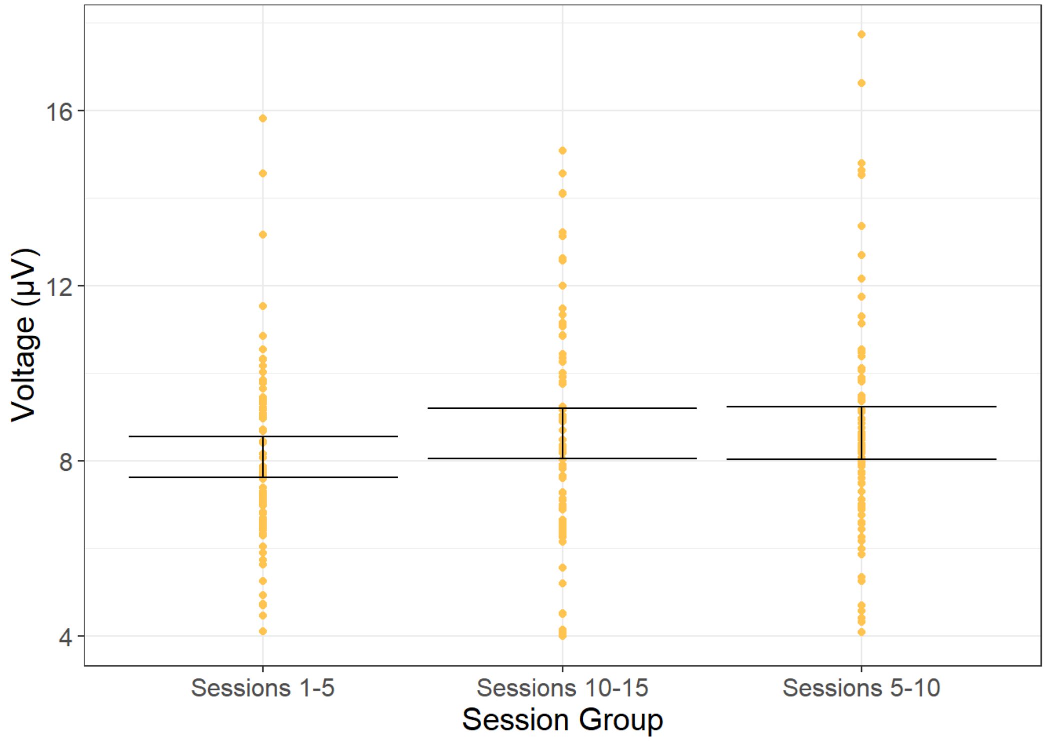


```
ggsave("C:/Users/dastg/Desktop/Main-Projects/Empra_2019_Dastgheib/Statistical_Analysis/Input/15VP-NFT/Output/Plots/NFT-Mu
-C3-B.png", width = 8, height = 4, dpi = 300)

#-----------------Mu-C3-(feedback)----------

lineNFT<- ggplot( NFT, aes(Sgroup, MUE_C3_Feedback))
lineNFT + stat_summary(fun = mean, geom = "point") + stat_summary(fun = mean, geom = "line", aes(group = Sgroup))+ stat_s
ummary(fun.data = mean_cl_normal, geom = "errorbar")+ labs(x = "Session Group", y = "Voltage (µV)")+ theme_bw()
```

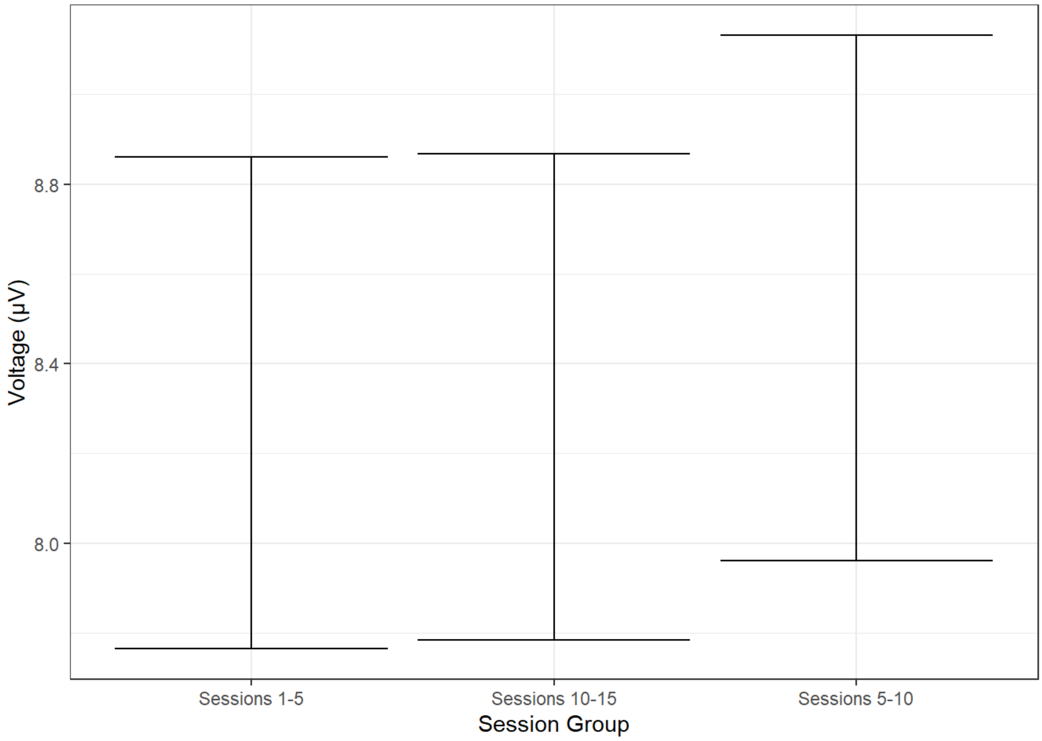


```
windowsFonts("Arial" = windowsFont("Arial"))
ggplot(NFT, aes(x=Sgroup, y=MUE_C3_Feedback))+ geom_point(aes(y= MUE_C3_Feedback),color="#fec44f",position =position_dodg
e(width = 0.5))+ stat_summary(fun = mean, geom = "point") + stat_summary(fun = mean, geom = "line", aes(group = Sgroup))+
stat_summary(fun.data = mean_cl_normal, geom = "errorbar")+ labs( x = "Session Group", y = "Voltage (µV)")+ theme_bw()+th
eme(text = element_text(size = 15, family = "Arial"))
```

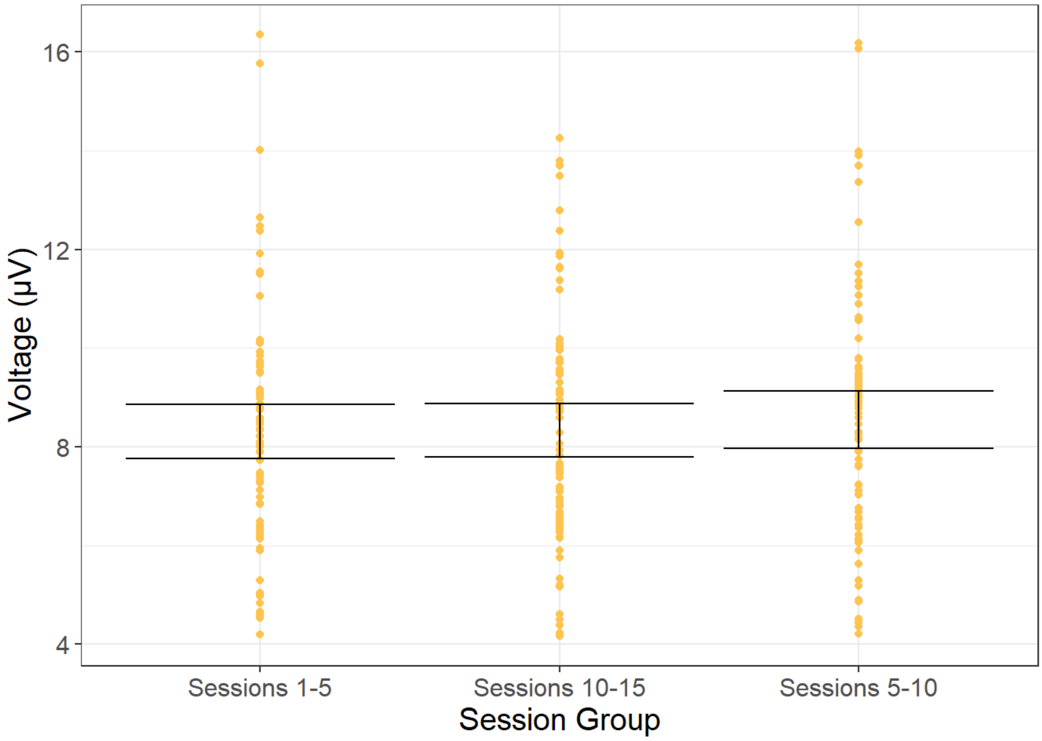


```
ggsave("C:/Users/dastg/Desktop/Main-Projects/Empra_2019_Dastgheib/Statistical_Analysis/Input/15VP-NFT/Output/Plots/NFT-Mu
-C3-F.png", width = 8, height = 4, dpi = 300)

#----------------MUE_C4_Baseline------------------

lineNFT<- ggplot( NFT, aes(Sgroup, MUE_C4_Baseline))
lineNFT + stat_summary(fun = mean, geom = "point") + stat_summary(fun = mean, geom = "line", aes(group = Sgroup))+ stat_s
ummary(fun.data = mean_cl_normal, geom = "errorbar")+ labs(x = "Session Group", y = "Voltage (µV)")+ theme_bw()
```

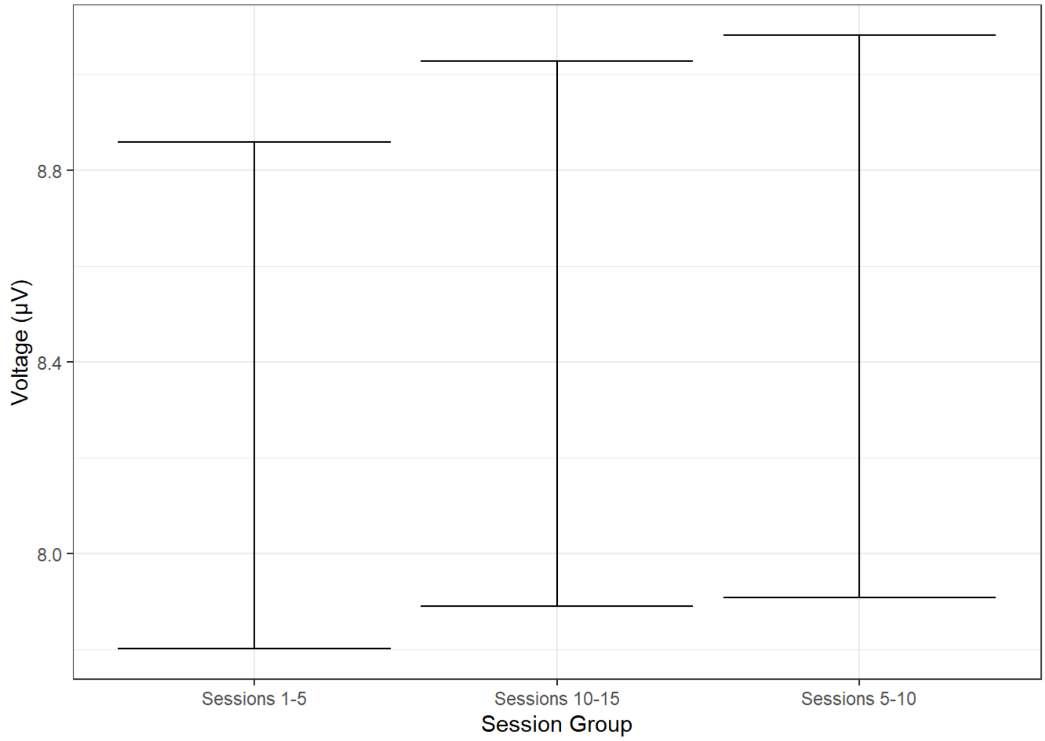


```
windowsFonts("Arial" = windowsFont("Arial"))
ggplot(NFT, aes(x=Sgroup, y=MUE_C4_Baseline))+ geom_point(aes(y= MUE_C4_Baseline),color="#fec44f",position =position_dodg
e(width = 0.5))+ stat_summary(fun = mean, geom = "point") + stat_summary(fun = mean, geom = "line", aes(group = Sgroup))+
stat_summary(fun.data = mean_cl_normal, geom = "errorbar")+ labs( x = "Session Group", y = "Voltage (µV)")+ theme_bw()+th
eme(text = element_text(size = 15, family = "Arial"))
```

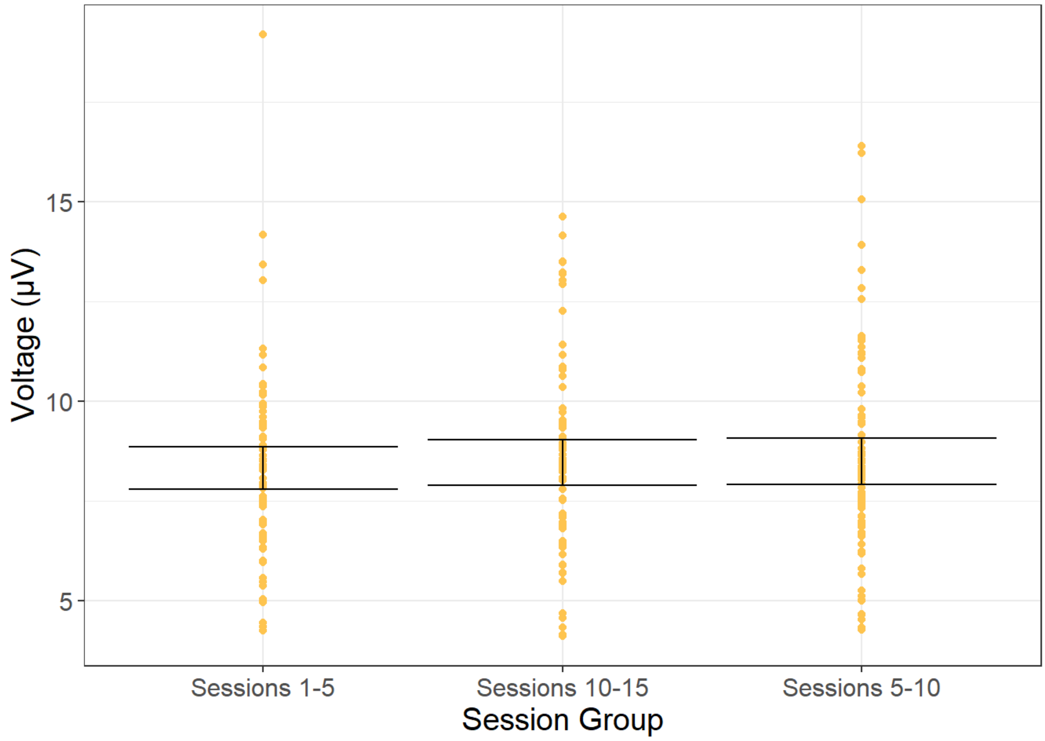


```
ggsave("C:/Users/dastg/Desktop/Main-Projects/Empra_2019_Dastgheib/Statistical_Analysis/Input/15VP-NFT/Output/Plots/NFT-Mu
-C4-B.png", width = 8, height = 4, dpi = 300)

#----------------MUE_C4_Feedback------------------

lineNFT<- ggplot( NFT, aes(Sgroup, MUE_C4_Feedback))
lineNFT + stat_summary(fun = mean, geom = "point") + stat_summary(fun = mean, geom = "line", aes(group = Sgroup))+ stat_s
ummary(fun.data = mean_cl_normal, geom = "errorbar")+ labs(x = "Session Group", y = "Voltage (µV)")+ theme_bw()
```

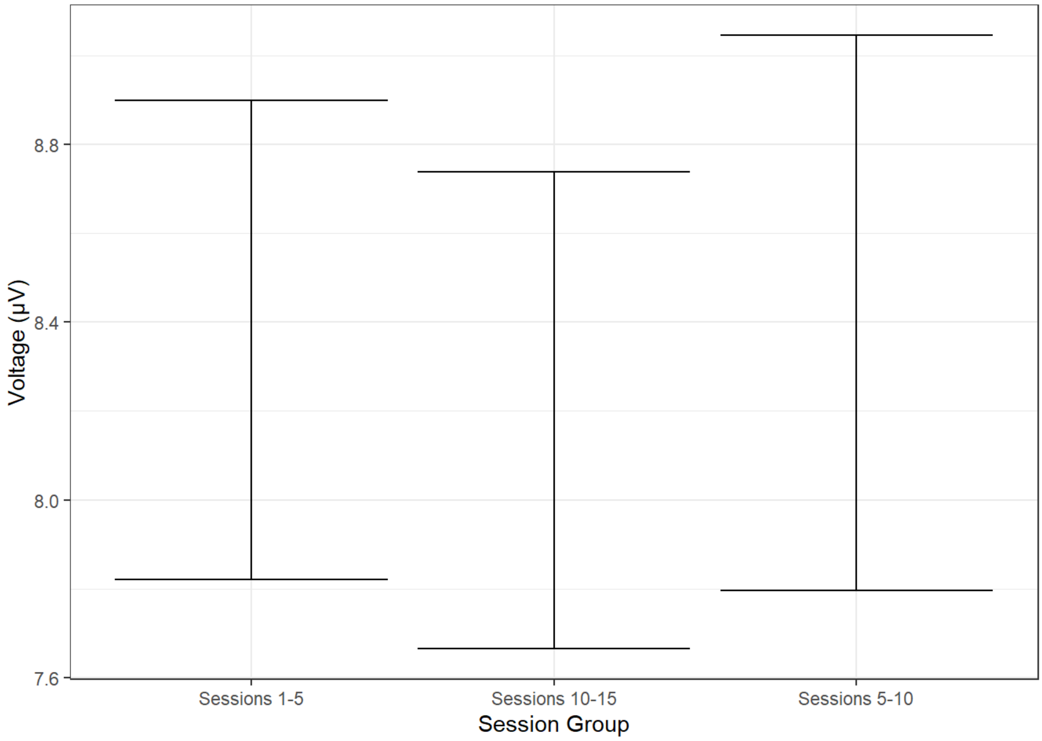


```
windowsFonts("Arial" = windowsFont("Arial"))
ggplot(NFT, aes(x=Sgroup, y=MUE_C4_Feedback))+ geom_point(aes(y= MUE_C4_Feedback),color="#fec44f",position =position_dodg
e(width = 0.5))+ stat_summary(fun = mean, geom = "point") + stat_summary(fun = mean, geom = "line", aes(group = Sgroup))+
stat_summary(fun.data = mean_cl_normal, geom = "errorbar")+ labs( x = "Session Group", y = "Voltage (µV)")+ theme_bw()+th
eme(text = element_text(size = 15, family = "Arial"))
```

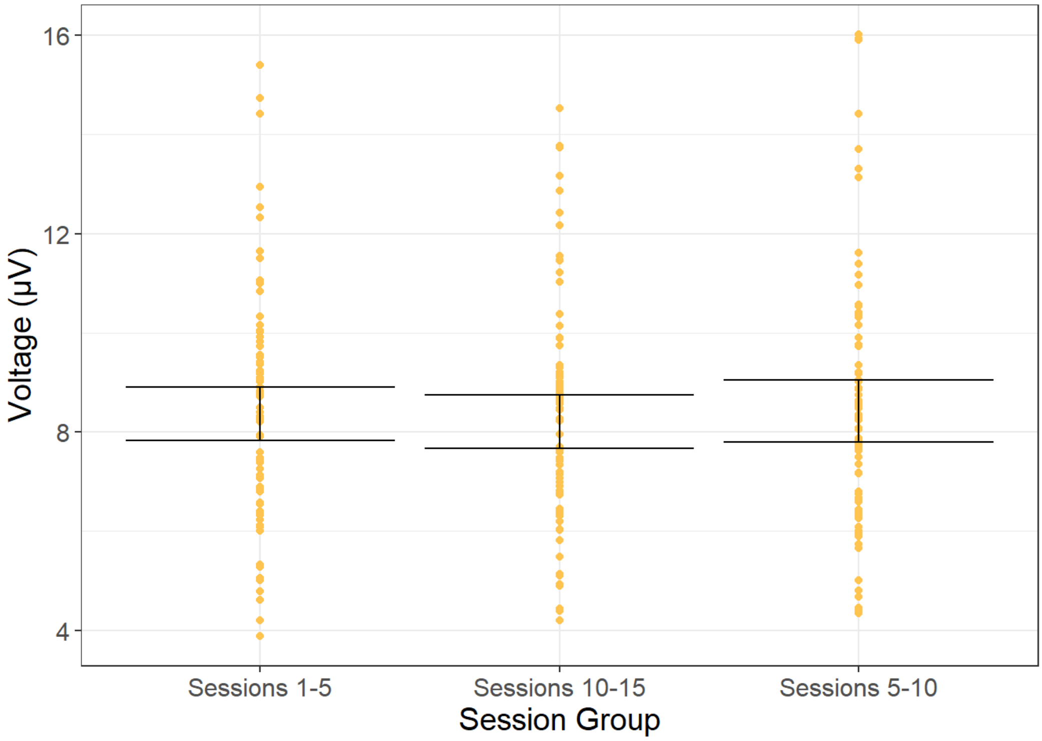


```
ggsave("C:/Users/dastg/Desktop/Main-Projects/Empra_2019_Dastgheib/Statistical_Analysis/Input/15VP-NFT/Output/Plots/NFT-Mu
-C4-F.png", width = 8, height = 4, dpi = 300)

#----------------MUE_C3-C4_Baseline------------------

lineNFT<- ggplot( NFT, aes(Sgroup, MUE_C3.C4_Baseline))
lineNFT + stat_summary(fun = mean, geom = "point") + stat_summary(fun = mean, geom = "line", aes(group = Sgroup))+ stat_s
ummary(fun.data = mean_cl_normal, geom = "errorbar")+ labs(x = "Session Group", y = "Voltage (µV)")+ theme_bw()
```

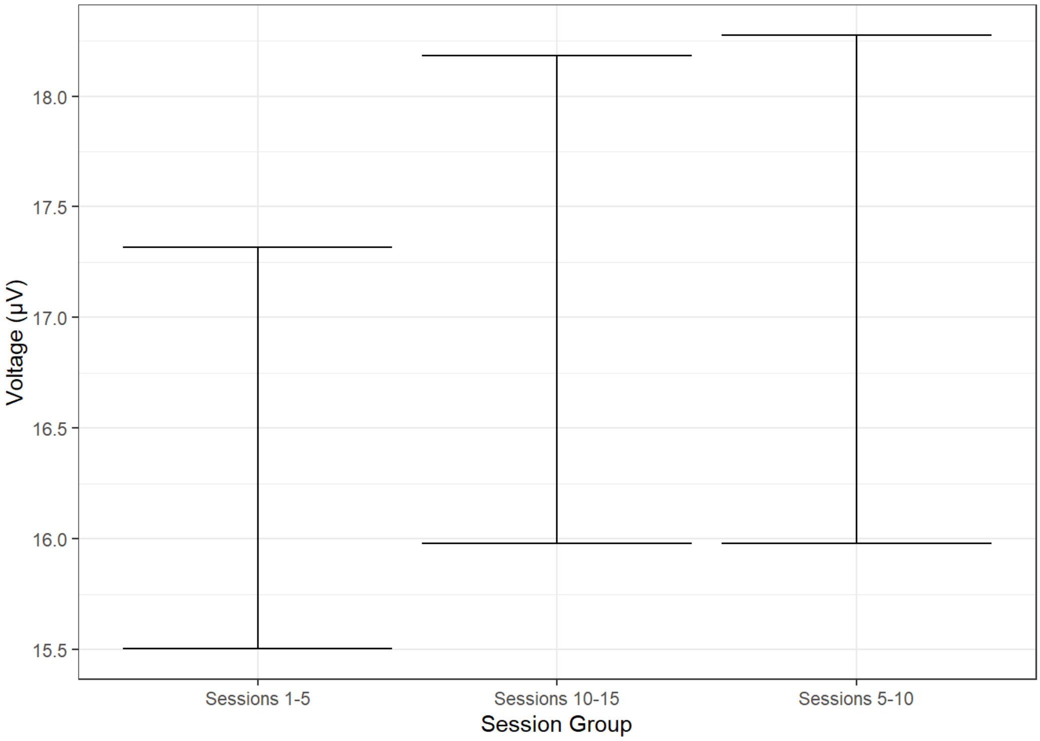


```
windowsFonts("Arial" = windowsFont("Arial"))
ggplot(NFT, aes(x=Sgroup, y=MUE_C3.C4_Baseline))+ geom_point(aes(y= MUE_C3.C4_Baseline),color="#fec44f",position =positio
n_dodge(width = 0.5))+ stat_summary(fun = mean, geom = "point") + stat_summary(fun = mean, geom = "line", aes(group = Sgr
oup))+stat_summary(fun.data = mean_cl_normal, geom = "errorbar")+ labs( x = "Session Group", y = "Voltage (µV)")+ theme_b
w()+theme(text = element_text(size = 15, family = "Arial"))
```

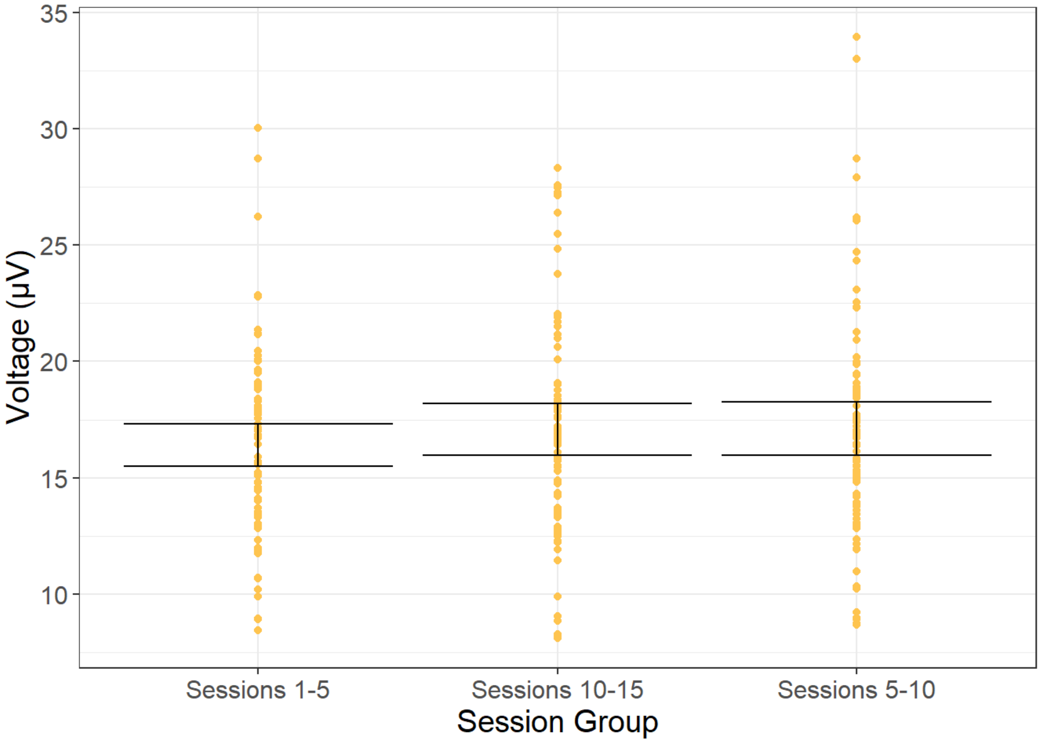


```
ggsave("C:/Users/dastg/Desktop/Main-Projects/Empra_2019_Dastgheib/Statistical_Analysis/Input/15VP-NFT/Output/Plots/NFT-Mu
-C3-C4-B.png", width = 8, height = 4, dpi = 300)

#----------------MUE_C3-C4_Feedback------------------

lineNFT<- ggplot( NFT, aes(Sgroup, MUE_C3.C4_Feedback))
lineNFT + stat_summary(fun = mean, geom = "point") + stat_summary(fun = mean, geom = "line", aes(group = Sgroup))+ stat_s
ummary(fun.data = mean_cl_normal, geom = "errorbar")+ labs(x = "Session Group", y = "Voltage (µV)")+ theme_bw()
```

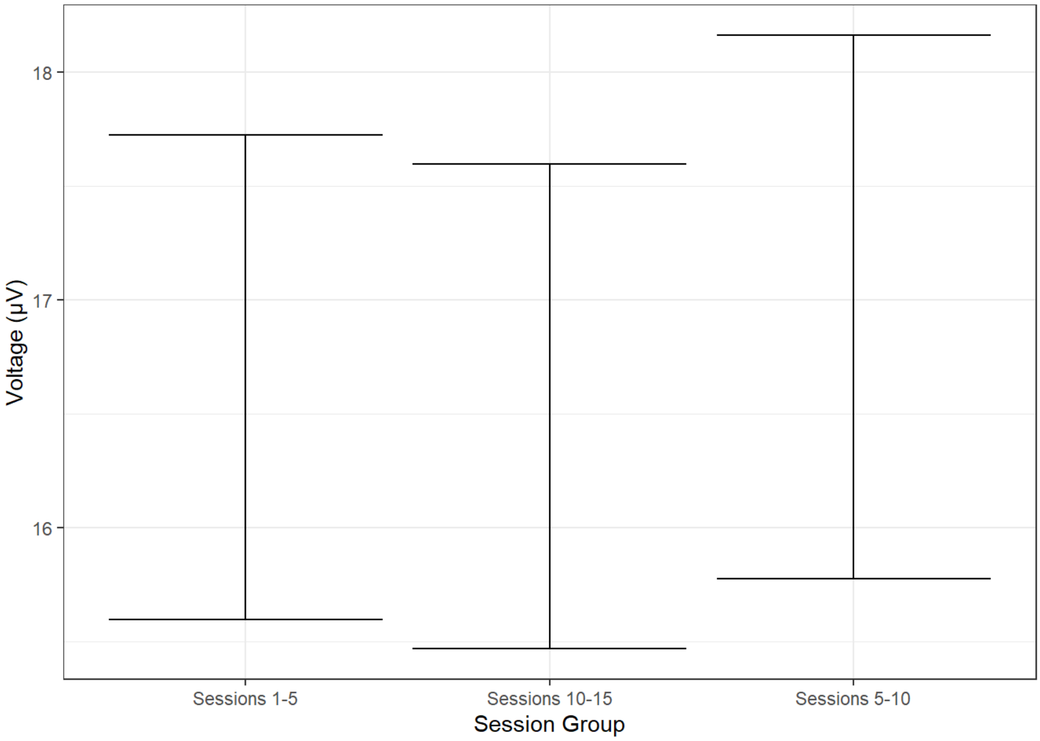


```
windowsFonts("Arial" = windowsFont("Arial"))
ggplot(NFT, aes(x=Sgroup, y=MUE_C3.C4_Feedback))+ geom_point(aes(y= MUE_C3.C4_Feedback),color="#fec44f",position =positio
n_dodge(width = 0.5))+ stat_summary(fun = mean, geom = "point") + stat_summary(fun = mean, geom = "line", aes(group = Sgr
oup))+stat_summary(fun.data = mean_cl_normal, geom = "errorbar")+ labs( x = "Session Group", y = "Voltage (µV)")+ theme_b
w()+theme(text = element_text(size = 15, family = "Arial"))
```

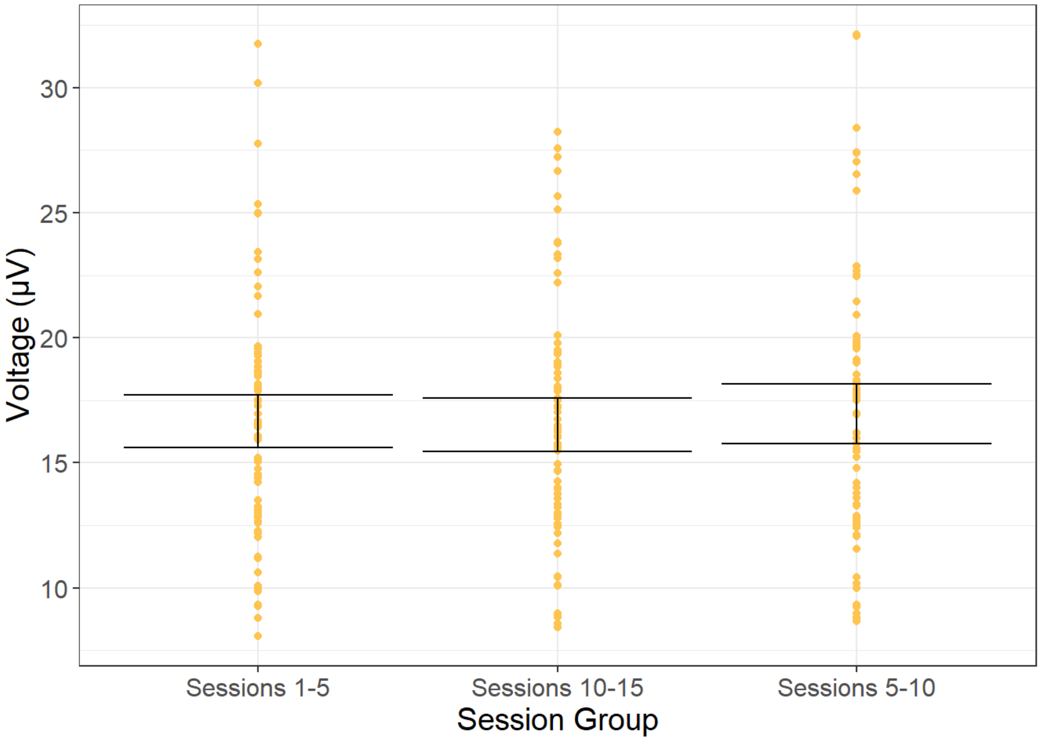


```
ggsave("C:/Users/dastg/Desktop/Main-Projects/Empra_2019_Dastgheib/Statistical_Analysis/Input/15VP-NFT/Output/Plots/NFT-Mu
-C3-C4-F.png", width = 8, height = 4, dpi = 300)

#----------------Beta_C3-C4_Baseline------------------

lineNFT<- ggplot( NFT, aes(Sgroup, BETA_C3.BETA_C4_baseline))
lineNFT + stat_summary(fun = mean, geom = "point") + stat_summary(fun = mean, geom = "line", aes(group = Sgroup))+ stat_s
ummary(fun.data = mean_cl_normal, geom = "errorbar")+ labs(x = "Session Group", y = "Voltage (µV)")+ theme_bw()
```

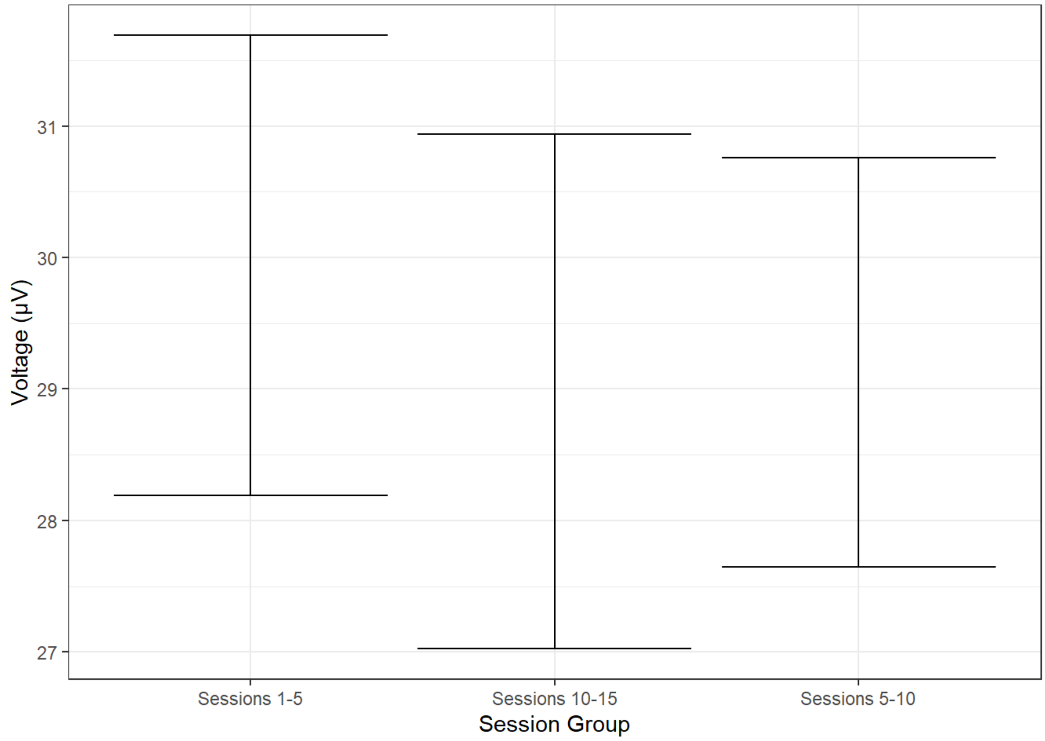


```
windowsFonts("Arial" = windowsFont("Arial"))
ggplot(NFT, aes(x=Sgroup, y=BETA_C3.BETA_C4_baseline))+ geom_point(aes(y= BETA_C3.BETA_C4_baseline),color="#fec44f",posit
ion =position_dodge(width = 0.5))+ stat_summary(fun = mean, geom = "point") + stat_summary(fun = mean, geom = "line", aes
(group = Sgroup))+stat_summary(fun.data = mean_cl_normal, geom = "errorbar")+ labs( x = "Session Group", y = "Voltage (µ
V)")+ theme_bw()+theme(text = element_text(size = 15, family = "Arial"))
```

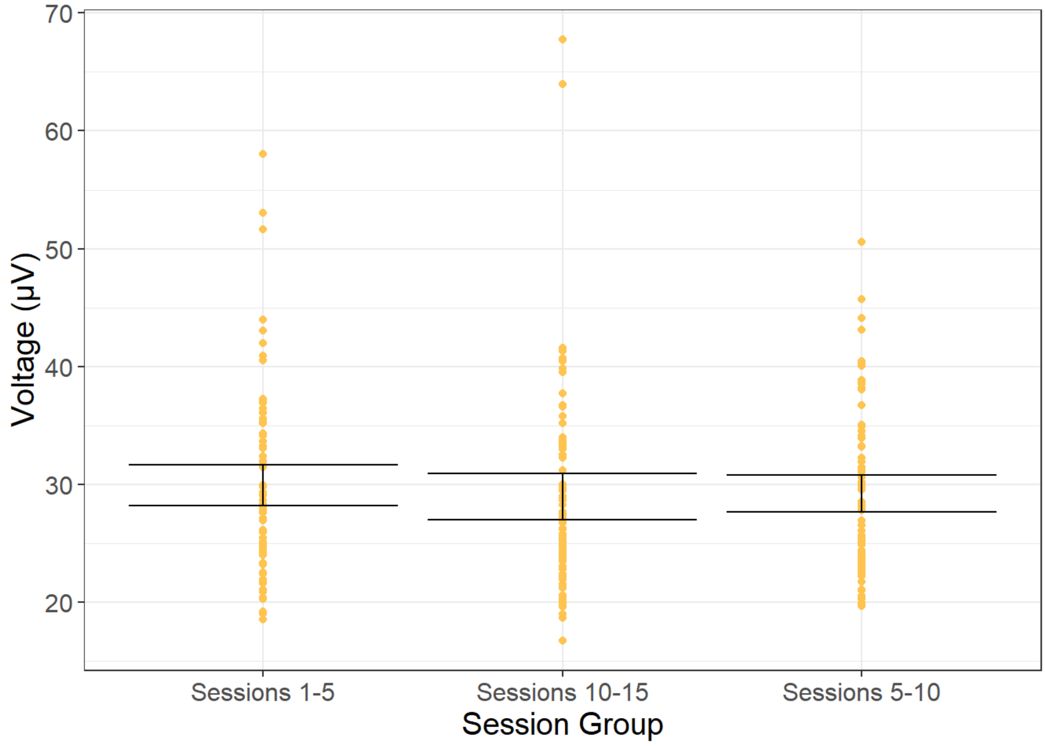


```
ggsave("C:/Users/dastg/Desktop/Main-Projects/Empra_2019_Dastgheib/Statistical_Analysis/Input/15VP-NFT/Output/Plots/NFT-Be
ta-C3-C4-B.png", width = 8, height = 4, dpi = 300)

#----------------Beta_C3-C4_Feedback------------------

lineNFT<- ggplot( NFT, aes(Sgroup, BETA_C3.BETA_C4_feedback))
lineNFT + stat_summary(fun = mean, geom = "point") + stat_summary(fun = mean, geom = "line", aes(group = Sgroup))+ stat_s
ummary(fun.data = mean_cl_normal, geom = "errorbar")+ labs(x = "Session Group", y = "Voltage (µV)")+ theme_bw()
```

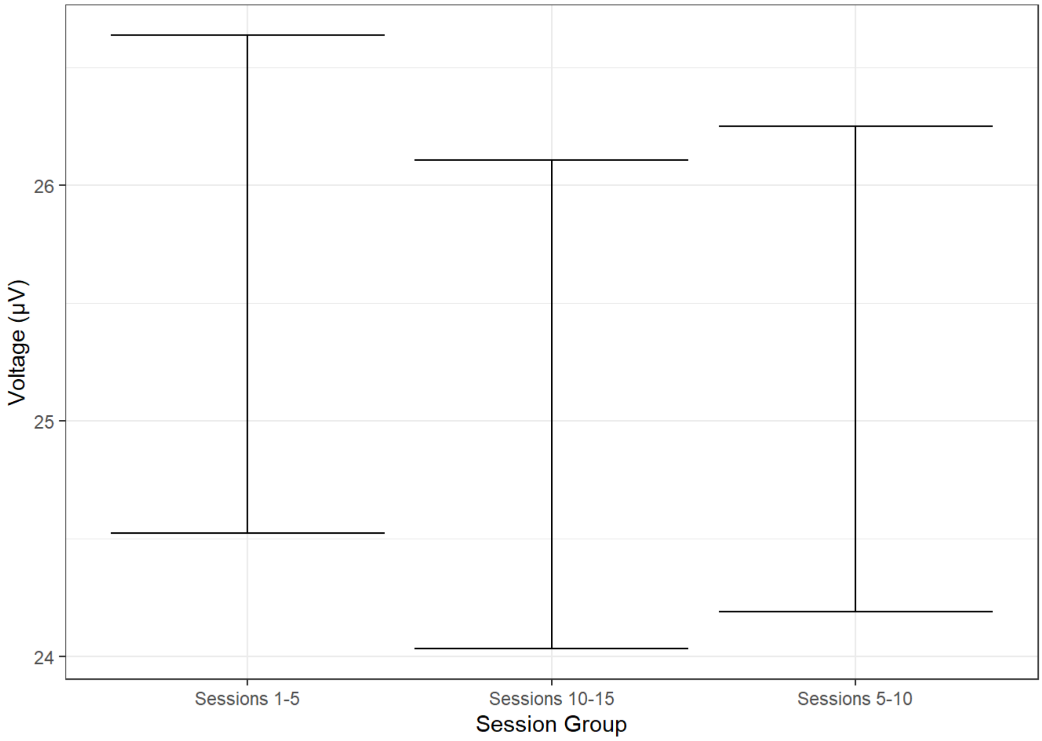


```
windowsFonts("Arial" = windowsFont("Arial"))
ggplot(NFT, aes(x=Sgroup, y=BETA_C3.BETA_C4_feedback))+ geom_point(aes(y= BETA_C3.BETA_C4_feedback),color="#fec44f",posit
ion =position_dodge(width = 0.5))+ stat_summary(fun = mean, geom = "point") + stat_summary(fun = mean, geom = "line", aes
(group = Sgroup))+stat_summary(fun.data = mean_cl_normal, geom = "errorbar")+ labs( x = "Session Group", y = "Voltage (µ
V)")+ theme_bw()+theme(text = element_text(size = 15, family = "Arial"))
```

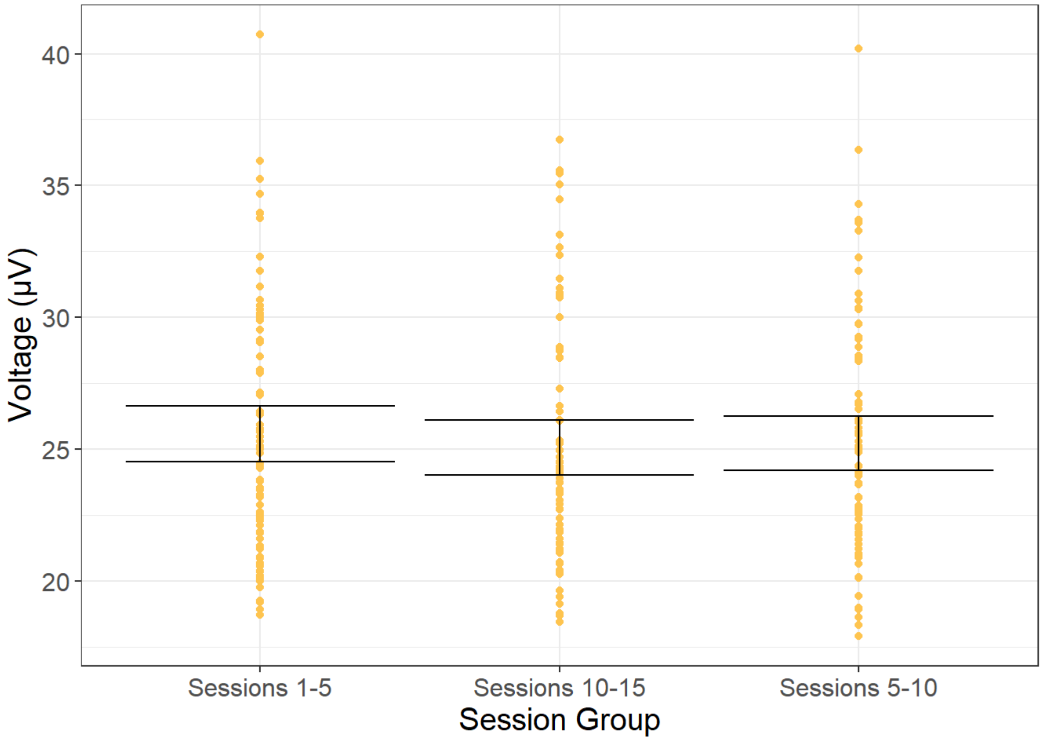


```
ggsave("C:/Users/dastg/Desktop/Main-Projects/Empra_2019_Dastgheib/Statistical_Analysis/Input/15VP-NFT/Output/Plots/NFT-Be
ta-C3-C4-F.png", width = 8, height = 4, dpi = 300)


#----------------Theta_C3-C4_Baseline------------------

lineNFT<- ggplot( NFT, aes(Sgroup, THETA_C3.THETA_C4_baseline))
lineNFT + stat_summary(fun = mean, geom = "point") + stat_summary(fun = mean, geom = "line", aes(group = Sgroup))+ stat_s
ummary(fun.data = mean_cl_normal, geom = "errorbar")+ labs(x = "Session Group", y = "Voltage (µV)")+ theme_bw()
```

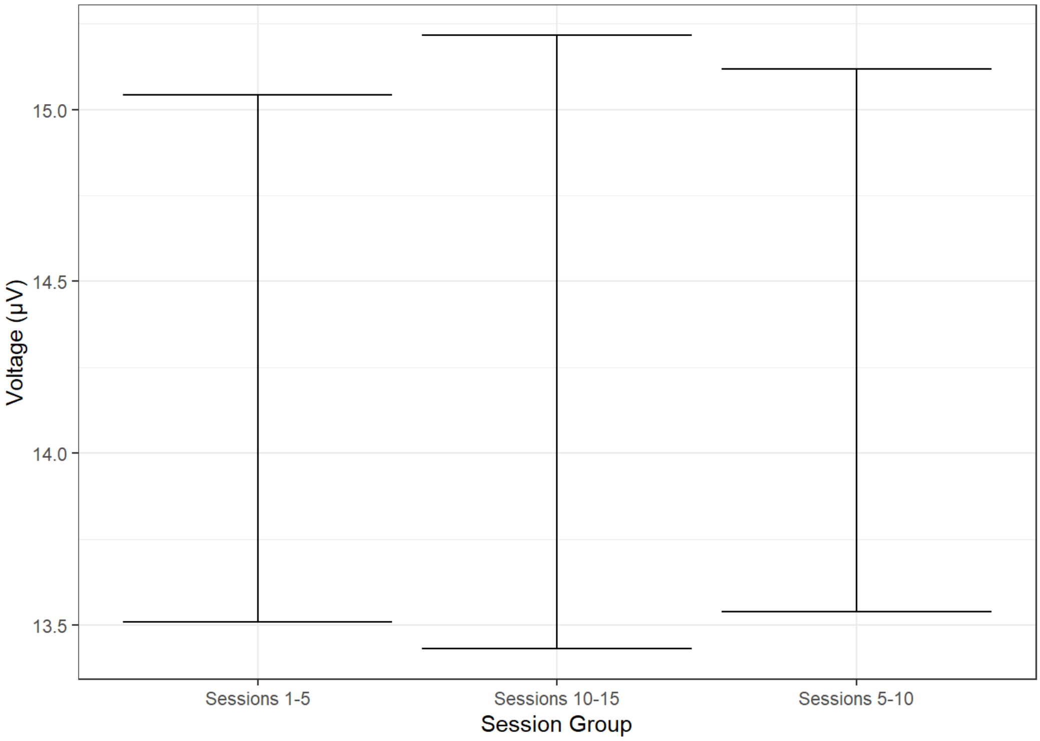


```
windowsFonts("Arial" = windowsFont("Arial"))
ggplot(NFT, aes(x=Sgroup, y=THETA_C3.THETA_C4_baseline))+ geom_point(aes(y= THETA_C3.THETA_C4_baseline),color="#fec44f",position =position_dodge(width = 0.5))+ stat_summary(fun = mean, geom = "point") + stat_summary(fun = mean, geom = "line", aes(group = Sgroup))+stat_summary(fun.data = mean_cl_normal, geom = "errorbar")+ labs( x = "Session Group", y = "Voltage (µV)")+ theme_bw()+theme(text = element_text(size = 15, family = "Arial"))
```

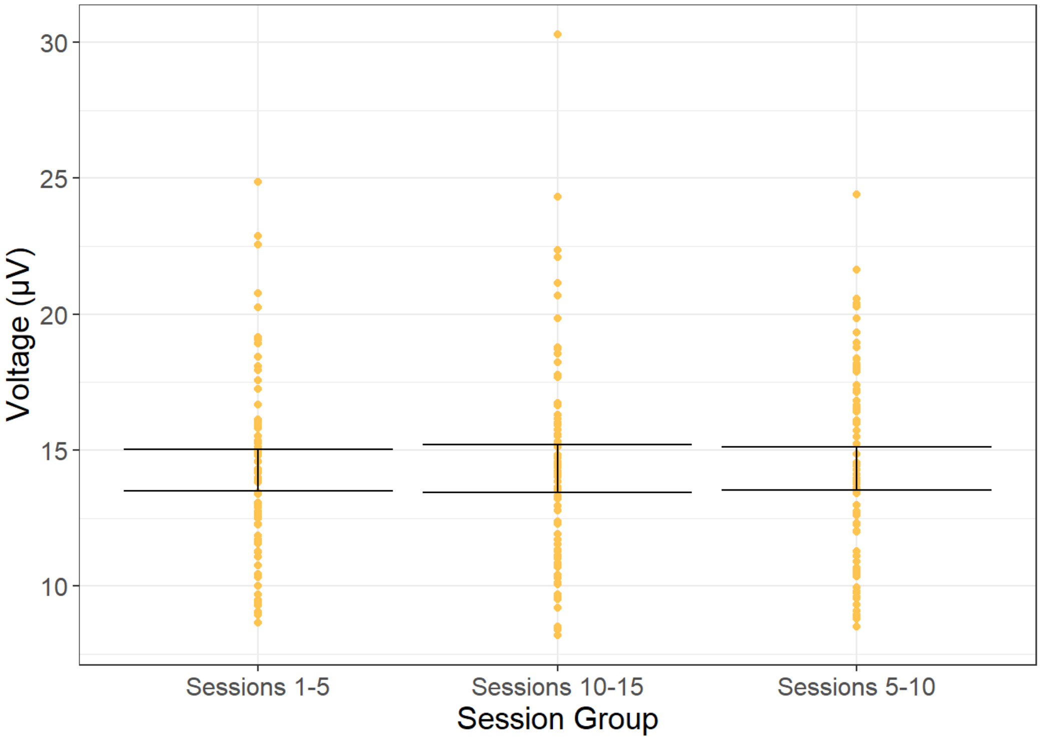


```
ggsave("C:/Users/dastg/Desktop/Main-Projects/Empra_2019_Dastgheib/Statistical_Analysis/Input/15VP-NFT/Output/Plots/NFT-Theta-C3-C4-B.png", width = 8, height = 4, dpi = 300)

#----------------Theta_C3-C4_Baseline------------------

lineNFT<- ggplot( NFT, aes(Sgroup, THETA_C3.THETA_C4_feedback))
lineNFT + stat_summary(fun = mean, geom = "point") + stat_summary(fun = mean, geom = "line", aes(group = Sgroup))+ stat_summary(fun.data = mean_cl_normal, geom = "errorbar")+ labs(x = "Session Group", y = "Voltage (µV)")+ theme_bw()
```

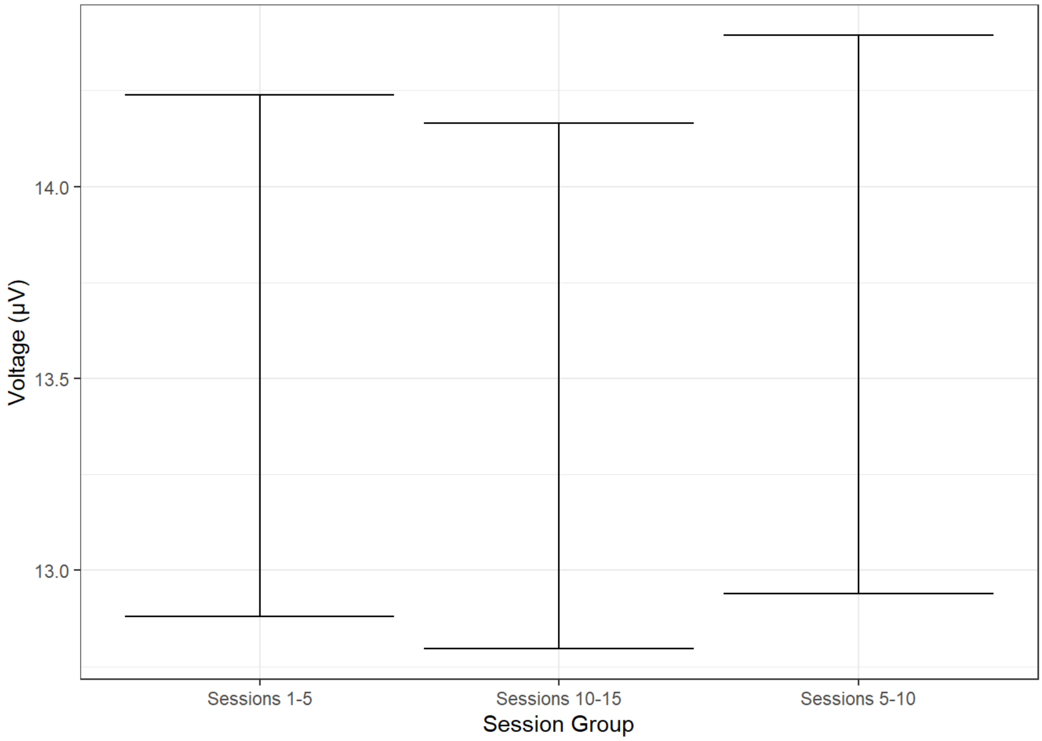


```
windowsFonts("Arial" = windowsFont("Arial"))
ggplot(NFT, aes(x=Sgroup, y=THETA_C3.THETA_C4_feedback))+ geom_point(aes(y= THETA_C3.THETA_C4_feedback),color="#fec44f",p
osition =position_dodge(width = 0.5))+ stat_summary(fun = mean, geom = "point") + stat_summary(fun = mean, geom = "line",
aes(group = Sgroup))+stat_summary(fun.data = mean_cl_normal, geom = "errorbar")+ labs( x = "Session Group", y = "Voltage
(µV)")+ theme_bw()+theme(text = element_text(size = 15, family = "Arial"))
```

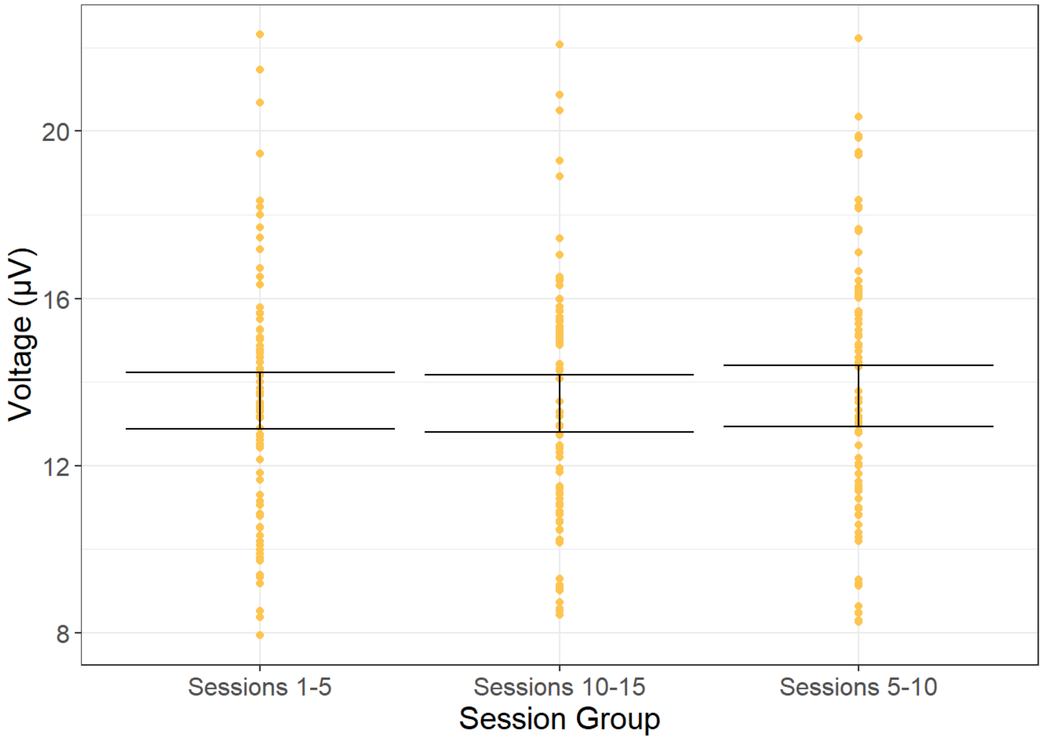


```
ggsave("C:/Users/dastg/Desktop/Main-Projects/Empra_2019_Dastgheib/Statistical_Analysis/Input/15VP-NFT/Output/Plots/NFT-Th
eta-C3-C4-F.png", width = 8, height = 4, dpi = 300)

#----------------Theta_C3-C4_Feedback------------------

lineNFT<- ggplot( NFT, aes(Sgroup, THETA_C3.THETA_C4_feedback))
lineNFT + stat_summary(fun = mean, geom = "point") + stat_summary(fun = mean, geom = "line", aes(group = Sgroup))+ stat_s
ummary(fun.data = mean_cl_normal, geom = "errorbar")+ labs(x = "Session Group", y = "Voltage (µV)")+ theme_bw()
```

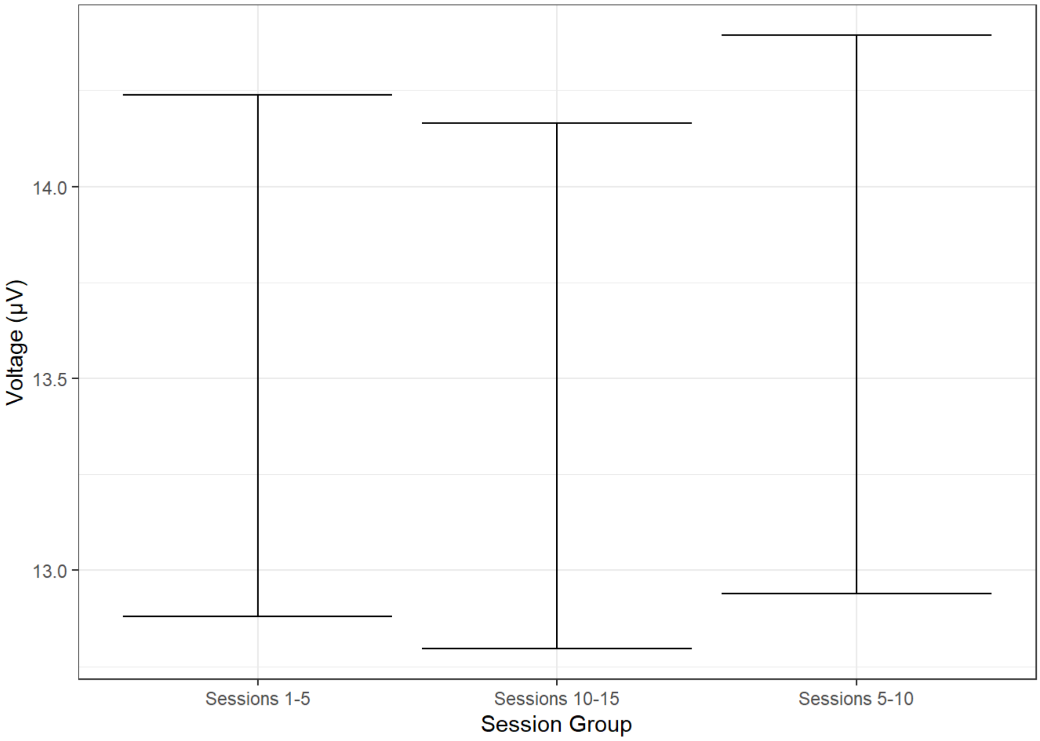


```
windowsFonts("Arial" = windowsFont("Arial"))
ggplot(NFT, aes(x=Sgroup, y=THETA_C3.THETA_C4_feedback))+ geom_point(aes(y= THETA_C3.THETA_C4_feedback),color="#fec44f",position =position_dodge(width = 0.5))+ stat_summary(fun = mean, geom = "point") + stat_summary(fun = mean, geom = "line", aes(group = Sgroup))+stat_summary(fun.data = mean_cl_normal, geom = "errorbar")+ labs( x = "Session Group", y = "Voltage (µV)")+ theme_bw()+theme(text = element_text(size = 15, family = "Arial"))
```

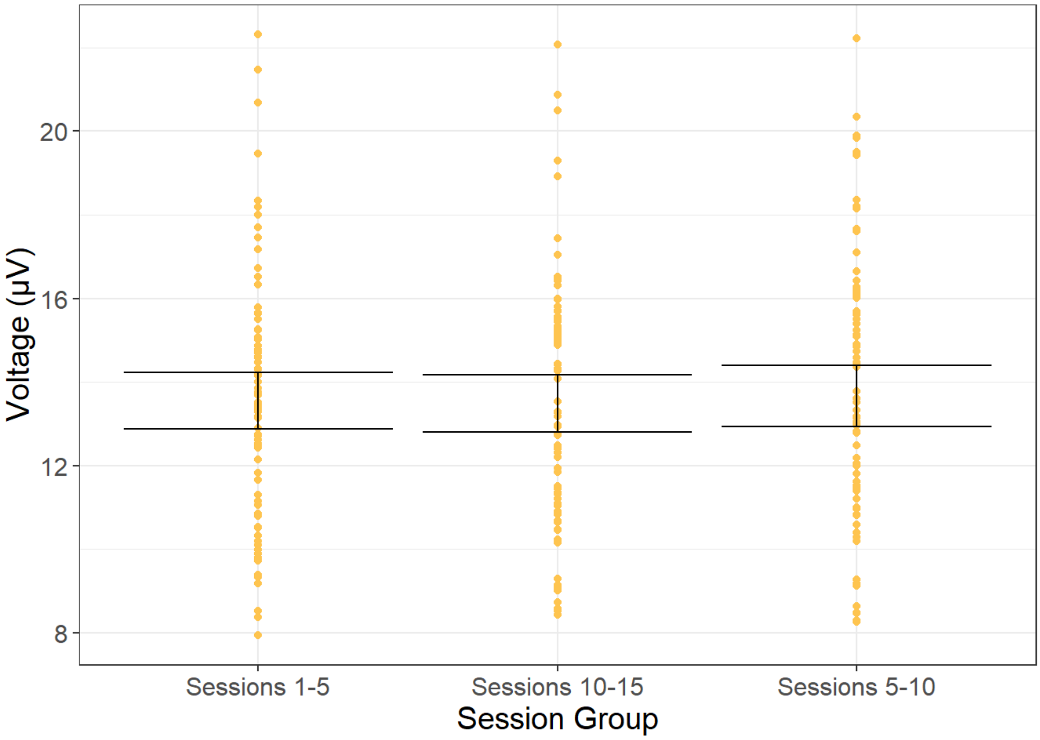


```
ggsave("C:/Users/dastg/Desktop/Main-Projects/Empra_2019_Dastgheib/Statistical_Analysis/Input/15VP-NFT/Output/Plots/NFT-Theta-C3-C4-F.png", width = 8, height = 4, dpi = 300)

#----------------EMG_C3-C4_Baseline------------------

lineNFT<- ggplot( NFT, aes(Sgroup, EMGLOW_C3.EMGLOW_C4_baseline))
lineNFT + stat_summary(fun = mean, geom = "point") + stat_summary(fun = mean, geom = "line", aes(group = Sgroup))+ stat_summary(fun.data = mean_cl_normal, geom = "errorbar")+ labs(x = "Session Group", y = "Voltage (µV)")+ theme_bw()
```

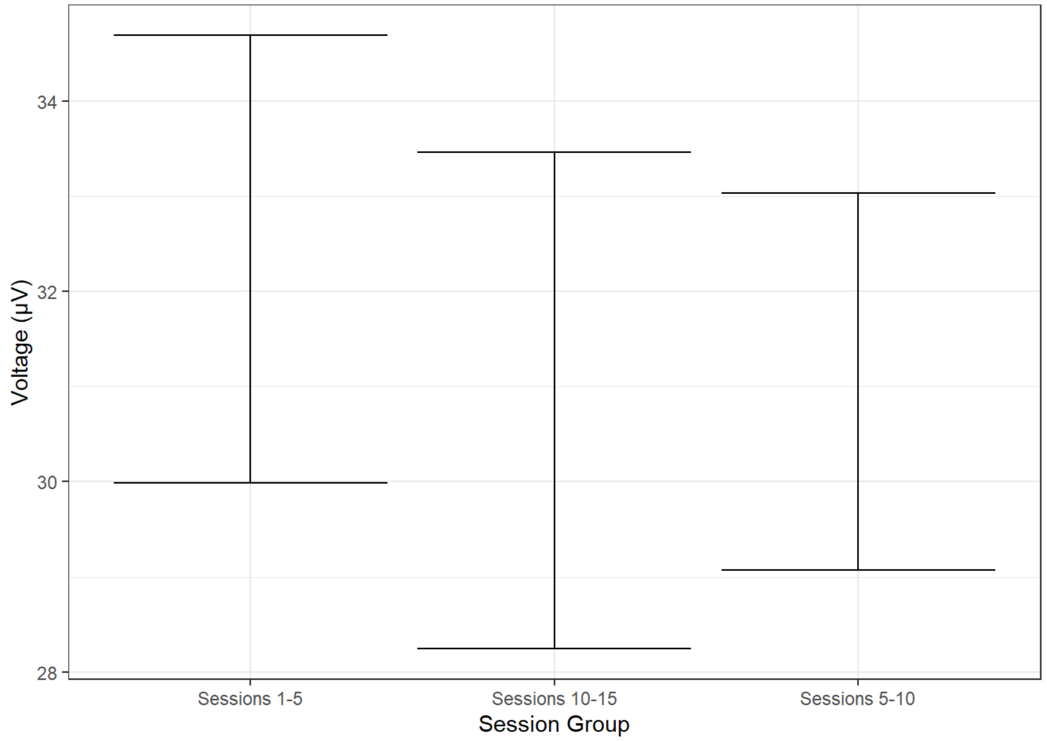


```
windowsFonts("Arial" = windowsFont("Arial"))
ggplot(NFT, aes(x=Sgroup, y=EMGLOW_C3.EMGLOW_C4_baseline))+ geom_point(aes(y= EMGLOW_C3.EMGLOW_C4_baseline),color="#fec44
f",position =position_dodge(width = 0.5))+ stat_summary(fun = mean, geom = "point") + stat_summary(fun = mean, geom = "li
ne", aes(group = Sgroup))+stat_summary(fun.data = mean_cl_normal, geom = "errorbar")+ labs( x = "Session Group", y = "Vol
tage (μV)")+ theme_bw()+theme(text = element_text(size = 15, family = "Arial"))
```

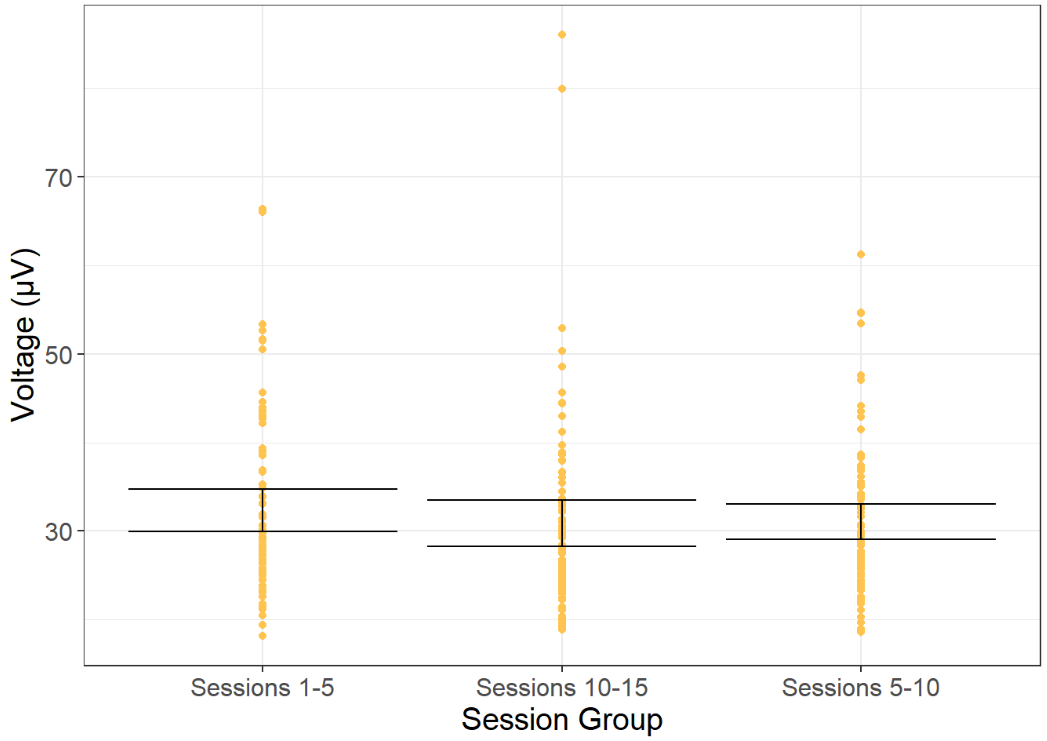


```
ggsave("C:/Users/dastg/Desktop/Main-Projects/Empra_2019_Dastgheib/Statistical_Analysis/Input/15VP-NFT/Output/Plots/NFT-EM
G-C3-C4-B.png", width = 8, height = 4, dpi = 300)

#----------------EMG_C3-C4_Feedback------------------

lineNFT<- ggplot( NFT, aes(Sgroup, EMGLOW_C3.EMGLOW_C4_feedback))
lineNFT + stat_summary(fun = mean, geom = "point") + stat_summary(fun = mean, geom = "line", aes(group = Sgroup))+ stat_s
ummary(fun.data = mean_cl_normal, geom = "errorbar")+ labs(x = "Session Group", y = "Voltage (μV)")+ theme_bw()
```

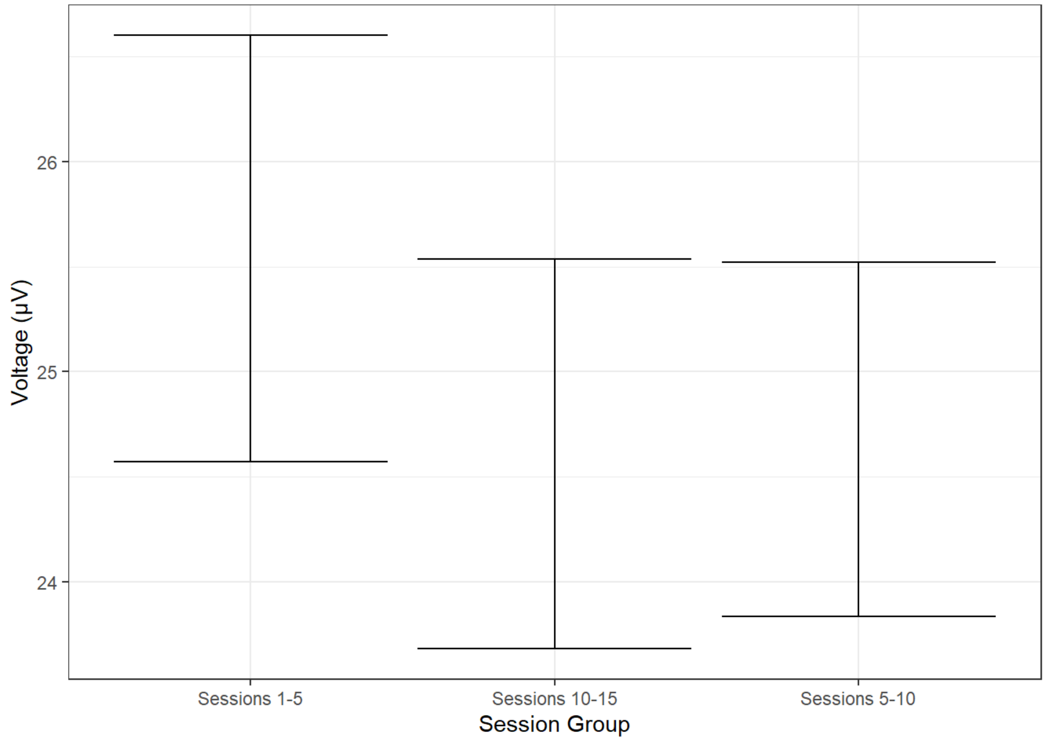


```
windowsFonts("Arial" = windowsFont("Arial"))
ggplot(NFT, aes(x=Sgroup, y=EMGLOW_C3.EMGLOW_C4_feedback ))+ geom_point(aes(y= EMGLOW_C3.EMGLOW_C4_feedback),color="#fec4
4f",position =position_dodge(width = 0.5))+ stat_summary(fun = mean, geom = "point") + stat_summary(fun = mean, geom = "l
ine", aes(group = Sgroup))+stat_summary(fun.data = mean_cl_normal, geom = "errorbar")+ labs( x = "Session Group", y = "Vo
ltage (µV)")+ theme_bw()+theme(text = element_text(size = 15, family = "Arial"))
```

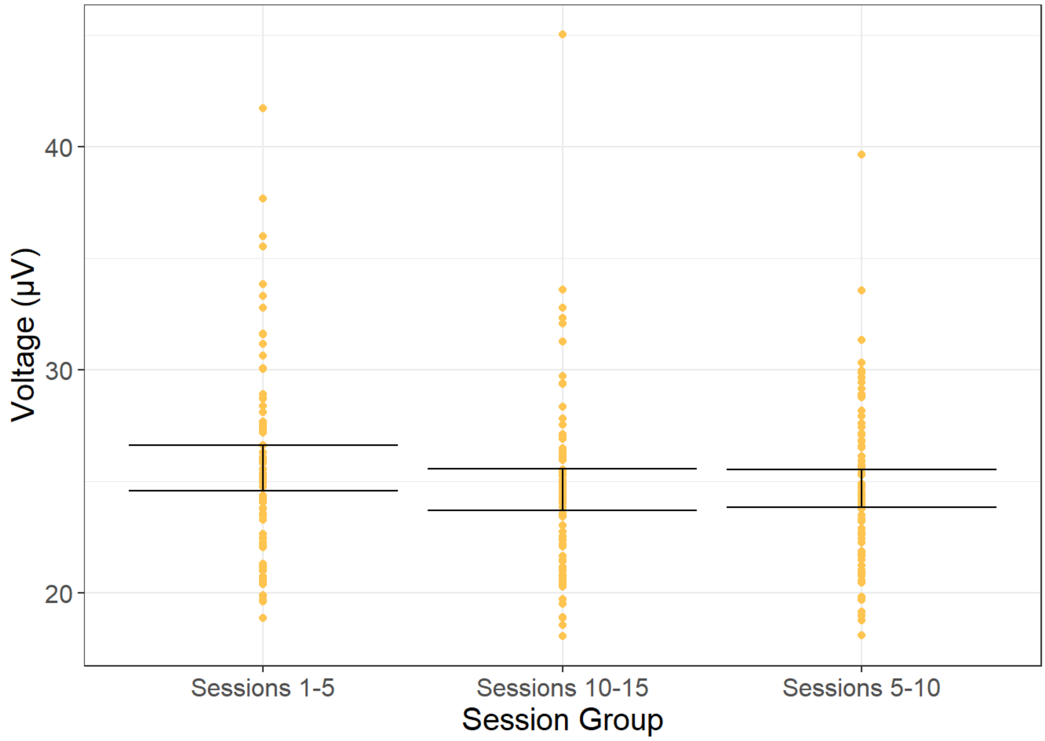


```
ggsave("C:/Users/dastg/Desktop/Main-Projects/Empra_2019_Dastgheib/Statistical_Analysis/Input/15VP-NFT/Output/Plots/NFT-EM
G-C3-C4-F.png", width = 8, height = 4, dpi = 300)
```

NFT sessions graph, based on the difference of feedback-baseline

```
#---------------------Graph the difference of (feedback)-(baseline)------
NFT<-read.csv(file=("C:/Users/dastg/Desktop/Main-Projects/Empra_2019_Dastgheib/Statistical_Analysis/Input/15VP-NFT/NFT_da
ta.csv"),header = TRUE)
NFT<- read_excel("C:/Users/dastg/Desktop/Main-Projects/Empra_2019_Dastgheib/Statistical_Analysis/Input/15VP-NFT/NFT_Excel
_data/NFT_data.xlsx")

#Create another Data base that has values for the difference of feedback-baseline.
NFT$Mu_C3_diff<-NFT$MUE_C3_Feedback-NFT$MUE_C3_Baseline;NFT$Mu_C4_diff<-NFT$MUE_C4_Feedback-NFT$MUE_C4_Baseline;NFT$Mu_C3
_C4_diff<-NFT$MUE_C3.C4_Feedback-NFT$MUE_C3.C4_Baseline;NFT$Theta_C3_C4_diff<-NFT$THETA_C3.THETA_C4_feedback-NFT$THETA_C
3.THETA_C4_baseline;NFT$Beta_C3_C4_diff<-NFT$BETA_C3.BETA_C4_feedback-NFT$BETA_C3.BETA_C4_baseline;NFT$EMG_C3_C4_diff<-NF
T$EMGLOW_C3.EMGLOW_C4_feedback-NFT$EMGLOW_C3.EMGLOW_C4_baseline

#-------------Mu_C3 diff--------------------
windowsFonts("Arial" = windowsFont("Arial"))
ggplot(NFT, aes(Session, Mu_C3_diff))+ geom_point(aes(y= Mu_C3_diff),color="#fec44f",position =position_dodge(width = 0.
5))+ stat_summary(fun = mean, geom = "line") + stat_summary(fun = mean, geom = "point", aes(group = Session))+ labs(x =
"Session", y = "Feedback-Baseline in Mu C3 montage (µV)")+ scale_x_continuous(name="Sessions", limits=c(1, 15), breaks =
c(1,2,3,4,5,6,7,8,9,10,11,12,13,14,15)) + theme_bw()+ theme(text = element_text(size = 15, family = "Arial"))+stat_summar
y(fun.data = mean_cl_normal, geom = "pointrange")+ geom_smooth(method = "lm", colour = "Red")
```

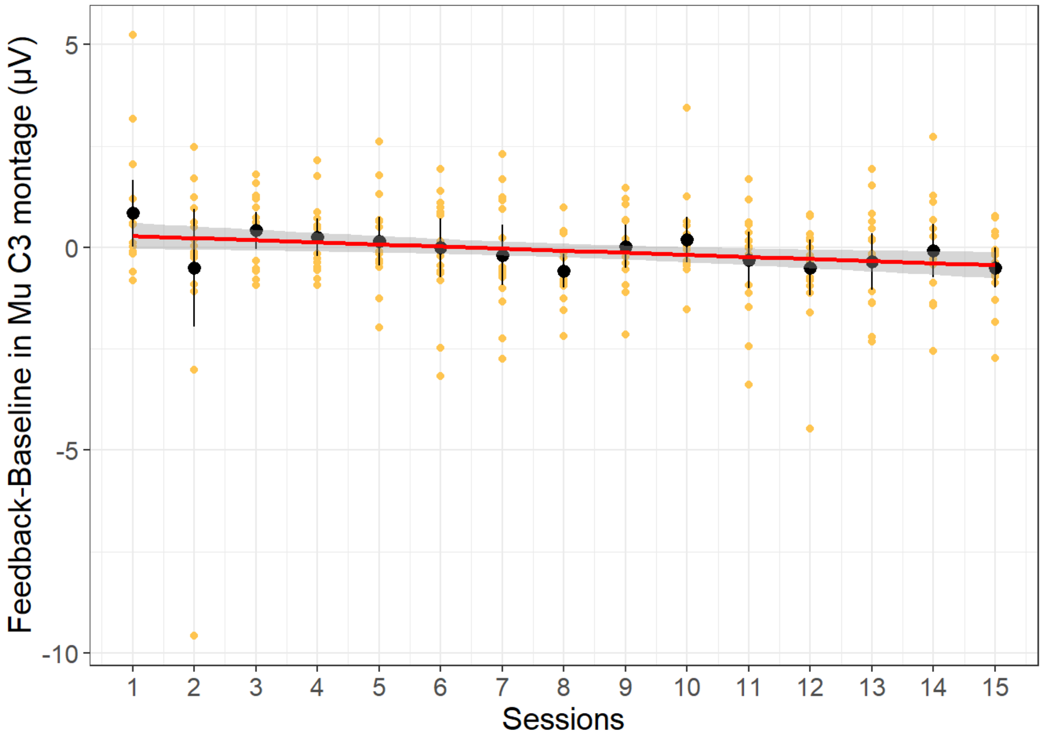


```
#-------------Mu_C4 diff--------------------
windowsFonts("Arial" = windowsFont("Arial"))
ggplot(NFT, aes(Session, Mu_C4_diff))+ geom_point(aes(y= Mu_C4_diff),color="#fec44f",position =position_dodge(width = 0.
5))+ stat_summary(fun = mean, geom = "line") + stat_summary(fun = mean, geom = "point", aes(group = Session))+ labs(x =
"Session", y = "Feedback-Baseline in Mu C4 montage (µV)")+ scale_x_continuous(name="Sessions", limits=c(1, 15), breaks =
c(1,2,3,4,5,6,7,8,9,10,11,12,13,14,15)) + theme_bw()+ theme(text = element_text(size = 15, family = "Arial"))+stat_summar
y(fun.data = mean_cl_normal, geom = "pointrange")+ geom_smooth(method = "lm", colour = "Red")
```

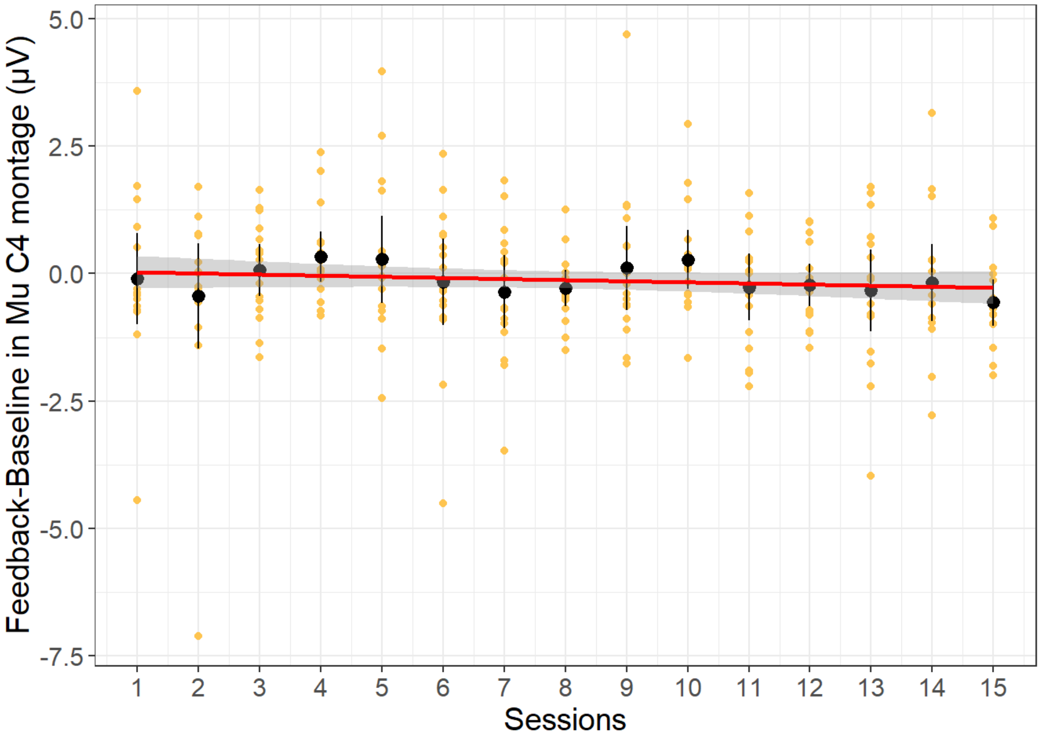


```
#-------------Mu_C3_C4 diff--------------------
windowsFonts("Arial" = windowsFont("Arial"))
ggplot(NFT, aes(Session, Mu_C3_C4_diff))+ geom_point(aes(y= Mu_C3_C4_diff),color="#fec44f",position =position_dodge(width
= 0.5))+ stat_summary(fun = mean, geom = "line") + stat_summary(fun = mean, geom = "point", aes(group = Session))+ labs(x
= "Session", y = "Feedback-Baseline in Mu C3-C4 montage (µV)")+ scale_x_continuous(name="Sessions", limits=c(1, 15), brea
ks = c(1,2,3,4,5,6,7,8,9,10,11,12,13,14,15)) + theme_bw()+ theme(text = element_text(size = 15, family = "Arial"))+stat_s
ummary(fun.data = mean_cl_normal, geom = "pointrange")+ geom_smooth(method = "lm", colour = "Red")
```

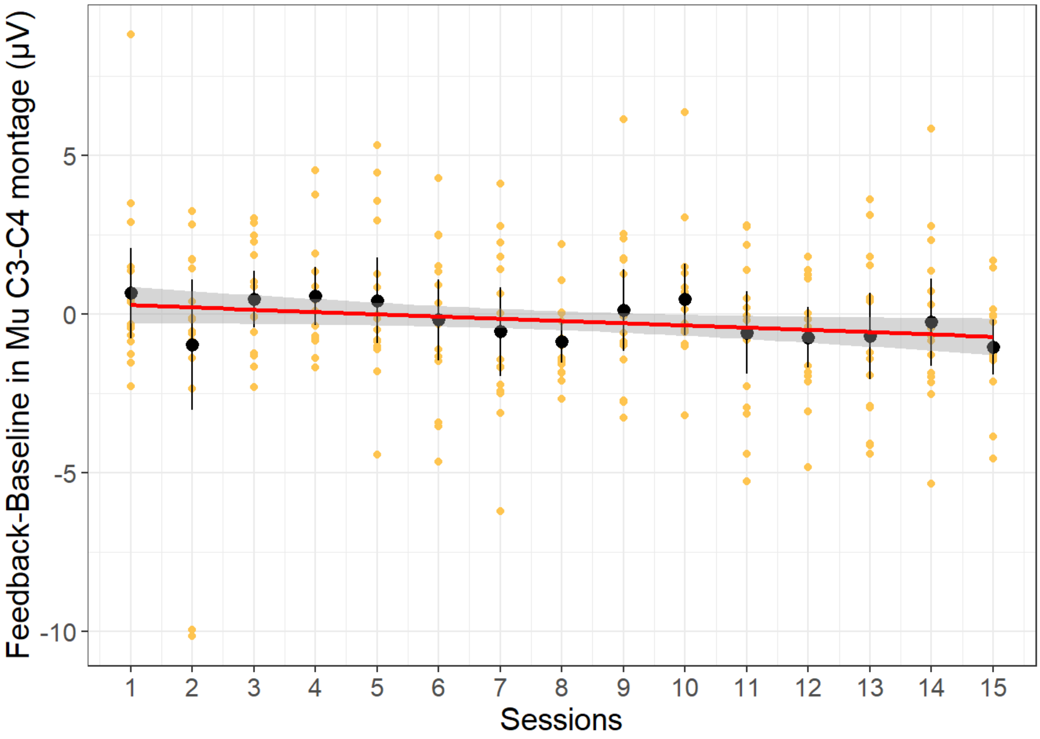

```
ggsave("C:/Users/dastg/Desktop/Main-Projects/Empra_2019_Dastgheib/Statistical_Analysis/Input/15VP-NFT/Output/Plots/NFT-Mu-C3-C4-diff.png", width = 8, height = 4, dpi = 300)
#-------------Theta_C3_C4 diff--------------------
windowsFonts("Arial" = windowsFont("Arial"))
ggplot(NFT, aes(Session, Theta_C3_C4_diff))+ geom_point(aes(y=Theta_C3_C4_diff),color="#fec44f",position =position_dodge(width = 0.5))+ stat_summary(fun = mean, geom = "line") + stat_summary(fun = mean, geom = "point", aes(group = Session))+ labs(x = "Session", y = "Feedback-Baseline in Theta C3-C4 montage (µV)")+ scale_x_continuous(name="Sessions", limits=c(1, 15), breaks = c(1,2,3,4,5,6,7,8,9,10,11,12,13,14,15)) + theme_bw()+ theme(text = element_text(size = 15, family = "Arial"))+stat_summary(fun.data = mean_cl_normal, geom = "pointrange")+ geom_smooth(method = "lm", colour = "Red")
```

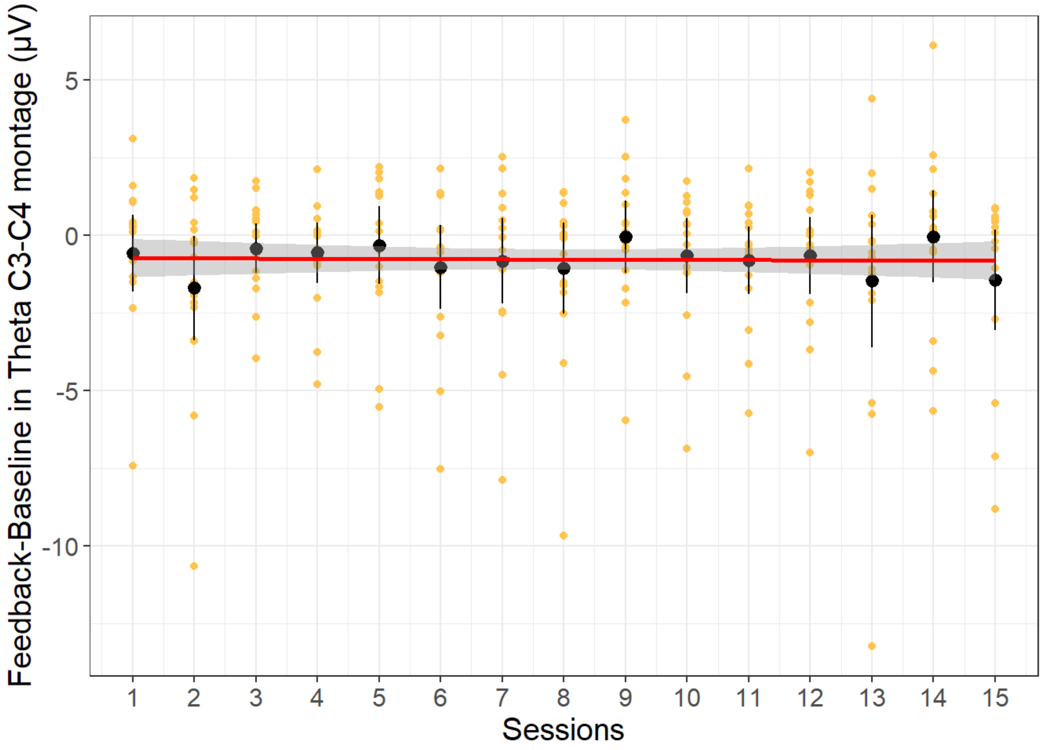


```
#-------------Beta_C3_C4 diff--------------------
windowsFonts("Arial" = windowsFont("Arial"))
ggplot(NFT, aes(Session, Beta_C3_C4_diff))+ geom_point(aes(y=Beta_C3_C4_diff),color="#fec44f",position =position_dodge(width = 0.5))+ stat_summary(fun = mean, geom = "line") + stat_summary(fun = mean, geom = "point", aes(group = Session))+ labs(x = "Session", y = "Feedback-Baseline in Beta C3-C4 montage (µV)")+ scale_x_continuous(name="Sessions", limits=c(1, 15), breaks = c(1,2,3,4,5,6,7,8,9,10,11,12,13,14,15)) + theme_bw()+ theme(text = element_text(size = 15, family = "Arial"))+stat_summary(fun.data = mean_cl_normal, geom = "pointrange")+ geom_smooth(method = "lm", colour = "Red")
```

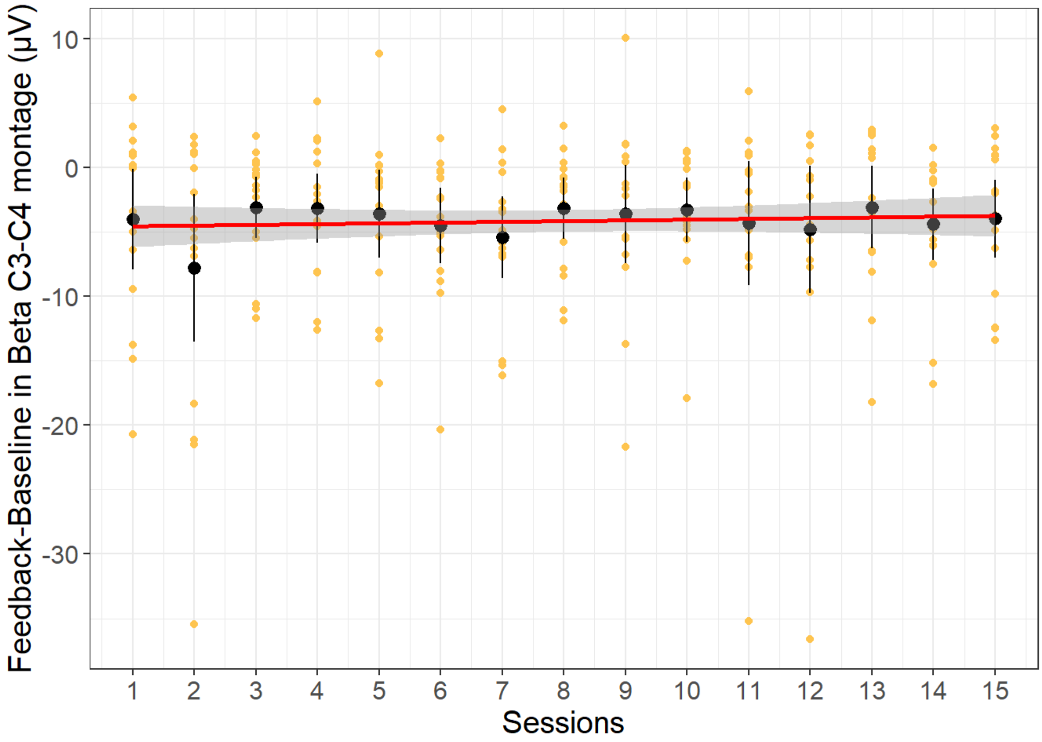


```
#-------------EMG_C3_C4 diff--------------------
windowsFonts("Arial" = windowsFont("Arial"))
ggplot(NFT, aes(Session,EMG_C3_C4_diff))+ geom_point(aes(y=EMG_C3_C4_diff),color="#fec44f",position =position_dodge(width = 0.5))+ stat_summary(fun = mean, geom = "line") + stat_summary(fun = mean, geom = "point", aes(group = Session))+ labs(x = "Session", y = "Feedback-Baseline in EMG C3-C4 montage (µV)")+ scale_x_continuous(name="Sessions", limits=c(1, 15), breaks = c(1,2,3,4,5,6,7,8,9,10,11,12,13,14,15)) + theme_bw()+ theme(text = element_text(size = 15, family = "Arial"))+stat_summary(fun.data = mean_cl_normal, geom = "pointrange")+ geom_smooth(method = "lm", colour = "Red")
```

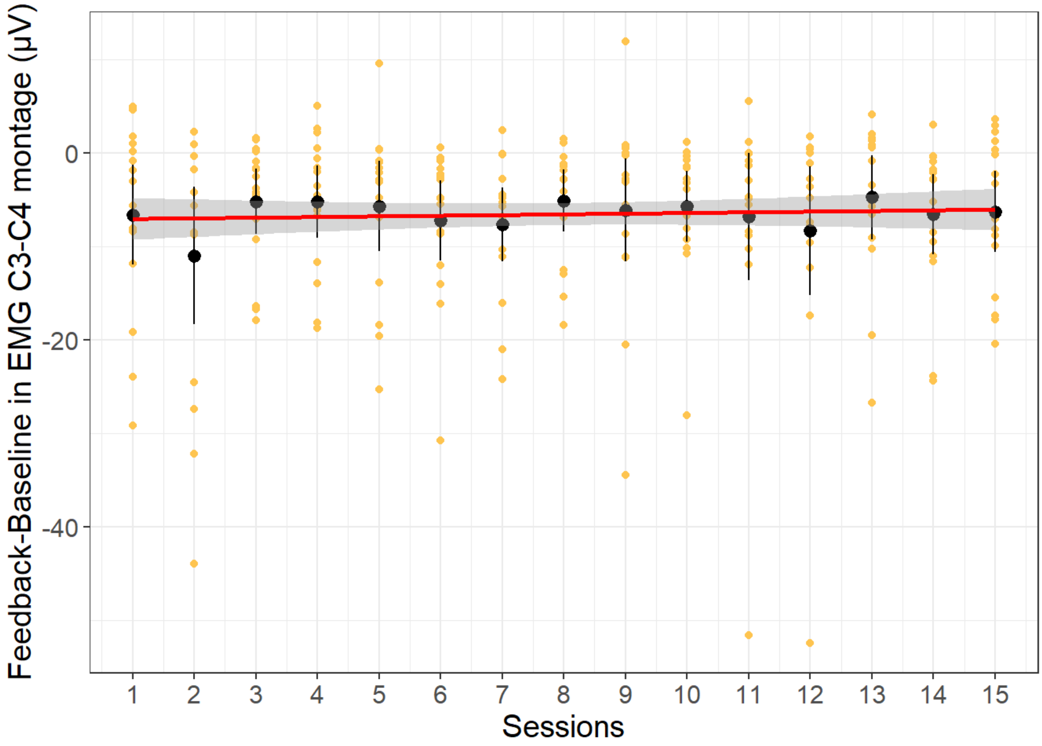


```
ggsave("C:/Users/dastg/Desktop/Main-Projects/Empra_2019_Dastgheib/Statistical_Analysis/Input/15VP-NFT/Output/Plots/NFT-EMG-C3-C4-F.png", width = 8, height = 4, dpi = 300)
```

Neurofeedback data device analysis,ANOVAs

```
#----------NFT sessions ANOVAS---------------------
#work with reshaped data:
NFTre<-read.csv(file=("C:/Users/dastg/Desktop/Main-Projects/Empra_2019_Dastgheib/Statistical_Analysis/Input/15VP-NFT/NFT_
reshaped_SC3C4.csv"),header = TRUE)

NFTmodel1<-aov_ez( "VP","Amplitude",NFTre,within=c("Session","State","Channel"),fun_aggregate=mean,na.rm=TRUE)

names(NFTmodel1)
```

```
## [1] "anova_table" "aov"         "Anova"       "lm"          "data"
```

```
summary(NFTmodel1)
```

```
##
## Univariate Type III Repeated-Measures ANOVA Assuming Sphericity
##
##                        Sum Sq num Df Error SS den Df  F value    Pr(>F)
## (Intercept)             67941      1   4503.8     15 226.2776 1.865e-10 ***
## Session                    63     14    631.5    210   1.5002   0.11286
## State                       2      1    114.8     15   0.3232   0.57813
## Channel                     0      1    107.0     15   0.0684   0.79725
## Session:State              22     14    197.7    210   1.6414   0.07036 .
## Session:Channel             8     14    135.8    210   0.8493   0.61497
## State:Channel               0      1      9.0     15   0.2329   0.63633
## Session:State:Channel       5     14     74.4    210   1.0137   0.44072
## ---
## Signif. codes:  0 '***' 0.001 '**' 0.01 '*' 0.05 '.' 0.1 ' ' 1
##
##
## Mauchly Tests for Sphericity
##
##                        Test statistic    p-value
## Session                    3.7304e-09 9.2740e-07
## Session:State              1.5406e-08 2.3288e-05
## Session:Channel            4.0536e-09 1.1291e-06
## Session:State:Channel      2.1610e-09 2.4870e-07
##
##
## Greenhouse-Geisser and Huynh-Feldt Corrections
##  for Departure from Sphericity
##
##                         GG eps Pr(>F[GG])
## Session               0.35521     0.2003
## Session:State         0.39910     0.1507
## Session:Channel       0.31187     0.5074
## Session:State:Channel 0.28807     0.4079
##
##                           HF eps Pr(>F[HF])
## Session               0.5525509  0.1669707
## Session:State         0.6632311  0.1067181
## Session:Channel       0.4558183  0.5409616
## Session:State:Channel 0.4072512  0.4199580
```

```
NFTre<-read.csv(file=("C:/Users/dastg/Desktop/Main-Projects/Empra_2019_Dastgheib/Statistical_Analysis/Input/15VP-NFT/NFT_
reshaped_CoC3C4.csv"),header = TRUE)


NFTmodel2<-aov_ez( "VP","Amplitude",NFTre,within=c("Freq","Session","State"),fun_aggregate=mean,na.rm=TRUE)

names(NFTmodel2)
```

```
## [1] "anova_table" "aov"         "Anova"       "lm"          "data"
```

```
summary(NFTmodel2)
```

```
##
## Univariate Type III Repeated-Measures ANOVA Assuming Sphericity
##
##                    Sum Sq num Df Error SS den Df  F value    Pr(>F)
## (Intercept)        896718      1  19058.2     15 705.7751 4.987e-14 ***
## Freq                76394      3  13565.7     45  84.4714 < 2.2e-16 ***
## Session               599     14   8342.7    210   1.0776  0.379475
## State                4101      1   3835.4     15  16.0393  0.001148 **
## Freq:Session          639     42   6843.0    630   1.4001  0.051307 .
## Freq:State           3200      3   2292.4     45  20.9399 1.220e-08 ***
## Session:State         322     14   5779.6    210   0.8345  0.631152
## Freq:Session:State    201     42   4371.6    630   0.6889  0.933006
## ---
## Signif. codes:  0 '***' 0.001 '**' 0.01 '*' 0.05 '.' 0.1 ' ' 1
##
##
## Mauchly Tests for Sphericity
##
##               Test statistic   p-value
## Freq                0.281664 0.0041993
## Session             0.000000 0.0003984
## Freq:State          0.005573 0.0000000
## Session:State       0.000000 0.0001494
##
##
## Greenhouse-Geisser and Huynh-Feldt Corrections
##  for Departure from Sphericity
##
##                GG eps Pr(>F[GG])
## Freq          0.56532  2.199e-11 ***
## Session       0.40567  0.3813299
## Freq:State    0.36038  0.0002378 ***
## Session:State 0.38596  0.5366062
## ---
## Signif. codes:  0 '***' 0.001 '**' 0.01 '*' 0.05 '.' 0.1 ' ' 1
##
##                  HF eps   Pr(>F[HF])
## Freq          0.6297707 1.914691e-12
## Session       0.6810693 3.831415e-01
## Freq:State    0.3663676 2.164987e-04
## Session:State 0.6286094 5.833983e-01
```

```
capture.output(NFTmodel2, file = "C:/Users/dastg/Desktop/Main-Projects/Empra_2019_Dastgheib/Statistical_Analysis/Input/15VP-NFT/Output/Tests/ANOVA_allfreq_CC3C4.txt")

F_to_eta2(f = c(1.40),
  df = c(42),
  df_error = c(630)) #0.52
```

Eta2_partial <dbl>	CI <dbl>	CI_low <dbl>	CI_high <dbl>
0.08536585	0.95	0	1

1 row

```
lsmeans(NFTmodel2, pairwise~State, adjust="tukey")
```

```
## $lsmeans
##  State    lsmean    SE df lower.CL upper.CL
##  Baseline   23.1 1.000 15     20.9     25.2
##  Feedback   20.1 0.767 15     18.5     21.8
##
## Results are averaged over the levels of: Session, Freq
## Confidence level used: 0.95
##
## $contrasts
##  contrast            estimate   SE df t.ratio p.value
##  Baseline - Feedback     2.92 0.73 15   4.005  0.0011
##
## Results are averaged over the levels of: Session, Freq
```

```
lsmeans(NFTmodel2, pairwise~Freq, adjust="tukey")
```

```
## $lsmeans
##  Freq      lsmean    SE df lower.CL upper.CL
##  High_Beta   28.3 1.198 15     25.7     30.8
##  Mu          16.8 1.117 15     14.4     19.2
##  Theta       14.0 0.727 15     12.4     15.5
##  Beta        27.4 1.148 15     25.0     29.8
##
## Results are averaged over the levels of: State, Session
## Confidence level used: 0.95
##
## $contrasts
##  contrast          estimate    SE df t.ratio p.value
##  High_Beta - Mu      11.438 1.520 15   7.525  <.0001
##  High_Beta - Theta   14.300 1.200 15  11.915  <.0001
##  High_Beta - Beta     0.864 0.676 15   1.278  0.5898
##  Mu - Theta           2.863 0.832 15   3.442  0.0170
##  Mu - Beta          -10.574 1.228 15  -8.611  <.0001
##  Theta - Beta       -13.437 1.063 15 -12.643  <.0001
##
## Results are averaged over the levels of: State, Session
## P value adjustment: tukey method for comparing a family of 4 estimates
```

```
lsmeans(NFTmodel2, pairwise~Freq:State, adjust="tukey")
```

```
## $lsmeans
##  Freq      State    lsmean    SE df lower.CL upper.CL
##  High_Beta Baseline   31.5 1.820 15     27.7     35.4
##  Mu        Baseline   16.9 1.057 15     14.7     19.2
##  Theta     Baseline   14.3 0.768 15     12.7     16.0
##  Beta      Baseline   29.5 1.435 15     26.4     32.5
##  High_Beta Feedback   25.0 0.769 15     23.3     26.6
##  Mu        Feedback   16.7 1.201 15     14.2     19.3
##  Theta     Feedback   13.6 0.749 15     12.0     15.2
##  Beta      Feedback   25.3 1.043 15     23.1     27.5
##
## Results are averaged over the levels of: Session
## Confidence level used: 0.95
##
## $contrasts
##  contrast                                estimate    SE df t.ratio p.value
##  High_Beta Baseline - Mu Baseline          14.609 1.993 15   7.329  0.0001
##  High_Beta Baseline - Theta Baseline       17.186 1.739 15   9.883  <.0001
##  High_Beta Baseline - Beta Baseline         2.056 0.845 15   2.433  0.2932
##  High_Beta Baseline - High_Beta Feedback    6.548 1.438 15   4.553  0.0069
##  High_Beta Baseline - Mu Feedback          14.814 2.184 15   6.782  0.0001
##  High_Beta Baseline - Theta Feedback       17.963 1.825 15   9.845  <.0001
##  High_Beta Baseline - Beta Feedback         6.219 1.721 15   3.614  0.0401
##  Mu Baseline - Theta Baseline               2.576 0.895 15   2.879  0.1464
##  Mu Baseline - Beta Baseline              -12.553 1.445 15  -8.686  <.0001
##  Mu Baseline - High_Beta Feedback          -8.062 1.107 15  -7.284  0.0001
##  Mu Baseline - Mu Feedback                  0.205 0.356 15   0.575  0.9987
##  Mu Baseline - Theta Feedback               3.354 0.667 15   5.026  0.0029
##  Mu Baseline - Beta Feedback               -8.391 1.041 15  -8.062  <.0001
##  Theta Baseline - Beta Baseline           -15.129 1.307 15 -11.577  <.0001
##  Theta Baseline - High_Beta Feedback      -10.638 0.973 15 -10.928  <.0001
##  Theta Baseline - Mu Feedback              -2.372 1.100 15  -2.156  0.4259
##  Theta Baseline - Theta Feedback            0.777 0.428 15   1.814  0.6210
##  Theta Baseline - Beta Feedback           -10.967 1.111 15  -9.867  <.0001
##  Beta Baseline - High_Beta Feedback         4.491 0.965 15   4.655  0.0057
##  Beta Baseline - Mu Feedback               12.758 1.657 15   7.698  <.0001
##  Beta Baseline - Theta Feedback            15.907 1.362 15  11.680  <.0001
##  Beta Baseline - Beta Feedback              4.162 1.013 15   4.109  0.0159
##  High_Beta Feedback - Mu Feedback           8.266 1.211 15   6.824  0.0001
##  High_Beta Feedback - Theta Feedback       11.415 0.845 15  13.516  <.0001
##  High_Beta Feedback - Beta Feedback        -0.329 0.561 15  -0.587  0.9985
##  Mu Feedback - Theta Feedback               3.149 0.788 15   3.997  0.0197
##  Mu Feedback - Beta Feedback               -8.595 1.125 15  -7.638  <.0001
##  Theta Feedback - Beta Feedback           -11.744 0.964 15 -12.178  <.0001
##
## Results are averaged over the levels of: Session
## P value adjustment: tukey method for comparing a family of 8 estimates
```

```
interFreqSess<-lsmeans(NFTmodel2, pairwise~Freq:Session, adjust="tukey")
capture.output(interFreqSess, file = "C:/Users/dastg/Desktop/Main-Projects/Empra_2019_Dastgheib/Statistical_Analysis/Input/15VP-NFT/Output/Tests/TukeyFreq-Session.txt")

#Effectsize for the main effect of session
library(effectsize)

F_to_eta2(f = c(1.08),
  df = c(5.68),
  df_error = c(85.19)) #0.07
```

Eta2_partial <dbl>	CI <dbl>	CI_low <dbl>	CI_high <dbl>
0.06717153	0.95	0	1

1 row

```
#Effectsize for the main effect of state
library(effectsize)

F_to_eta2(f = c(16.04),
  df = c(1),
  df_error = c(15)) #0.52
```

Eta2_partial <dbl>	CI <dbl>	CI_low <dbl>	CI_high <dbl>
0.5167526	0.95	0.1974272	1

1 row

```
#calculate the effect sizes:

library(effectsize)

F_to_eta2(f = c(2.4842),
  df = c(2),
  df_error = c(30))
```

Eta2_partial <dbl>	CI <dbl>	CI_low <dbl>	CI_high <dbl>
0.1420826	0.95	0	1

1 row

```
#=0.14
```

Further investigations on the interaction; separate ANOVAs for every rhythm

```
NFTre<-read.csv(file=("C:/Users/dastg/Desktop/Main-Projects/Empra_2019_Dastgheib/Statistical_Analysis/Input/15VP-NFT/NFT_reshaped_CoC3C4.csv"),header = TRUE)


#Theta----

NFTT<-NFTre %>% filter(Freq=="Theta")

NFTmodelT<-aov_ez( "VP","Amplitude",NFTT,within=c("Session","State"),fun_aggregate=mean,na.rm=TRUE)

names(NFTmodelT)
```

```
## [1] "anova_table" "aov"         "Anova"       "lm"          "data"
```

```
summary(NFTmodelT)
```

```
##
## Univariate Type III Repeated-Measures ANOVA Assuming Sphericity
##
##                Sum Sq num Df Error SS den Df  F value    Pr(>F)
## (Intercept)    93560      1   3809.3     15 368.4135 5.721e-12 ***
## Session           48     14    767.6    210   0.9289   0.52856
## State             73      1    330.5     15   3.2911   0.08971 .
## Session:State     27     14    415.7    210   0.9909   0.46369
## ---
## Signif. codes:  0 '***' 0.001 '**' 0.01 '*' 0.05 '.' 0.1 ' ' 1
##
##
## Mauchly Tests for Sphericity
##
##               Test statistic    p-value
## Session           2.0260e-09 0.00000021
## Session:State     1.3909e-07 0.00179417
##
##
## Greenhouse-Geisser and Huynh-Feldt Corrections
##  for Departure from Sphericity
##
##                GG eps Pr(>F[GG])
## Session       0.34094     0.4644
## Session:State 0.42436     0.4360
##
##                  HF eps Pr(>F[HF])
## Session       0.5194252  0.4897455
## Session:State 0.7337505  0.4546947
```

```
#Mu----
NFTMu<-NFTre %>% filter(Freq=="Mu")

NFTmodelmu<-aov_ez( "VP","Amplitude",NFTMu,within=c("Session","State"),fun_aggregate=mean,na.rm=TRUE)

names(NFTmodelmu)
```

```
## [1] "anova_table" "aov"         "Anova"       "lm"          "data"
```

```
summary(NFTmodelmu)
```

```
##
## Univariate Type III Repeated-Measures ANOVA Assuming Sphericity
##
##                Sum Sq num Df Error SS den Df  F value    Pr(>F)
## (Intercept)   135859      1   8989.9     15 226.6857 1.842e-10 ***
## Session          126     14   1257.5    210   1.5057   0.11083
## State              5      1    228.0     15   0.3307   0.57377
## Session:State     42     14    399.5    210   1.5848   0.08531 .
## ---
## Signif. codes:  0 '***' 0.001 '**' 0.01 '*' 0.05 '.' 0.1 ' ' 1
##
##
## Mauchly Tests for Sphericity
##
##               Test statistic    p-value
## Session           4.3328e-09 1.3060e-06
## Session:State     3.0157e-08 9.5154e-05
##
##
## Greenhouse-Geisser and Huynh-Feldt Corrections
##  for Departure from Sphericity
##
##                GG eps Pr(>F[GG])
## Session       0.35684     0.1983
## Session:State 0.39544     0.1670
##
##                  HF eps Pr(>F[HF])
## Session       0.5564270  0.1644187
## Session:State 0.6534631  0.1242563
```

```
#Beta----

NFTB<-NFTre %>% filter(Freq=="Beta")

NFTmodelB<-aov_ez( "VP","Amplitude",NFTB,within=c("Session","State"),fun_aggregate=mean,na.rm=TRUE)

names(NFTmodelB)
```

```
## [1] "anova_table" "aov"         "Anova"       "lm"          "data"
```

```
summary(NFTmodelB)
```

```
##
## Univariate Type III Repeated-Measures ANOVA Assuming Sphericity
##
##                Sum Sq num Df Error SS den Df  F value    Pr(>F)
## (Intercept)   360311      1   9489.6     15 569.5331 2.406e-13 ***
## Session          366     14   4609.7    210   1.1917 0.2834001
## State           2079      1   1847.0     15  16.8838 0.0009292 ***
## Session:State    170     14   3226.6    210   0.7890 0.6805948
## ---
## Signif. codes:  0 '***' 0.001 '**' 0.01 '*' 0.05 '.' 0.1 ' ' 1
##
##
## Mauchly Tests for Sphericity
##
##               Test statistic    p-value
## Session           2.1718e-09 2.4885e-07
## Session:State     7.6184e-10 1.7926e-08
##
##
## Greenhouse-Geisser and Huynh-Feldt Corrections
##  for Departure from Sphericity
##
##                GG eps Pr(>F[GG])
## Session       0.38381     0.3202
## Session:State 0.36456     0.5631
##
##                  HF eps Pr(>F[HF])
## Session       0.6230784  0.3064899
## Session:State 0.5749712  0.6139978
```

```
#highbeta----

NFTHB<-NFTre %>% filter(Freq=="High_Beta")

NFTmodelHB<-aov_ez( "VP","Amplitude",NFTHB,within=c("Session","State"),fun_aggregate=mean,na.rm=TRUE)

names(NFTmodelHB)
```

```
## [1] "anova_table" "aov"         "Anova"       "lm"          "data"
```

```
summary(NFTmodelHB)
```

```
##
## Univariate Type III Repeated-Measures ANOVA Assuming Sphericity
##
##               Sum Sq num Df Error SS den Df  F value    Pr(>F)
## (Intercept)   383383      1  10335.0     15 556.4315 2.852e-13 ***
## Session          698     14   8551.0    210   1.2246 0.2591002
## State           5145      1   3722.4     15  20.7324 0.0003807 ***
## Session:State    283     14   6109.4    210   0.6947 0.7784016
## ---
## Signif. codes:  0 '***' 0.001 '**' 0.01 '*' 0.05 '.' 0.1 ' ' 1
##
##
## Mauchly Tests for Sphericity
##
##               Test statistic    p-value
## Session           1.4419e-08 1.9938e-05
## Session:State     3.1574e-09 6.1580e-07
##
##
## Greenhouse-Geisser and Huynh-Feldt Corrections
##  for Departure from Sphericity
##
##                GG eps Pr(>F[GG])
## Session       0.35104     0.3064
## Session:State 0.35992     0.6302
##
##                  HF eps Pr(>F[HF])
## Session       0.5427371  0.2926629
## Session:State 0.5637860  0.6936837
```

cite R

```
#--------Cite R----------

citation()
```

```
##
## To cite R in publications use:
##
##   R Core Team (2023). R: A language and environment for statistical
##   computing. R Foundation for Statistical Computing, Vienna, Austria.
##   URL https://www.R-project.org/.
##
## Ein BibTeX-Eintrag für LaTeX-Benutzer ist
##
##   @Manual{,
##     title = {R: A Language and Environment for Statistical Computing},
##     author = {{R Core Team}},
##     organization = {R Foundation for Statistical Computing},
##     address = {Vienna, Austria},
##     year = {2023},
##     url = {https://www.R-project.org/},
##   }
##
## We have invested a lot of time and effort in creating R, please cite it
## when using it for data analysis. See also 'citation("pkgname")' for
## citing R packages.
```
